# Carboxylate-Catalyzed *C*-Silylation of Terminal Alkynes

**DOI:** 10.1021/acs.orglett.3c04213

**Published:** 2024-03-01

**Authors:** Anton Bannykh, Petri M. Pihko

**Affiliations:** Department of Chemistry and NanoScience Center, University of Jyväskylä, P.O.B. 35, FI-40014 University of Jyväskylä, Finland

## Abstract

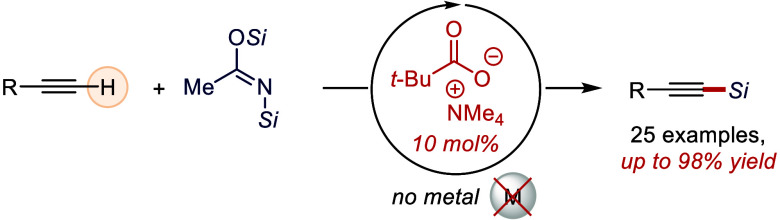

A carboxylate-catalyzed, metal-free *C*-silylation protocol for terminal alkynes is reported using a quaternary ammonium pivalate as the catalyst and commercially available *N*,*O*-bis(silyl)acetamides as silylating agents. The reaction proceeds under mild conditions, tolerates a range of functionalities, and enables concomitant *O*- or *N*-silylation of acidic OH or NH groups. A Hammett ρ value of +1.4 ± 0.1 obtained for *para*-substituted 2-arylalkynes is consistent with the proposed catalytic cycle involving a turnover-determining deprotonation step.

Metal-free deprotonation of hydrocarbons is challenging due to the high p*K*_a_ values of most hydrocarbons,^1^ and typical methods to generate carbanions with strong organometallic bases result in the formation of another organometallic species. For example, aromatic hydrocarbons can be deprotonated only by strong bases (e.g., Schlosser reagent)^2−4^ unless they are activated by a directing group.^5−7^ With a p*K*_a_ of ca. 28 (in DMSO), terminal alkynes might be an exception to this rule, but in practice even they require the use of strong organometallic bases and/or more electropositive, π-coordinating metals such as Zn.^8,9^ Catalytic deprotonation reactions of alkynes without metals are presumed to be highly challenging,^10^ although reactions with aldehydes and ketones (Favorskii reaction) have been realized with strong metal-free bases such as quaternary ammonium hydroxides.^11−13^

We have previously shown that metal-free catalytic enoyl isomerization^14^ and silylative aldol reactions^15^ are possible with simple carboxylate salt catalysts, without the need of metal or strong (and potentially nucleophilic) hydroxide bases. In the aldol reaction, the combination of tetramethylammonium pivalate (TMAP) and the neutral silylating agent *N*,*O*-bis(trimethylsilyl)acetamide (BSA) was required for rapid turnover rates. Herein we show that catalytic deprotonation of terminal alkynes with concomitant *C*-silylation can be achieved under very mild conditions using a metal-free carboxylate catalyst (Scheme 1) and silylamides as the silyl source.

**Scheme 1:**
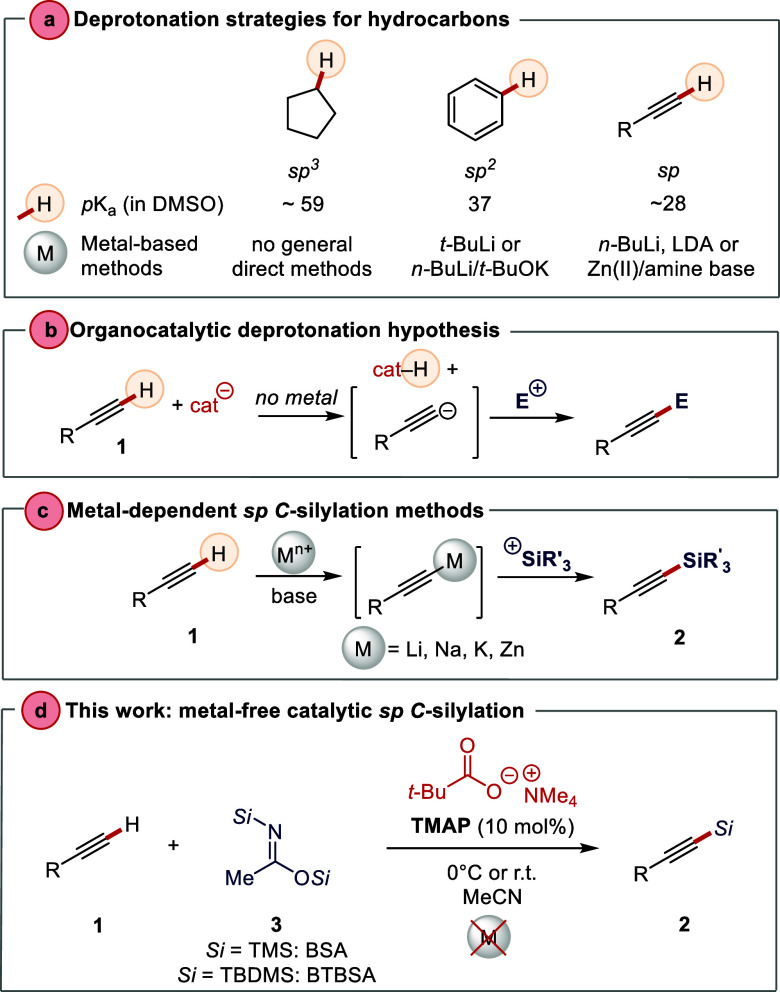
Deprotonation of Hydrocarbons: The Concept of Metal-Free Deprotonation–Silylation Sequence

Silylated terminal alkynes are versatile precursors of alkynyl nucleophiles in synthetic organic chemistry,^16−18^ and the silyl group also plays a role of a protecting group. Typical approaches to the synthesis of *C*-silylated alkynes include deprotonation of terminal alkynes with stoichiometric amount of organolithium compounds (e.g., *n*-BuLi) and the use of halosilanes as the silylating agent.^19^ An alternative silylation method with stoichiometric Lewis acid (ZnCl_2_) has been reported using silylamines,^20^ and the more reactive Zn(OTf)_2_ has been used as a Lewis acid in stoichiometric and catalytic variants employing halosilanes^21^ and silyl triflates,^22^ respectively. More recent catalytic versions of TMS protection employing the Ruppert–Prakash reagent (TMSCF_3_)^23^ and bis(trimethylsilyl)acetylene as the electrophilic TMS donor^24^ with strong bases, NaH and KHMDS, have also been reported recently. Catalytic decarboxylations of silyl alkynoates have been reported as an alternative pathway to silylalkynes.^25,26^ Silyl hydrides can also be used as silylating agents with alkali-metal hydroxides or transition metals as catalysts.^27−30^

We initiated our study by using phenylacetylene (**1a**) and *p*-CF_3_-phenylacetylene (**1b**) as model substrates and exposing these alkynes to a catalytic amount of TMAP and 1.5 equiv of BSA. To our delight, both substrates were converted to the desired TMS–acetylenes (R = H, **2a** or R = CF_3_, **2b**) in high yields (Table 1, entries 1 and 2). With **1b**, the reaction proceeded at −10 °C in nearly quantitative yield (94% **2b** was obtained). Deviations in catalyst loading or the quantity of BSA did not lead to any improvement (Table 1, entries 3–5), but in the absence of the catalyst (TMAP), no **2a** was detected (Table 1, entry 6). Interestingly, replacing the silylating agent with BSTFA gave no reaction (Table 1, entry 7), but the bulkier *tert*-butyldimethylsilylating agent BTBSA afforded the corresponding TBDMS-protected alkyne **4** in 65% yield.

**Table 1:**
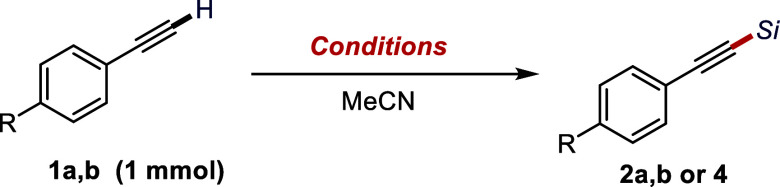

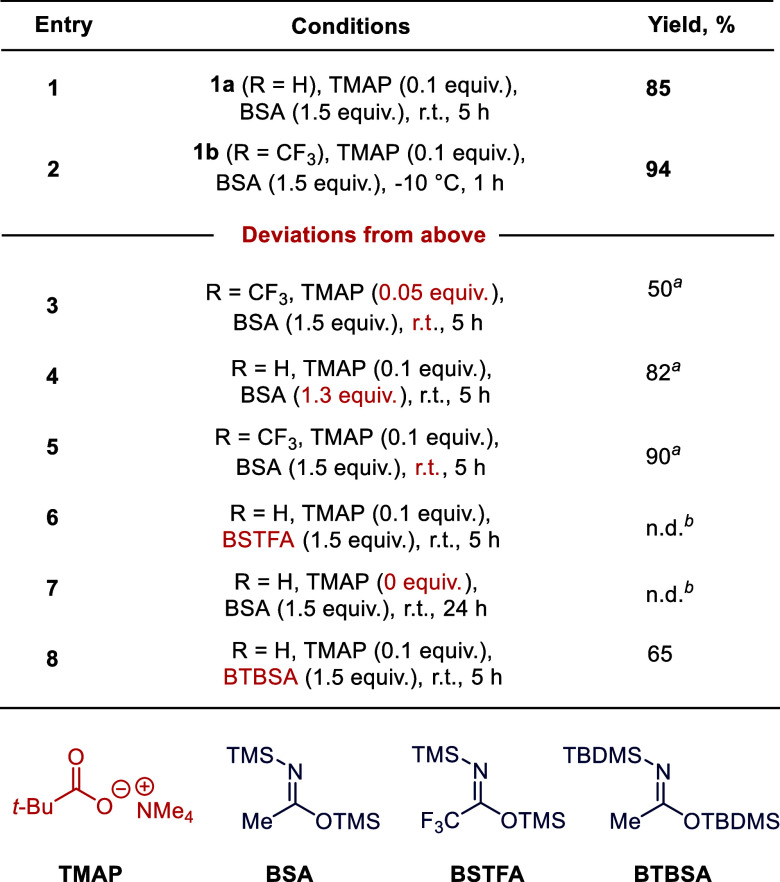
Optimization of the TMAP-Catalyzed Silylation of Alkynes^a^

a Conversion based on ^1^H NMR analysis of the crude reaction mixture.

b Run as an ^1^H NMR experiment in MeCN-*d*_3_.

The utility of the carboxylate–BSA silylating protocol was then explored with a range of substrates. Substituted phenylacetylenes **1a**–**i** gave the TMS-protected alkynes **2a**–**i** in excellent, even nearly quantitative yields with both electron-donating and electron-withdrawing groups (EWGs). Typically, the reactions proceeded to quantitative conversions, as judged by ^1^H NMR and/or TLC analysis of the crude reaction mixture. In general, EWG-substituted substrates **1b**, **1f**, and **1i** gave better yields when the reaction was conducted at −10 or 0 °C. Double silylation of **1u** was also readily achieved using 3 equiv of BSA, giving **2u** in 98% yield. Heterocyclic and other aromatic terminal alkynes **1j**–**m** also gave high yields of TMS-protected alkynes **2j**–**m**. The reaction also tolerated enynes and propargylic substrates bearing different functionalities and protecting groups (**1n**–**q**). With **1o**, a gram-scale experiment demonstrated that the process is scalable (91% yield of **2o** at 10 mmol scale vs 97% at 1 mmol scale).

The process also tolerates aliphatic alkynes **1r**–**t**, but with these, the reaction is more sluggish. With these substrates, reactions typically reached ca. 90% conversion, requiring additional purification. The desired TMS-protected alkynes **2r**–**t** can nevertheless be obtained in moderate isolated yields (52–70%) after purification.

The current catalytic BSA–TMAP system can also readily protect other hydroxy and amine groups in situ. To demonstrate the applicability of the silylation protocol with a complex substrate, we carried out a reaction with ethynylestradiol **1v** using an excess of BSA (7 equiv). The triply silylated product **2v**, with the TMS-protected phenol, tertiary alcohol, and terminal alkyne, was obtained in 84% yield (based on 90% sample purity). The triple silylation was unambiguously confirmed by scXRD (see the Supporting Information (SI); CCDC 2313380).

In addition, double *N*,*C*-silylation of Boc-propargylamine **1w** could be achieved in 95% yield. Recently, interest in N–H silylation protocols has been growing,^31−34^ although *N*-silylated compounds, especially those bearing an *N*-TMS group, are known to be relatively unstable.^35,36^ Indeed, spontaneous hydrolysis of the *N-*TMS group of **2w** during storage (4 °C) led to slow crystallization of the *C*-silylated carbamate **2x** (see Scheme 2). The scXRD structure of **2x** also confirmed the position of the *C*-silyl group (Scheme 2).

**Scheme 2:**
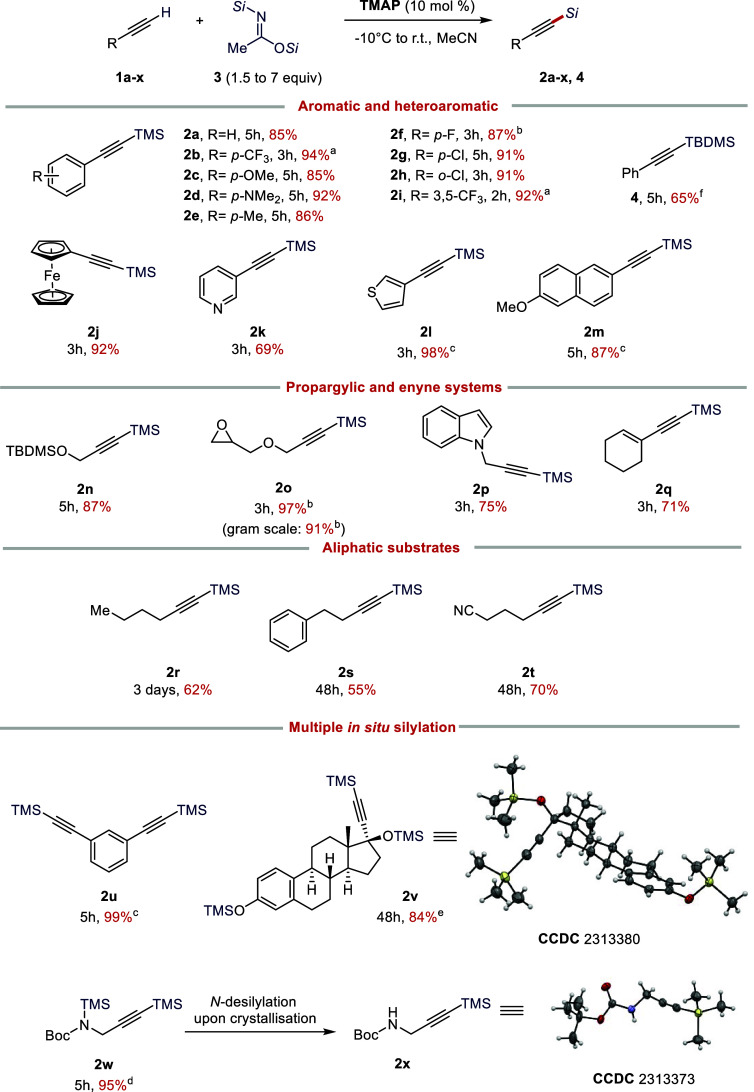
Scope of the Carboxylate-Catalyzed Silylation of Alkynes Reactions were carried out at r.t. with 1.5 equiv of BSA, unless otherwise noted: (a) run at −10 °C; (b) run at 0 °C; (c) 3 equiv of BSA was used; (d) 5 equiv of BSA was used; (e) 7 equiv of BSA and MeCN/THF (1:1 v/v) were used; (f) 1.5 equiv of BTBSA was used. See the Supporting Information for details.

Limitations of the present catalytic *C*-silylation method include the following examples (see Scheme 3). *N*-Tosyl-protected *N*-methylpropargylamine (**1y**) underwent partial isomerization to provide a poorly separable mixture of allene **5** and the desired TMS-protected alkyne **2y**. Attempts to perform double silylation for primary hydroxy group and terminal alkyne (**1z**, derived from 5-(hydroxymethyl)furfural) gave a mixture of mono- (**5z′**) and bis-silylated (**5z**) products in a 25:75 ratio, respectively, in a total yield of 50%. Finally, we noted that the phthalimide protecting group is not tolerated under the reaction conditions, and only decomposition of starting material **1aa** or **1ab** was observed.

**Scheme 3:**
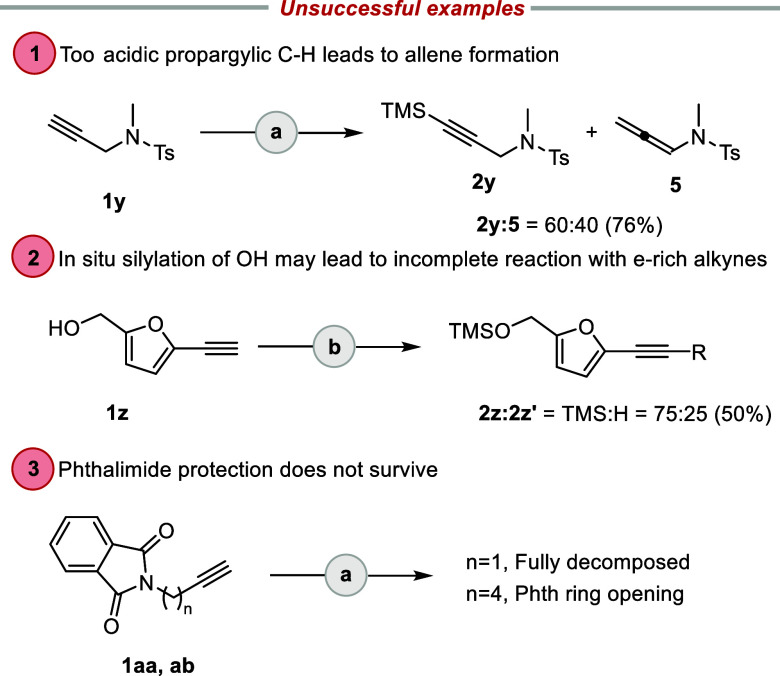
Unsuccessful Examples Reaction conditions: (a) TMAP (10 mol %), BSA (1.5 equiv), MeCN, 0 °C to r.t.; (b) TMAP (10 mol %), BSA (5 equiv), MeCN, 0 °C to r.t. Product ratios were determined by ^1^H NMR analysis.

Since control experiments without the TMAP catalyst (Table 1, entry 7) or with the alternative CF_3_-substituted silylating agent BSTFA (Table 1, entry 6) resulted in no reaction, the catalytic cycle appears to require both species. We propose a probase mechanism involving an initial silyl transfer from BSA to the pivalate anion of TMAP,^15^ leading to formation of anionic species **I** (Scheme 4)^37−40^ with subsequent deprotonation of the alkyne (Scheme 4). This mechanism is supported by the inertness of BSTFA, which should give rise to a weaker base. Furthermore, this mechanistic scenario also corroborated by the Hammett plot with different aryl-conjugated alkynes (**2a**, **2c**–**g**), which resulted in a ρ value of +1.4 ± 0.1 (see the SI). This value is consistent with the formation of carbanionic-like species in the turnover-determining deprotonation step and agrees with our initial mechanistic blueprint for the reaction.^41−43,20^ In the proposed catalytic cycle, the alkyne anion–Me_4_N^+^ ion pair **II** (Scheme 4)^10−12^ is silylated by BSA, generating the probase and completing the cycle. In the kinetic experiments with phenylacetylenes, 1 mol % TMAP catalyst was sufficient to give reasonable rates in ^1^H NMR studies (see the SI), but in preparative experiments, we found that using 10 mol % TMAP was a safer option to cover a broad range of substrates.

**Scheme 4:**
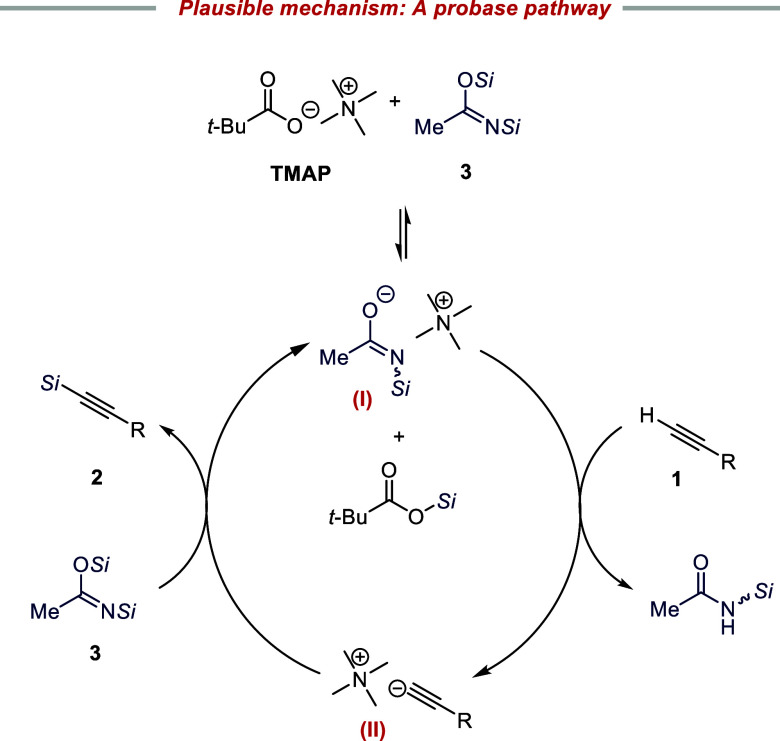
Plausible Reaction Mechanism

In conclusion, we report a new carboxylate-catalyzed, metal-free protocol for the silylation of terminal alkynes. A bench-stable, inexpensive catalyst (TMAP) and commercially available noncorrosive silylating agent (BSA or BTBSA) can be employed. The protocol tolerates a range of substrates, and unprotected OH and NH groups are typically silylated as well under the reaction conditions.^44^

## Data Availability

The data underlying this study are available in the published article and its Supporting Information.

## Supporting Information Available

Experimental procedures, details of kinetics experiments, and crystallographic data (PDF)
Copies of NMR spectra (PDF)
FAIR data, including the primary NMR FID files, for compounds **2a**–**2x** and **4** (ZIP)

## References

[ref1] Bordwell F. G. Equilibrium Acidities in Dimethyl Sulfoxide Solution. Acc. Chem. Res. 1988, 21 (12), 456–463. 10.1021/ar00156a004.

[ref2] Ohsato T.; Okuno Y.; Ishida S.; Iwamoto T.; Lee K.-H.; Lin Z.; Yamashita M.; Nozaki K. A Potassium Diboryllithate: Synthesis, Bonding Properties, and the Deprotonation of Benzene. Angew. Chem., Int. Ed. 2016, 55 (38), 11426–11430. 10.1002/anie.201605005. 27533099

[ref3] Schlosser M.; Jung H. C.; Takagishi S. Selective Mono- or Dimetalation of Arenes by Means of Superbasic Reagents. Tetrahedron 1990, 46 (16), 5633–5648. 10.1016/S0040-4020(01)87763-2.

[ref4] Bryce-Smith D.; Gold V.; Satchell D. P. N. The Hydrogen Isotope Effect in the Metallation of Benzene and Toluene. J. Chem. Soc. 1954, 2743–2747. 10.1039/jr9540002743.

[ref5] Beak P.; Meyers A. I. Stereo- and Regiocontrol by Complex Induced Proximity Effects: Reactions of Organolithium Compounds. Acc. Chem. Res. 1986, 19 (11), 356–363. 10.1021/ar00131a005.

[ref6] Snieckus V. Directed Ortho Metalation. Tertiary Amide and *O*-Carbamate Directors in Synthetic Strategies for Polysubstituted Aromatics. Chem. Rev. 1990, 90 (6), 879–933. 10.1021/cr00104a001.

[ref7] Schlosser M. The 2 × 3 Toolbox of Organometallic Methods for Regiochemically Exhaustive Functionalization. Angew. Chem., Int. Ed. 2005, 44 (3), 376–393. 10.1002/anie.200300645. 15558637

[ref8] Niwa S.; Soai K. Catalytic Asymmetric Synthesis of Optically Active Alkynyl Alcohols by Enantioselective Alkynylation of Aldehydes and by Enantioselective Alkylation of Alkynyl Aldehydes. J. Chem. Soc., Perkin Trans. 1 1990, 937 10.1039/p19900000937.

[ref9] Frantz D. E.; Fässler R.; Carreira E. M. Facile Enantioselective Synthesis of Propargylic Alcohols by Direct Addition of Terminal Alkynes to Aldehydes. J. Am. Chem. Soc. 2000, 122 (8), 1806–1807. 10.1021/ja993838z.

[ref10] Paria S.; Lee H.-J.; Maruoka K. Enantioselective Alkynylation of Isatin Derivatives Using a Chiral Phase-Transfer/Transition-Metal Hybrid Catalyst System. ACS Catal. 2019, 9 (3), 2395–2399. 10.1021/acscatal.8b04949.

[ref11] Ishikawa T.; Mizuta T.; Hagiwara K.; Aikawa T.; Kudo T.; Saito S. Catalytic Alkynylation of Ketones and Aldehydes Using Quaternary Ammonium Hydroxide Base. J. Org. Chem. 2003, 68 (9), 3702–3705. 10.1021/jo026592g. 12713383

[ref12] Weil T.; Schreiner P. R. Organocatalytic Alkynylation of Aldehydes and Ketones under Phase-Transfer Catalytic Conditions. Eur. J. Org. Chem. 2005, 2005 (11), 2213–2217. 10.1002/ejoc.200500064.

[ref13] Schmidt E. Yu.; Cherimichkina N. A.; Bidusenko I. A.; Protzuk N. I.; Trofimov B. A. Alkynylation of Aldehydes and Ketones Using the Bu_4_NOH/H_2_O/DMSO Catalytic Composition: A Wide-Scope Methodology. Eur. J. Org. Chem. 2014, 2014 (21), 4663–4670. 10.1002/ejoc.201402275.

[ref14] Riuttamäki S.; Laczkó G.; Madarász Á.; Földes T.; Pápai I.; Bannykh A.; Pihko P. M. Carboxylate Catalyzed Isomerization of β,γ-Unsaturated N-Acetylcysteamine Thioesters. Chem. - Eur. J. 2022, 28 (45), e202201030 10.1002/chem.202201030. 35604200 PMC9541288

[ref15] Riuttamäki S.; Bannykh A.; Pihko P. M. Carboxylate Catalysis: A Catalytic O-Silylative Aldol Reaction of Aldehydes and Ethyl Diazoacetate. J. Org. Chem. 2023, 88 (20), 14396–14403. 10.1021/acs.joc.3c01304. 37768196 PMC10594658

[ref16] Milzarek T. M.; Ramirez N. P.; Liu X.-Y.; Waser J. One-Pot Synthesis of Functionalized Bis(trifluoromethylated)benziodoxoles from Iodine(I) Precursors. Chem. Commun. 2023, 59 (84), 12637–12640. 10.1039/D3CC04525K. 37791867

[ref17] Larock R. C.; Yum E. K. Synthesis of Indoles via Palladium-Catalyzed Heteroannulation of Internal Alkynes. J. Am. Chem. Soc. 1991, 113 (17), 6689–6690. 10.1021/ja00017a059.

[ref18] Larson G. L. Some Aspects of the Chemistry of Alkynylsilanes. Synthesis 2018, 50 (13), 2433–2462. 10.1055/s-0036-1591979.

[ref19] Preparative Acetylenic Chemistry, 2nd ed.; Brandsma L., Ed.; Studies in Organic Chemistry, Vol. 34; Elsevier, 1988.

[ref20] Andreev A. A.; Konshin V. V.; Komarov N. V.; Rubin M.; Brouwer C.; Gevorgyan V. Direct Electrophilic Silylation of Terminal Alkynes. Org. Lett. 2004, 6 (3), 421–424. 10.1021/ol036328p. 14748608

[ref21] Jiang H.; Zhu S. Silylation of 1-Alkynes with Chlorosilanes Promoted by Zn(OTf)_2_: An Efficient Way to the Preparation of Alkynylsilanes. Tetrahedron Lett. 2005, 46 (3), 517–519. 10.1016/j.tetlet.2004.10.175.

[ref22] Rahaim R. J.; Shaw J. T. Zinc-Catalyzed Silylation of Terminal Alkynes. J. Org. Chem. 2008, 73 (7), 2912–2915. 10.1021/jo702557d. 18331056

[ref23] Arde P.; Reddy V.; Vijaya Anand R. NHC Catalysed Trimethylsilylation of Terminal Alkynes and Indoles with Ruppert’s Reagent under Solvent Free Conditions. RSC Adv. 2014, 4 (91), 49775–49779. 10.1039/C4RA08727E.

[ref24] Kuciński K.; Hreczycho G. Transition Metal-Free Catalytic C–H Silylation of Terminal Alkynes with Bis(Trimethylsilyl)Acetylene Initiated by KHMDS. ChemCatChem 2022, 14 (18), e202200794 10.1002/cctc.202200794.

[ref25] Kawatsu T.; Aoyagi K.; Nakajima Y.; Choi J.-C.; Sato K.; Matsumoto K. Catalytic Decarboxylation of Silyl Alkynoates to Alkynylsilanes. Organometallics 2020, 39 (16), 2947–2950. 10.1021/acs.organomet.0c00433.

[ref26] Kawatsu T.; Kataoka S.; Fukaya N.; Choi J.-C.; Sato K.; Matsumoto K. Fluoride Ion-Initiated Decarboxylation of Silyl Alkynoates to Alkynylsilanes. ACS Omega 2021, 6 (19), 12853–12857. 10.1021/acsomega.1c01256. 34056436 PMC8154224

[ref27] Toutov A. A.; Betz K. N.; Schuman D. P.; Liu W.-B.; Fedorov A.; Stoltz B. M.; Grubbs R. H. Alkali Metal-Hydroxide-Catalyzed C(Sp)–H Bond Silylation. J. Am. Chem. Soc. 2017, 139 (4), 1668–1674. 10.1021/jacs.6b12114. 28026952

[ref28] Stachowiak H.; Kuciński K.; Kallmeier F.; Kempe R.; Hreczycho G. Cobalt-Catalyzed Dehydrogenative C–H Silylation of Alkynylsilanes. Chem. - Eur. J. 2022, 28 (1), e202103629 10.1002/chem.202103629. 34634167 PMC9299208

[ref29] Wissing M.; Studer A. Tuning the Selectivity of AuPd Nanoalloys towards Selective Dehydrogenative Alkyne Silylation. Chem. - Eur. J. 2019, 25 (23), 5870–5874. 10.1002/chem.201900493. 30719758

[ref30] Voronkov M. G.; Ushakova N. I.; Tsykhanskaya I. I.; Pukhnarevich V. B. Dehydrocondensation of Trialkylsilanes with Acetylene and Monosubstituted Acetylenes. J. Organomet. Chem. 1984, 264 (1), 39–48. 10.1016/0022-328X(84)85131-1.

[ref31] Leland B. E.; Mondal J.; Trovitch R. J. Sustainable Preparation of Aminosilane Monomers, Oligomers, and Polymers through Si–N Dehydrocoupling Catalysis. Chem. Commun. 2023, 59 (25), 3665–3684. 10.1039/D2CC07092H. 36857645

[ref32] Kuciński K.; Hreczycho G. Silicon–Nitrogen Bond Formation via Dealkynative Coupling of Amines with Bis(trimethylsilyl)acetylene Mediated by KHMDS. Chem. Commun. 2022, 58 (81), 11386–11389. 10.1039/D2CC04413G. 36128699

[ref33] Liu M.-M.; Xu Y.; He C. Catalytic Asymmetric Dehydrogenative Si–H/N–H Coupling: Synthesis of Silicon-Stereogenic Silazanes. J. Am. Chem. Soc. 2023, 145 (21), 11727–11734. 10.1021/jacs.3c02263. 37204933

[ref34] Harinath A.; Karmakar H.; Kisan D. A.; Nayek H. P.; Panda T. K. NHC–Zn Alkyl Catalyzed Cross-Dehydrocoupling of Amines and Silanes. Org. Biomol. Chem. 2023, 21 (20), 4237–4244. 10.1039/D3OB00453H. 37139558

[ref35] Blokker E.; Sun X.; Poater J.; van der Schuur J. M.; Hamlin T. A.; Bickelhaupt F. M. The Chemical Bond: When Atom Size Instead of Electronegativity Difference Determines Trend in Bond Strength. Chem. - Eur. J. 2021, 27 (63), 15616–15622. 10.1002/chem.202103544. 34609774 PMC9298008

[ref36] Warner D. L.; Hibberd A. M.; Kalman M.; Klapars A.; Vedejs E. N-Silyl Protecting Groups for Labile Aziridines: Application toward the Synthesis of N–H Aziridinomitosenes. J. Org. Chem. 2007, 72 (22), 8519–8522. 10.1021/jo7013615. 17910500

[ref37] Claraz A.; Oudeyer S.; Levacher V. Chiral Quaternary Ammonium Aryloxide/*N*,*O*-Bis(Trimethylsilyl)acetamide Combination as Efficient Organocatalytic System for the Direct Vinylogous Aldol Reaction of (5*H*)-Furan-2-one Derivatives. Adv. Synth. Catal. 2013, 355 (5), 841–846. 10.1002/adsc.201201041.

[ref38] Tanaka J.; Suzuki S.; Tokunaga E.; Haufe G.; Shibata N. Asymmetric Desymmetrization via Metal-Free C–F Bond Activation: Synthesis of 3,5-Diaryl-5-fluoromethyloxazolidin-2-ones with Quaternary Carbon Centers. Angew. Chem., Int. Ed. 2016, 55 (32), 9432–9436. 10.1002/anie.201603210. 27332650

[ref39] Teng B.; Chen W.; Dong S.; Kee C. W.; Gandamana D. A.; Zong L.; Tan C.-H. Pentanidium- and Bisguanidinium-Catalyzed Enantioselective Alkylations Using Silylamide as Brønsted Probase. J. Am. Chem. Soc. 2016, 138 (31), 9935–9940. 10.1021/jacs.6b05053. 27447024

[ref40] Bourgeois D.; Craig D.; King N. P.; Mountford D. M. Synthesis of Homoallylic Sulfones through a Decarboxylative Claisen Rearrangement Reaction. Angew. Chem., Int. Ed. 2005, 44 (4), 618–621. 10.1002/anie.200462023. 15612061

[ref41] Saunders W. H.; Williams R. A. Mechanisms of Elimination Reactions. II. Rates of Elimination from Some Substituted 2-Phenylethyl Bromides and 2-Phenylethyldimethylsulfonium Bromides. J. Am. Chem. Soc. 1957, 79 (14), 3712–3716. 10.1021/ja01571a030.

[ref42] Ayrey G.; Bourns A. N.; Vyas V. A. Isotope Effect Studies on Elimination Reactions: III. Nitrogen Isotope Effects in the E2 Reaction of Ethyltrimethyl-Ammonium and 2-Phenylethyltrimethylammonium Ions. Can. J. Chem. 1963, 41 (7), 1759–1767. 10.1139/v63-253.

[ref43] Smith P. J.; Tsui S. K. Reactant-like Transition State for Concerted E2 Process. J. Am. Chem. Soc. 1973, 95 (14), 4760–4761. 10.1021/ja00795a052.

[ref44] Bannykh A.; Pihko P. Carboxylate Catalyzed Silylation of Alkynes. ChemRxiv 2023, 10.26434/chemrxiv-2023-wj8lc. PMC10949233 38428925

