# Supplementary material for: Carboxylate-Catalyzed C-Silylation of Terminal Alkynes
Source: Org Lett. 2024 Mar 1;26(10):1991–5. doi: 10.1021/acs.orglett.3c04213 (PMC10949233; doi:10.1021/acs.orglett.3c04213)
Supplement: Supplementary file 1 — ol3c04213_si_001.pdf [file ol3c04213_si_001.pdf]

Supporting Information

Carboxylate catalyzed *C*-silylation of terminal alkynes

Anton Bannykh, Petri M. Pihko *

Department of Chemistry and Nanoscience Center,. P.O.B. 35, 40014 University of Jyväskylä, FINLAND

E-mail: Petri.Pihko@jyu.fi

1 Contents

2 General Information 4

3 Experimental 5

3.1 Preparation of the tetramethylammonium pivalate catalyst 5

3.2 General procedure for the TMAP-catalyzed silylation of alkynes 6

3.2.1 Trimethyl(phenylethynyl)silane (**2a**) 6

3.2.2 Trimethyl((4-(trifluoromethyl)phenyl)ethynyl)silane (**2b**) 6

3.2.3 ((4-Methoxyphenyl)ethynyl)trimethylsilane (**2c**) 7

3.2.4 *N,N*-Dimethyl-4-((trimethylsilyl)ethynyl)aniline (**2d**) 7

3.2.5 Trimethyl(*p*-tolylethynyl)silane (**2e**) 8

3.2.6 ((4-Fluorophenyl)ethynyl)trimethylsilane (**2f**) 8

3.2.7 ((4-Chlorophenyl)ethynyl)trimethylsilane (**2g**) 8

3.2.8 ((2-Chlorophenyl)ethynyl)trimethylsilane (**2h**) 9

3.2.9 ((3,5-Bis(trifluoromethyl)phenyl)ethynyl)trimethylsilane (**2i**) 9

3.2.10 Ferrocenylethynetrimethylsilane (**2j**) 9

3.2.11 3-((Trimethylsilyl)ethynyl)pyridine (**2k**) 10

3.2.12 Trimethyl(thiophen-3-ylethynyl)silane (**2l**) 10

3.2.13 ((6-Methoxynaphthalen-2-yl)ethynyl)trimethylsilane (**2m**) 10

3.2.14 *tert*-Butyldimethyl((3-(trimethylsilyl)prop-2-yn-1-yl)oxy)silane (**2n**) 11

3.2.15 Trimethyl(3-(oxiran-2-ylmethoxy)prop-1-yn-1-yl)silane (**2o**) 11

3.2.16 1-(3-(Trimethylsilyl)prop-2-yn-1-yl)-*1H*-indole (**2p**) 12

3.2.17 (Cyclohex-1-en-1-ylethynyl)trimethylsilane (**2q**) 12

3.2.18 Hex-1-yn-1-yltrimethylsilane (**2r**) 12

3.2.19 Trimethyl(4-phenylbut-1-yn-1-yl)silane (**2s**) 13

3.2.20 6-(Trimethylsilyl)hex-5-ynenitrile (**2t**) 13

3.2.21 1,3-Bis((trimethylsilyl)ethynyl)benzene (**2u**) 13

3.2.22 *(((8R,9S,13S,14S,17S)*-13-Methyl-17-((trimethylsilyl)ethynyl)-7,8,9,11,12,13,14,15,16,17-decahydro-*6H*-cyclopenta[*a*]phenanthrene-3,17-diyl)bis(oxy))bis(trimethylsilane) (**2v**) 14

3.2.23 *tert*-Butyl (trimethylsilyl)(3-(trimethylsilyl)prop-2-yn-1-yl)carbamate (**2w**) 14

3.2.24 *tert*-Butyl (3-(trimethylsilyl)prop-2-yn-1-yl)carbamate (**2x**) 15

3.2.25 *tert*-Butyldimethyl(phenylethynyl)silane (**4**) 15

3.3 Mechanistic studies 16

3.3.1 Kinetic studies 16
3.3.2 Hammett plot 29
3.4 Crystallographic data 31
3.5 References 35

2 General Information

All reactions were carried out under an argon atmosphere using Schlenk technique in oven-dried glassware, unless otherwise noted. Schlenk line was additionally equipped with the Drierite™ gas-drying unit. Syringes, catalysts and moisture sensitive reagents (e.g. BSA) were stored in desiccators. When needed, nonaqueous reagents were transferred under argon *via* syringe or cannula and dried prior to use. Dry MeCN and THF were obtained by passing deoxygenated solvents through activated alumina columns (MBraun SPS-800 Series solvent purification system). Deionized ultrapure water was obtained by Milli-Q Synergy® Water Purification System. Other solvents and reagents were used as obtained from supplier, unless otherwise noted. The reactions were cooled either with ice-water baths (nominally 0 °C, external temperature) or with ice-water-rock salt mixtures (external bath temperature –10 °C).

Analytical TLC was performed using Merck silica gel F254 (230-400 mesh) plates and analyzed by UV light or by staining upon heating with $KMnO_4$ solution (1 g $KMnO_4$, 6.7 g K_2CO_3, 1.7 mL 1M NaOH, 100 mL H_2O). For silica gel chromatography, the flash chromatography technique was used, with Merck silica gel 60 (230-400 mesh) and p.a. grade solvents unless otherwise noted. Alkynes **1n**[1], **1p**[2], **1w**[3], **1y**[4], **1z**[5], **1aa**[6], **1ab**[7] were prepared according to the literature procedures.

The ^{1}H NMR and ^{13}C NMR spectra were recorded in $CDCl_3$ or CD_3CN on Bruker Avance 500 or 300 MHz spectrometers. The chemical shifts are reported in ppm relative to $CHCl_3$ (δ 7.26), CHD_2CN (δ 1.94) or ^{1}H NMR. For the ^{13}C NMR spectra, the residual $CDCl_3$ (δ 77.16), CD_3CN (δ 118.26) and were used as the internal standards. IR spectra were recorded on a Bruker Alpha FT-IR spectrometer. Optical rotations were obtained with a Perkin-Elmer 343 polarimeter. High resolution mass spectrometric data were measured using Agilent 6560 IM-QTOF mass spectrometer. Metal concentrations in solutions were analyzed by inductively coupled plasma optical emission spectrometer (ICP-OES, PerkinElmer, Optima 8300).

Single-crystal X-ray diffraction analyses were performed at 120 K on Rigaku XtaLAB Synergy R, HyPix-Arc 100 with CuKα (λ = 1.54184 Å) radiation. The data reduction and absorption corrections were made by program CrysAlisPro.[8] Crystals were obtained by slow evaporation of **2v** solution in $CDCl_3$ or by spontaneous decomposition of **2w** upon cooling leading to **2x**. The structures were solved by using SHELXT[9] in OleX 2-1.5[10] and refined with SHELXL.[11] The structure was drawn with Mercury.[12] Crystallographic data was deposited with the accession number 2313373 (**2v**) and 2313380 (**2x**) can be obtained free of charge from the Cambridge Crystallographic Data Centre via www.ccdc.cam.ac.uk/structures.

3 Experimental

3.1 Preparation of the tetramethylammonium pivalate catalyst

This catalyst is a known compound.[13] For the purposes of this study, the catalyst was prepared as follows: Tetramethylammonium hydroxide solution (25 wt-% in water, 3.41 mL, 866 mg, 9.5 mmol, 0.95 equiv) was added to a suspension of pivalic acid (1.02 g, 10.0 mmol, 1.0 equiv) in 5 mL of deionized water at room temperature. After 10 min, reaction mixture was concentrated under reduced pressure, and remaining water was removed by heating on the vacuum line using heat gun until the viscous liquid turns to white solid. Final drying was completed overnight under high vacuum at room temperature to give TMAP in quantitative yield (1.74 g) as a white solid. TMAP is slightly hygroscopic, and it was stored in desiccator over silica gel (orange indicator). However, no special precautions were used in handling and weighing the catalyst.

^{1}H NMR (300 MHz, CD_3CN) 3.19 (s, 12H), 1.02 (s, 9H).

^{13}C NMR (75 MHz, CD_3CN) 182.1, 55.9, 39.9, 29.6.

ICP OES: Ca (56 mg/kg) and K (68 mg/kg) were found. No other metals were detected above the detection limit of the instrument (typically 5-25 ppm).

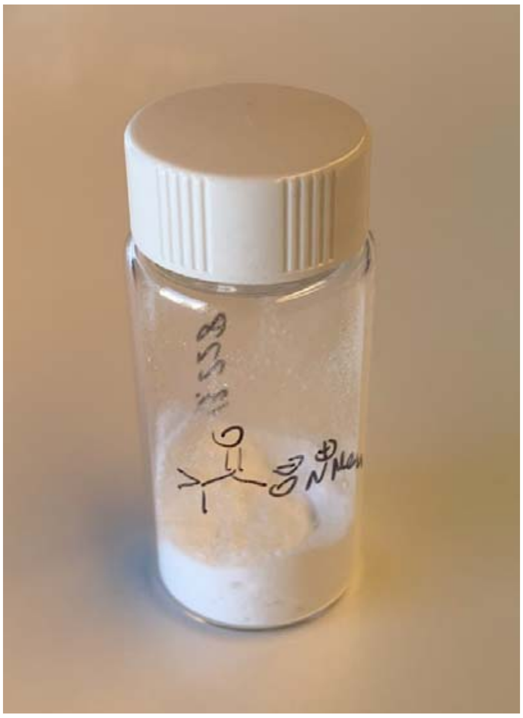

Image S1. A vial containing TMAP catalyst.

3.2 General procedure for the TMAP-catalyzed silylation of alkynes

*General procedure 1 (**GP1**):* To a stirred solution of TMAP (0.1 equiv) in dry MeCN, acetylene (0. 50 – 1.00 mmol, 1 equiv) was added either via syringe or as a solid, followed by addition of *N,O*-bis(trimethylsilyl)acetamide (typically 1.5 equiv) via syringe at room temperature. The reaction progress was followed by TLC (UV or $KMnO_4$). After no acetylene remained in the reaction mixture, hexane (10 ml) and water (15 mL) were added, the phases were separated, and the aqueous phase was extracted in hexane (10 ml × 3). The combined organic extracts were dried over Na_2SO_4, filtered through the 1-2 cm pad of silica gel, washed the pad with extra portion of hexane (10 ml) and concentrated to give the pure desired product as a colorless oil or solid, if otherwise noted.

*General procedure 2 (**GP2**):* To an cooled solution (0 °C) of TMAP (0.1 equiv) in dry MeCN, acetylene (0.50 – 1.00 mmol, 1 equiv) was added either via syringe or as a solid, followed by addition of *N,O*-bis(trimethylsilyl)acetamide (typically 1.5 equiv) via syringe. The reaction progress was followed by TLC (UV or $KMnO_4$). After no acetylene remained in the reaction mixture, hexanes (10 ml) and water (15 mL) were added, the phases were separated, and the aqueous phase was extracted with hexanes (10 ml × 3). The combined organic extracts were dried over Na_2SO_4, filtered through the 1-2 cm pad of silica gel, washed the pad with extra portion of hexane (10 ml) and concentrated to give the pure desired product as a colorless oil or solid, if otherwise noted.

3.2.1 Trimethyl(phenylethynyl)silane (**2a**)

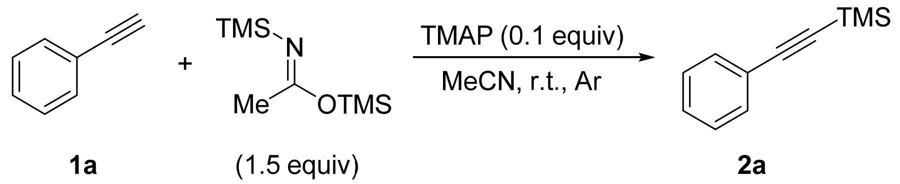

Prepared using **GP1** using phenylacetylene (110 µL, 102 mg, 1 mmol) to give, after 5 h reaction time, 150 mg (85 %) of **2a** as a colorless oil. Spectral data corresponds to previously published data.[14]

^{1}H NMR (300 MHz, $CDCl_3$) δ 7.57 – 7.41 (m, 2H), 7.35 – 7.27 (m, 3H), 0.27 (s, 9H). **^{13}C{^{1}H} NMR** (75 MHz, $CDCl_3$) δ 132.1, 128.6, 128.3, 123.3, 105.3, 94.2, 0.1. **IR** (neat, ATR): ν_{max} 2158, 1487, 1248 cm^{-1}.

3.2.2 Trimethyl((4-(trifluoromethyl)phenyl)ethynyl)silane (**2b**)

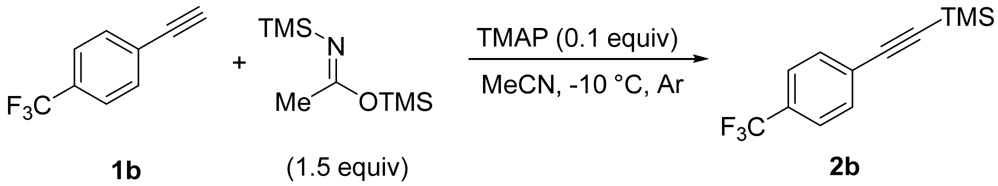

Prepared using **GP2** using 1-ethynyl-4-(trifluoromethyl)benzene (164 µL, 170 mg, 1 mmol) to give after 1 hour reaction time, 227 mg (94 %) of **2b** as a colorless oil. Spectral data corresponds to previously published data.[14]

^{1}H NMR (300 MHz, $CDCl_3$) δ 7.56 (s, 4H), 0.27 (s, 9H). **^{13}C{^{1}H} NMR** (75 MHz, $CDCl_3$) δ 132.3, 130.3 (q, *J* = 32.7 Hz), 127.1, 125.3 (q, *J* = 3.8 Hz), 124.1 (q, *J* = 272.2 Hz), 103.6, 97.4, -0.0. **IR** (neat, ATR): ν_{max} 2161, 1320, 1250 cm^{-1}.

3.2.3 ((4-Methoxyphenyl)ethynyl)trimethylsilane (**2c**)

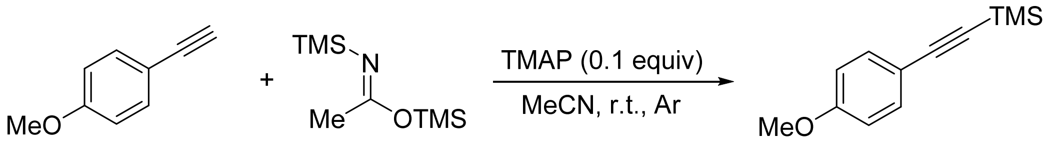

1c (1.5 equiv) **2c**

Prepared using **GP1** using 1-ethynyl-4-methoxybenzene (132 mg, 1 mmol) to give after 5 h reaction time, 174 mg (85 %) of **2c** as a colorless oil. Spectral data corresponds to previously published data.[14]

^{1}H NMR (300 MHz, $CDCl_3$) δ 7.53 – 7.33 (m, 2H), 6.93 – 6.73 (m, 2H), 3.81 (s, 3H), 0.24 (s, 9H). **^{13}C{^{1}H} NMR** (75 MHz, $CDCl_3$) δ 159.9, 133.6, 115.5, 114.0, 105.4, 92.6, 55.4, 0.2. **IR** (neat, ATR): ν_{max} 2154, 1463, 1245 cm^{-1}. **HRMS (ESI$^+$) m/z:** [M+H]$^+$ calculated for $C_{12}H_{17}OSi^+$ 205.1044, observed 205.1034, Δ = -4.9 ppm.

3.2.4 *N,N*-Dimethyl-4-((trimethylsilyl)ethynyl)aniline (**2d**)

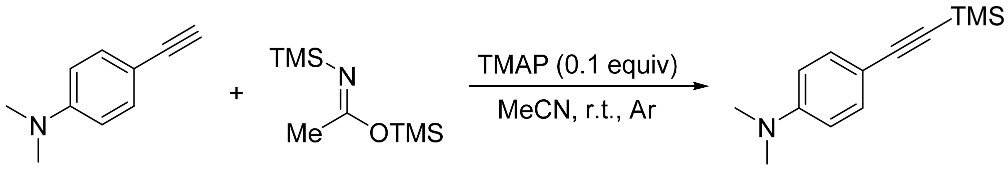

1d (1.5 equiv) **2d**

Prepared using **GP1** using 4-ethynyl-*N,N*-dimethylaniline (145 mg, 1 mmol) to give after 5 h reaction time, 200 mg (92 %) of **2d** as a beige solid. Spectral data corresponds to previously published data.[14]

^{1}H NMR (300 MHz, $CDCl_3$) δ 7.46 – 7.28 (m, 2H), 6.76 – 6.37 (m, 2H), 2.97 (s, 6H), 0.23 (s, 9H). **^{13}C{^{1}H} NMR** (75 MHz, $CDCl_3$) δ 150.4, 133.3, 111.8, 110.1, 106.7, 91.3, 40.3, 0.4. **IR** (neat, ATR): ν_{max} 2139, 1442, 1244 cm^{-1}. **HRMS (ESI$^+$) m/z:** [M+H]$^+$ calculated for $C_{13}H_{20}NSi^+$ 218.1360, observed 218.1358, Δ = -0.9 ppm. **mp** 89 – 91 °C.

3.2.5 Trimethyl(*p*-tolylethynyl)silane (**2e**)

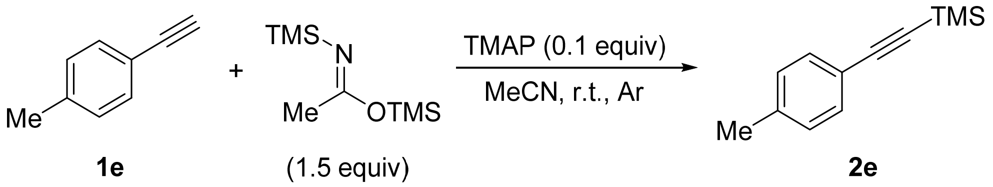

Prepared using **GP1** using 1-ethynyl-4-methylbenzene (127 µL, 116 mg, 1 mmol) to give after 6 h reaction time, 166 mg (86 %) of **2e** as an off-white solid. Spectral data corresponds to previously published data.[14]

^{1}H NMR (300 MHz, $CDCl_3$) δ 7.44 – 7.31 (m, 2H), 7.19 – 7.01 (m, 2H), 2.34 (s, 3H), 0.25 (s, 9H). **^{13}C{^{1}H} NMR** (75 MHz, $CDCl_3$) δ 138.7, 132.0, 129.1, 120.3, 105.5, 93.4, 21.6, 0.2. **IR** (neat, ATR): ν_{max} 2156, 1505, 1247 cm^{-1}. **mp** 34 – 35 °C.

3.2.6 ((4-Fluorophenyl)ethynyl)trimethylsilane (**2f**)

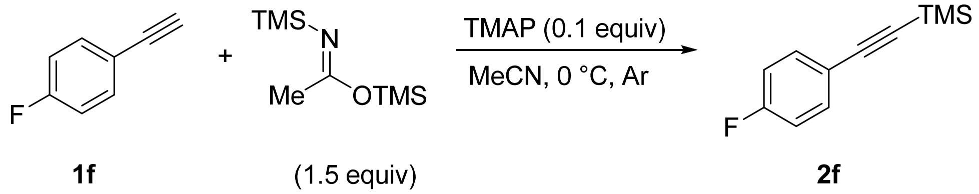

Prepared using **GP2** using 1-ethynyl-4-fluorobenzene (115 µL, 120 mg, 1 mmol) to give after 3 h reaction time, 167 mg (87 %) of **2f** as a colorless oil. Spectral data corresponds to previously published data.[15]

^{1}H NMR (300 MHz, $CDCl_3$) δ 7.55 – 7.32 (m, 2H), 7.07 – 6.83 (m, 2H), 0.25 (s, 9H). **^{13}C{^{1}H} NMR** (75 MHz, $CDCl_3$) δ 162.8 (d, $J_{C,F}$ = 249.7 Hz), 134.1 (d, $J_{C,F}$ = 8.3 Hz), 119.5 (d, $J_{C,F}$ = 3.5 Hz), 115.6 (d, $J_{C,F}$ = 22.0 Hz), 104.1, 94.0 (d, $J_{C,F}$ = 1.4 Hz), 0.1. **IR** (neat, ATR): ν_{max} 2160, 1503, 1251 cm^{-1}.

3.2.7 ((4-Chlorophenyl)ethynyl)trimethylsilane (**2g**)

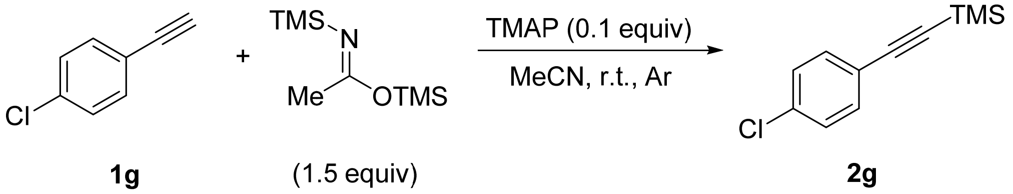

Prepared using **GP1** using 1-chloro-4-ethynylbenzene (68 mg, 0.5 mmol) to give after 5 h reaction time, 95 mg (91 %) of **2g** as a white solid. Spectral data corresponds to previously published data.[14]

^{1}H NMR (300 MHz, $CDCl_3$) δ 7.51 – 7.32 (m, 2H), 7.30 – 7.17 (m, 2H), 0.23 (s, 9H). **^{13}C{^{1}H} NMR** (75 MHz, $CDCl_3$) δ 134.7, 133.3, 128.7, 121.8, 104.0, 95.5, 0.0(4). **IR** (neat, ATR): ν_{max} 2156, 1484, 1258 cm^{-1}. **mp** 52 –54 °C.

3.2.8 ((2-Chlorophenyl)ethynyl)trimethylsilane (2h)

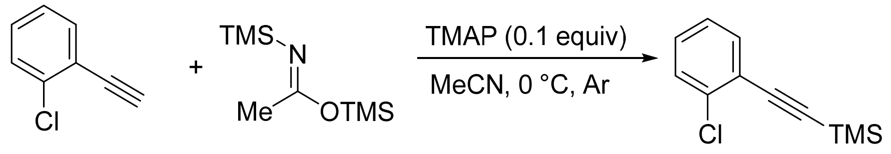

1h (1.5 equiv) **2h**

Prepared using **GP2** using 1-chloro-2-ethynylbenzene (124 µL, 136 mg, 1 mmol) to give after 3 h reaction time, 190 mg (91 %) of **2h** as a clear yellow oil. Spectral data corresponds to previously published data.[16]

^{1}H NMR (300 MHz, $CDCl_3$) δ 7.55 – 7.44 (m, 1H), 7.45 – 7.33 (m, 1H), 7.25 – 7.10 (m, 2H), 0.28 (s, 9H). **^{13}C{^{1}H} NMR** (75 MHz, $CDCl_3$) δ 136.4, 133.8, 129.6, 129.4, 126.4, 123.2, 101.4, 100.4, 0.0(2). **IR** (neat, ATR): ν_{max} 2163, 1469, 1249 cm^{-1}.

3.2.9 ((3,5-Bis(trifluoromethyl)phenyl)ethynyl)trimethylsilane (2i)

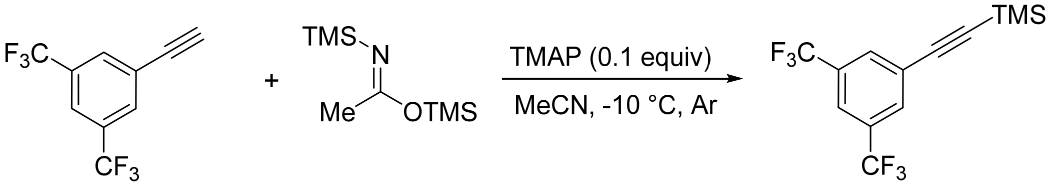

1i (1.5 equiv) **2i**

Prepared using **GP2** using 1-ethynyl-3,5-bis(trifluoromethyl)benzene (177 µL, 238 mg, 1 mmol) to give after 2 h reaction time, 284 mg (92 %) of **2i** as a white solid. Spectral data corresponds to previously published data.[17]

^{1}H NMR (300 MHz, $CDCl_3$) δ 7.88 (s, 2H), 7.79 (s, 1H), 0.28 (s, 9H). **^{13}C{^{1}H} NMR** (75 MHz, $CDCl_3$) δ 132.0 (q, $J_{C,F}$ = 33.8 Hz), 132.2 – 131.8 (m), 125.7, 123.1 (q, $J_{C,F}$ = 272.9 Hz), 121.9 (hept, $J_{C,F}$ = 3.8 Hz), 101.7, 98.9, -0.2. **IR** (neat, ATR): ν_{max} 2174, 1460, 1249 cm^{-1}. **mp** 48 – 49 °C.

3.2.10 Ferrocenylethynetrimethylsilane (2j)

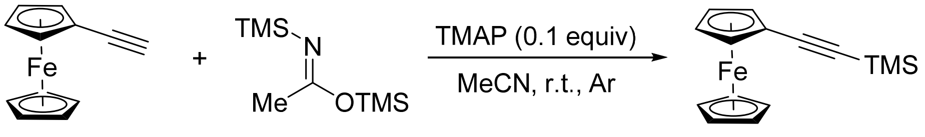

1j (1.5 equiv) **2j**

Prepared using **GP1** using ethynylferrocene (105 mg, 0.5 mmol) to give after 5 h reaction time, 130 mg (92 %) of **2j** as a red-brown solid. Spectral data corresponds to previously published data.[18]

^{1}**H NMR** (300 MHz, $CDCl_3$) δ 4.43 (apparent t, *J* = 1.8 Hz, 2H), 4.20 (s, 5H), 4.18 (apparent t, *J* = 1.9 Hz, 2H), 0.23 (s, 9H). ^{13}**C{^{1}H}** NMR (75 MHz, CDCl3) δ 104.3, 90.7, 71.9, 70.3, 68.9, 65.0, 0.4. **IR** (neat, ATR): ν_{max} 2146, 1453, 1247 cm^{-1}. **mp** 55 – 56 °C.

3.2.11 3-((Trimethylsilyl)ethynyl)pyridine (**2k**)

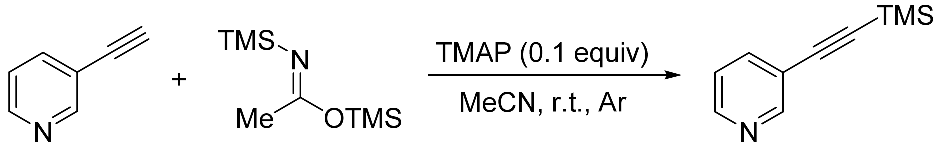

1k (1.5 equiv) **2k**

Prepared using **GP1** using 3-ethynylpyridine (72 µl, 52 mg, 0.5 mmol) to give after 5 h reaction time, 60 mg (69 %) of **2k** as a colorless oil. Spectral data corresponds to previously published data.[14]

^{1}**H NMR** (300 MHz, $CDCl_3$) δ 8.69 (dd, *J* = 2.2, 0.9 Hz, 1H), 8.52 (dd, *J* = 4.9, 1.7 Hz, 1H), 7.73 (dt, *J* = 7.9, 1.9 Hz, 1H), 7.22 (ddd, *J* = 7.9, 4.9, 0.9 Hz, 1H), 0.26 (s, 9H). ^{13}**C{^{1}H} NMR** (75 MHz, $CDCl_3$) δ 152.8, 148.9, 138.9, 123.0, 120.4, 101.6, 98.4, -0.1. **IR** (neat, ATR): ν_{max} 2162, 1474, 1249 cm^{-1}.

3.2.12 Trimethyl(thiophen-3-ylethynyl)silane (**2l**)

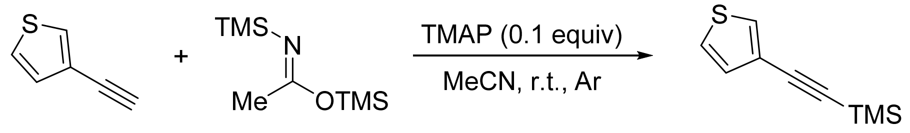

1l (3 equiv) **2l**

Prepared using **GP1** 3-ethynylthiophene (60 mg, 0.50 mmol) and BSA (367 µL, 305 mg, 1.50 mmol, 3 equiv) to give after 3 h reaction time, 88 mg (98 %) of **2l** as a colorless oil. Spectral data corresponds to previously published data.[14]

^{1}**H NMR** (300 MHz, $CDCl_3$) δ 7.48 (dd, *J* = 3.0, 1.1 Hz, 1H), 7.23 (ddd, *J* = 5.0, 3.0, 0.6 Hz, 1H), 7.12 (dd, *J* = 5.0, 1.2 Hz, 1H), 0.24 (s, 4H). ^{13}**C{^{1}H} NMR** (75 MHz, $CDCl_3$) δ 130.3, 129.7, 125.3, 122.5, 100.0, 94.0, 0.1. **IR** (neat, ATR): ν_{max} 2153, 1356, 1248 cm^{-1}.

3.2.13 ((6-Methoxynaphthalen-2-yl)ethynyl)trimethylsilane (**2m**)

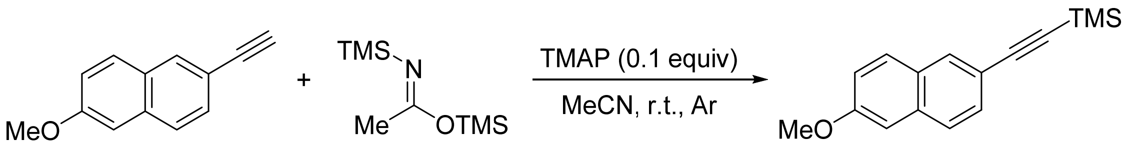

1m (3 equiv) **2m**

Prepared using **GP1** 2-ethynyl-6-methoxynaphthalene (91 mg, 0.50 mmol) and BSA (367 µL, 305 mg, 1.50 mmol, 3 equiv) to give after 5 h reaction time, 110 mg (87 %) of **2m** as a white solid. Spectral data corresponds to previously published data.[19]

^{1}H NMR (300 MHz, $CDCl_3$) δ 7.92 (s, 1H), 7.70−7.61 (m, 2H), 7.47 (dd, *J* = 8.4, 1.6 Hz, 1H), 7.14 (dd, *J* = 8.9, 2.5 Hz, 1H), 7.09 (d, *J* = 2.5 Hz, 1H), 3.92 (s, 3H), 0.28 (s, 9H). **^{13}C{^{1}H} NMR** (75 MHz, $CDCl_3$) δ 158.6, 134.4, 132.0, 129.5, 129.4, 128.5, 126.8, 119.5, 118.2, 106.0, 105.9, 93.8, 55.5, 0.2. **IR** (neat, ATR): ν_{max} 2152, 1478, 1233 cm^{-1}. **mp** 104 – 105 °C.

3.2.14 *tert*-Butyldimethyl((3-(trimethylsilyl)prop-2-yn-1-yl)oxy)silane (2n)

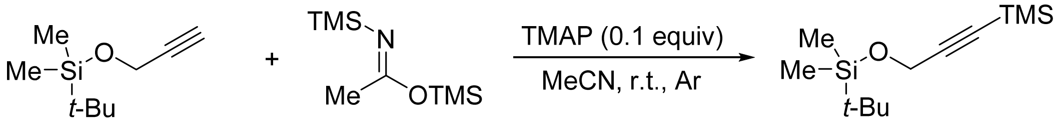

1n (1.5 equiv) **2n**

Prepared using **GP1** using *tert*-butyldimethyl(prop-2-yn-1-yloxy)silane (170 mg, 1 mmol) to give after 5 h reaction time, 210 mg (87 %) of **2n** as an colourless oil. Spectral data corresponds to previously published data.[14]

^{1}H NMR (300 MHz, $CDCl_3$) δ 4.30 (s, 2H), 0.91 (s, 9H), 0.16 (s, 9H), 0.12 (s, 6H). **^{13}C{^{1}H}** NMR (75 MHz, $CDCl_3$) δ 104.7, 89.8, 52.4, 26.0, 18.4, -0.1, -4.9. **IR** (neat, ATR): ν_{max} 2153, 1474, 1249 cm^{-1}. **HRMS (ESI$^+$) m/z:** [M+Na]$^+$ calculated for $C_{12}H_{26}OSi_2Na^+$ 265.1415, observed 265.1408, Δ = -2.6 ppm.

3.2.15 Trimethyl(3-(oxiran-2-ylmethoxy)prop-1-yn-1-yl)silane (2o)

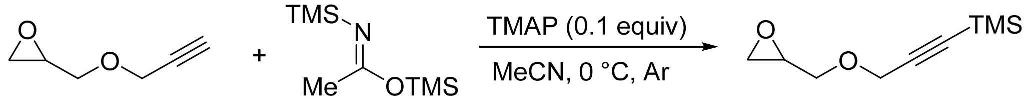

1o (1.5 equiv) **2o**

Prepared using **GP2** using 2-((prop-2-yn-1-yloxy)methyl)oxirane (108 µL, 112 mg, 1 mmol) to give after 2 h reaction time, 179 mg (97 %) of **2o** as a colorless oil.

^{1}H NMR (300 MHz, $CDCl_3$) δ 4.19 (obs. ABq, 2H, Δv = 12 Hz, $|J_{AB}|$ = 16.0 Hz, 2H), 3.78 (dd, *J* = 11.3, 3.3 Hz, 1H), 3.49 (dd, *J* = 11.3, 5.7 Hz, 1H), 3.20 – 3.13 (m, 1H), 2.82 – 2.78 (m, 1H), 2.63 (dd, *J* = 5.1, 2.7 Hz, 1H), 0.18 (s, 9H). **^{13}C{^{1}H} NMR** (75 MHz, $CDCl_3$) δ 101.1, 92.0, 70.5, 59.4, 50.6, 44.6, -0.1. **IR** (neat, ATR): ν_{max} 2175, 1441, 1249 cm^{-1}. **HRMS (ESI$^+$) m/z:** [M+Na]$^+$ calculated for $C_9H_{16}O_2SiNa^+$ 207.0812, observed 207.0818, Δ = 2.9 ppm.

Another batch of **2o** was prepared in larger scale using 785 mg (7.0 mmol) of 2-((prop-2-yn-1-yloxy)methyl)oxirane, 2.57 mL (10.5 mmol) of BSA and TMAP (124 mg, 700.0 µmol) after 2 h to afford **2o** (1.18 g, 91%) as a colorless oil. ^{1}H NMR spectrum of the resulting **2o** fully matched the data obtained in the small-scale batch.

3.2.16 1-(3-(Trimethylsilyl)prop-2-yn-1-yl)-*1H*-indole (**2p**)

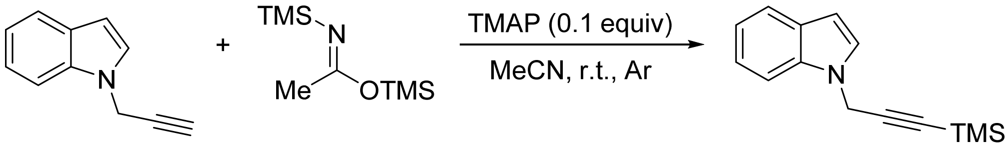

1p (1.5 equiv) **2p**

Prepared using **GP1** 1-(prop-2-yn-1-yl)-1H-indole (60 mg, 0.50 mmol) and to give after 5 h reaction time, 90 mg (75 %) of **2p** as a beige solid. Spectral data corresponds to previously published data.[20]

^{1}H NMR (300 MHz, $CDCl_3$) δ 7.66 (dt, *J* = 7.8, 1.0 Hz, 1H), 7.42 (dd, *J* = 8.3, 0.9 Hz, 1H), 7.31 – 7.22 (m, 2H), 7.15 (ddd, *J* = 8.1, 7.1, 1.1 Hz, 1H), 6.55 (dd, *J* = 3.2, 0.9 Hz, 2H), 4.90 (s, 2H), 0.20 (s, 9H). **^{13}C{^{1}H} NMR** (75 MHz, $CDCl_3$) δ 136.0, 129.0, 127.3, 121.9, 121.2, 119.9, 109.5, 101.9, 99.3, 90.8, 36.9, -0.1. **IR** (neat, ATR): ν_{max} 2177, 1462, 1247 cm^{-1}. **mp** 57 −59°C. **HRMS (ESI$^+$) m/z:** [M+H]$^+$ calculated for $C_{14}H_{18}NSi^+$ 228.1204, observed 228.1203. Δ = − 0.4 ppm.

3.2.17 (Cyclohex-1-en-1-ylethynyl)trimethylsilane (**2q**)

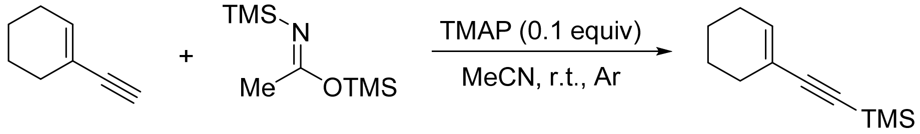

1q (1.5 equiv) **2q**

Prepared using **GP1** using 1-ethynylcyclohex-1-ene (118 µL, 106 mg, 1 mmol) to give after 3 h reaction time, 126 mg (71 %) of **2q** as a clear yellow oil. Spectral data corresponds to previously published data.[14]

^{1}H NMR (500 MHz, $CDCl_3$) δ 6.18 (tt, *J* = 4.1, 1.8 Hz, 1H), 2.16 – 2.03 (m, 4H), 1.67 – 1.53 (m, 4H), 0.18 (s, 9H). **^{13}C{^{1}H} NMR** (126 MHz, $CDCl_3$) δ 136.4, 120.9, 107.5, 91.0, 29.2, 25.8, 22.4, 21.6, 0.3. **IR** (neat, ATR): ν_{max} 2146, 1448, 1248 cm^{-1}.

3.2.18 Hex-1-yn-1-yltrimethylsilane (**2r**)

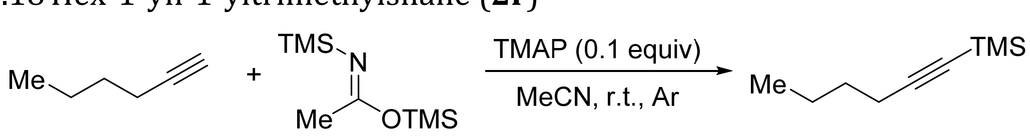

1r (1.5 equiv) **2r**

Prepared using **GP1** using hexyne (230 µl, 164 mg, 2 mmol) to give after 3 days reaction time, 190 mg (62 %) of **2r** as a colorless oil. Spectral data corresponds to previously published data.[21]

^{1}H NMR (300 MHz, $CDCl_3$) δ 2.22 (t, *J* = 6.9 Hz, 2H), 1.65 – 1.30 (m, 4H), 0.91 (t, *J* = 7.2 Hz, 3H), 0.14 (s, 9H). **^{13}C{^{1}H} NMR** (75 MHz, $CDCl_3$) δ 107.9, 84.4, 30.9, 22.1, 19.7, 13.7, 0.3. **IR** (neat, ATR): ν_{max} 2174, 1465, 1249 cm^{-1}.

3.2.19 Trimethyl(4-phenylbut-1-yn-1-yl)silane (2s)

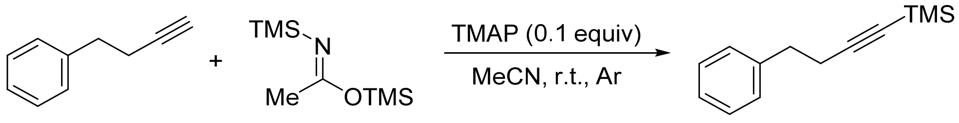

1s (3 equiv) **2s**

Prepared using **GP1** using but-3-yn-1-ylbenzene (144 µL, 133 mg, 1 mmol) and BSA (733 µL, 610 mg, 3 mmol, 3 equiv) to give after 48 h reaction time and column purification (pentane:Et_2O – 100:0 to 95:5), 115 mg (55 %, 96% purity of sample) of **2s** as a colorless oil. Spectral data corresponds to previously published data.[22]

^{1}H NMR (300 MHz, $CDCl_3$) δ 7.37 – 7.18 (m, 5H), 2.87 (t, *J* = 7.6 Hz, 2H), 2.53 (t, *J* = 7.6 Hz, 2H), 0.18 (s, 9H). **^{13}C{^{1}H} NMR** (75 MHz, $CDCl_3$) δ 140.8, 128.6, 128.4, 126.4, 106.8, 35.3, 22.3, 0.2. **IR** (neat, ATR): ν_{max} 2174, 1454, 1248 cm^{-1}. **HRMS (ESI$^+$) m/z:** [M+Na]$^+$ calculated for $C_{13}H_{18}SiNa^+$ 225.1070, observed 225.1070. Δ = 0 ppm.

3.2.20 6-(Trimethylsilyl)hex-5-ynenitrile (2t)

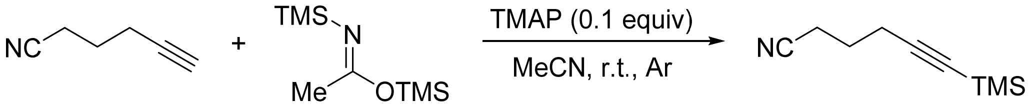

1t (3 equiv) **2t**

Prepared using **GP1** using hex-5-ynenitrile (107 µL, 93 mg, 1 mmol) and BSA (733 µL, 610 mg, 3 mmol, 3 equiv) to give after 48 h reaction time, 125 mg (70 %) of **2t** as a colorless oil. Spectral data corresponds to previously published data.[23]

^{1}H NMR (300 MHz, $CDCl_3$) δ 2.48 (t, *J* = 7.2 Hz, 2H), 2.40 (t, *J* = 6.7 Hz, 2H), 1.86 (p, *J* = 7.0 Hz, 2H), 0.15 (s, 9H). **^{13}C{^{1}H} NMR** (75 MHz, $CDCl_3$) δ 119.3, 104.0, 87.0, 24.6, 19.1, 16.2, 0.1. **IR** (neat, ATR): ν_{max} 2176, 1431, 1250 cm^{-1}. **HRMS (ESI$^+$) m/z:** [M+Na]$^+$ calculated for $C_9H_{15}NSiNa^+$ 188.0866, observed 188.0868. Δ = 1.1 ppm.

3.2.21 1,3-Bis((trimethylsilyl)ethynyl)benzene (2u)

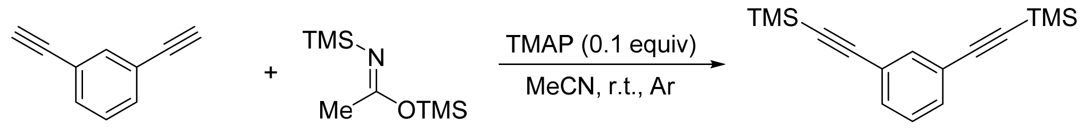

1u (3 equiv) **2u**

Prepared using **GP1** using 1,3-diethynylbenzene (139 µL, 131 mg, 1 mmol) and BSA (734 µL, 610 mg, 3 mmol, 3 equiv) to give after 3 h reaction time, 269 mg (99 %) of **2u** as a beige solid. Spectral data corresponds to previously published data.[14]

^{1}H NMR (300 MHz, $CDCl_3$) δ 7.58 (dt, *J* = 1.7, 0.8 Hz, 1H), 7.39 (ddd, *J* = 7.5, 1.6, 0.6 Hz, 2H), 7.22 (ddd, *J* = 8.4, 7.1, 0.6 Hz, 1H), 0.24 (s, 18H). **^{13}C{^{1}H} NMR** (75 MHz, $CDCl_3$) δ 135.6,

131.9, 128.3, 123.5, 104.2, 95.0, 0.1. **IR** (neat, ATR): ν_{max} 2153, 1474, 1249 cm^{-1}. **mp** 59 – 61 ⍰.

3.2.22 *(((8R,9S,13S,14S,17S)*-13-Methyl-17-((trimethylsilyl)ethynyl)-7,8,9,11,12,13,14,15,16,17-decahydro-*6H*-cyclopenta[*a*]phenanthrene-3,17-diyl)bis(oxy))bis(trimethylsilane) (**2v**)

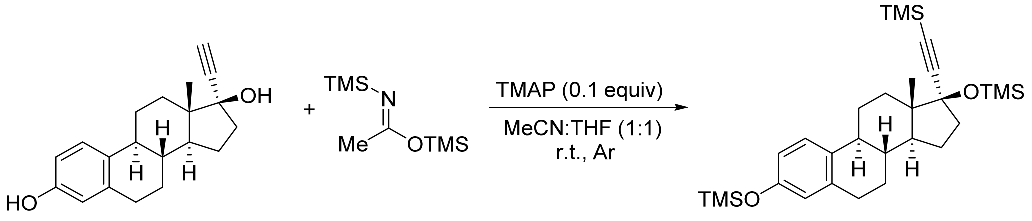

1v (7 equiv) **2v**

Prepared using **GP1.** A mixture of,MeCN:THF (v/v 1:1) was used as the solvent., Ethynylestradiol (148 mg, 0.50 mmol) and BSA (856 µL, 712 mg, 3.50 mmol, 7 equiv) were reacted to give, after 24 h of reaction time, 240 mg of **2v** of 90% purity (corresponding to 216 mg (84%) of **2v**) as a white solid. Based on the small ^{1}H NMR signal at 2.6 ppm (br s), the impurity is likely to be the bis-*O*-silylated **1v**. In the literature, **2v** has been obtained in situ and used further without isolation and purification.[24]

^{1}H NMR (300 MHz, $CDCl_3$) δ 7.16 (d, *J* = 8.2 Hz, 1H), 6.63 (dd, *J* = 8.4, 2.7 Hz, 1H), 6.56 (d, *J* = 2.6 Hz, 1H), 2.85 – 2.74 (m, 2H), 2.44 – 2.01 (m, 3H), 2.02 – 1.58 (m, 6H), 1.56 – 1.21 (m, 4H), 0.81 (s, 3H), 0.26 (s, 9H), 0.18 (s, 18H). **^{13}C{^{1}H} NMR** (75 MHz, $CDCl_3$) δ 153.0, 138.1, 133.6, 126.4, 120.1, 117.4, 110.5, 90.8, 81.1, 48.7, 48.1, 44.1, 40.7, 39.7, 33.0, 29.9, 27.6, 26.7, 23.2, 13.1, 2.1, 0.4, 0.1. **IR** (neat, ATR): ν_{max} 2162, 1497, 1245, 1091 cm^{-1}. **HRMS (ESI^{+}) m/z:** [M+Na]$^{+}$ calculated for $C_{29}H_{48}O_2Si_3Na^{+}$ 535.2855, observed 535.2856. Δ = 0.2 ppm. **mp** 114 – 116 °C. $[\alpha]_D^{20}$ = –18 (*c* = 0.1, DCM).

3.2.23 *tert*-Butyl (trimethylsilyl)(3-(trimethylsilyl)prop-2-yn-1-yl)carbamate (**2w**)

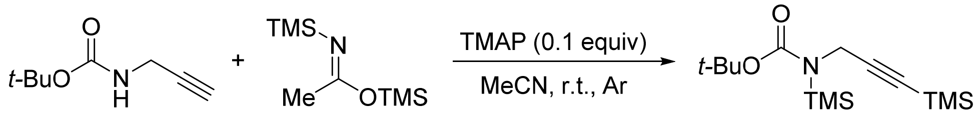

1w (5 equiv) **2w**

Prepared using **GP1** *tert*-butyl prop-2-yn-1-ylcarbamate (78 mg, 0.50 mmol) and BSA (611 µL, 506 mg, 2.50 mmol, 5 equiv) to give after 5 h reaction time, 142 mg (95 %) of **2w** as a colorless oil.

^{1}H NMR (300 MHz, $CDCl_3$) δ 3.90 (s, 2H), 1.46 (s, 9H), 0.27 (s, 9H), 0.11 (s, 9H). **^{13}C{^{1}H} NMR** (75 MHz, $CDCl_3$) δ 157.7, 104.7, 86.8, 80.5, 34.5, 28.5, 0.6, -0.0(4). **IR** (neat, ATR): ν_{max} 2176, 1476, 1318, 1248, 1165, 838 cm^{-1}. **HRMS (ESI^{+}) m/z:** [M+K]$^{+}$ calculated for $C_{14}H_{29}NO_2Si_2K^{+}$ 338.1369, observed 338.1381. Δ = 3.5 ppm.

3.2.24 *tert*-Butyl (3-(trimethylsilyl)prop-2-yn-1-yl)carbamate (**2x**)

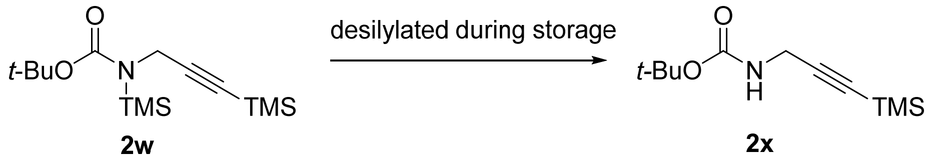

2x was obtained spontaneously during crystallization of **2w** in the fridge as a colorless crystals (107 mg, 99% mass balance), analyzed without further purification.

^{1}H NMR (300 MHz, $CDCl_3$) δ 4.64 (s, 1H), 3.93 (d, *J* = 5.4 Hz, 2H), 1.45 (s, 9H), 0.15 (s, 9H). **^{13}C{^{1}H} NMR** (75 MHz, $CDCl_3$) δ 155.3, 102.0, 88.1, 80.1, 31.6, 28.5, -0.1. **IR** (neat, ATR): ν_{max} 3345, 2179, 1247 cm^{-1}. **HRMS (ESI$^+$) m/z:** $[M+Na]^+$ calculated for $C_{11}H_{21}NO_2SiNa^+$ 250.1234, observed 250.1234. Δ = 0 ppm. **mp** 61 – 64 °C.

3.2.25 *tert*-Butyldimethyl(phenylethynyl)silane (**4**)

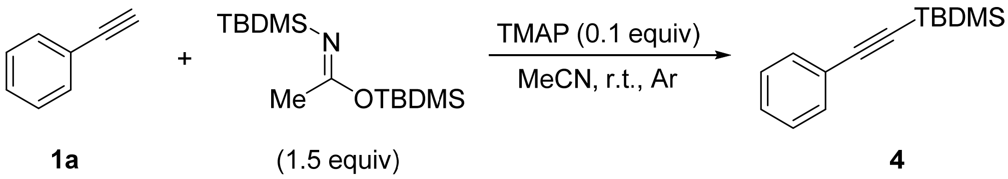

Prepared using **GP1** using phenylacetylene (55 µL, 51 mg, 0.50 mmol) and BTBSA (216 mg, 0.75 mmol, 1.5 equiv) to give, after 5 h reaction time, a crude product as an oil which was dried in high vacuum (traces of TBDMSOH might have remained after filtration through the silica pad) to a constant weight of 70 mg (65 %) of **4** as a colorless oil. Spectral data corresponds to previously published data.[25]

^{1}H NMR (300 MHz, $CDCl_3$) δ 7.61 – 7.40 (m, 2H), 7.34 – 7.27 (m, 3H), 1.01 (s, 9H), 0.20 (s, 6H).**^{13}C{^{1}H} NMR** (75 MHz, $CDCl_3$) δ 132.1, 128.6, 128.3, 123.5, 105.9, 92.6, 26.3, 16.9, -4.4. **IR** (neat, ATR): ν_{max} 2158, 1488, 1249, 832, 774, 754 cm^{-1}.

3.3 Mechanistic studies

3.3.1 Kinetic studies

Preliminary experiments revealed that 1 mol% (0.01 equiv) of catalyst was sufficient to obtain measurable rates in the reaction. The use of higher catalyst loadings led to reaction rates that were generally too rapid to measure by in situ NMR technique used here (500 MHz, 30 □ probe temperature). Due to the hygroscopic nature of the catalyst and very fast rates, the reaction progress plots of two independent measurements do not always overlay. Nevertheless, we were able to obtain a reasonable Hammett relationship using averaged data of the independent rate measurements.

Procedure: Alkyne (1.0 equiv, 60 µmol), dibenzyl ether (1.0 equiv, 60 µmol), *N,O*-bis(trimethylsilyl)acetamide (1.5 equiv., 90 µmol), were dissolved in dry CD_3CN (600 µL) in NMR tube and the first ^1H NMR spectrum was recorded. The catalyst (TMAP solution, 62 mM in CD_3CN, 0.01 equiv) was then added, the NMR tube was capped and inverted three times to ensure homogeneity, and the sample was reinserted into the NMR probe. The reaction was followed by ^1H NMR for 60-70 minutes (60 scans with 1 min gap) by monitoring the diagnostic TMS-alkyne ^1H NMR peaks at 0.25-0.30 ppm. The results were processed with MestReNova kinetic plugin and converted into concentrations using integration of internal standard with the correction on the initial concentration in Microsoft Excel, and the relative rates were obtained by exponential fits using OriginPro 2017.

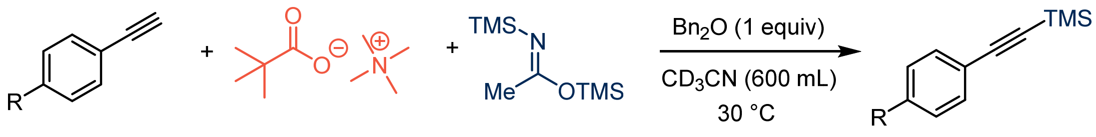

1 (60.00 µmol) **TMAP** (1 mol%) **BSA** (1.5 equiv) **2**

Scheme S1. General equation of the reaction used for ^1H NMR studies.

3.3.1.1 ^1H NMR experiment for **2a**.

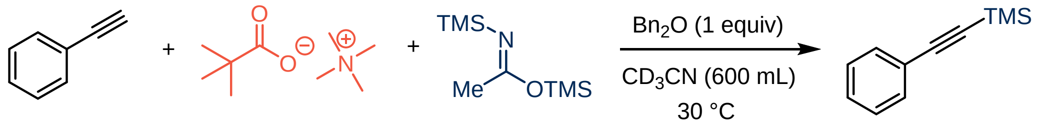

1a (60.00 µmol) **TMAP** (1 mol%) **BSA** (1.5 equiv) **2a**

1. First ^1H NMR scan of the reaction mixture 9 min after the addition of TMAP addition (which marks the start of the reaction). Zoomed area: Ph-CC-Si(CH_3)$_3$ signals.

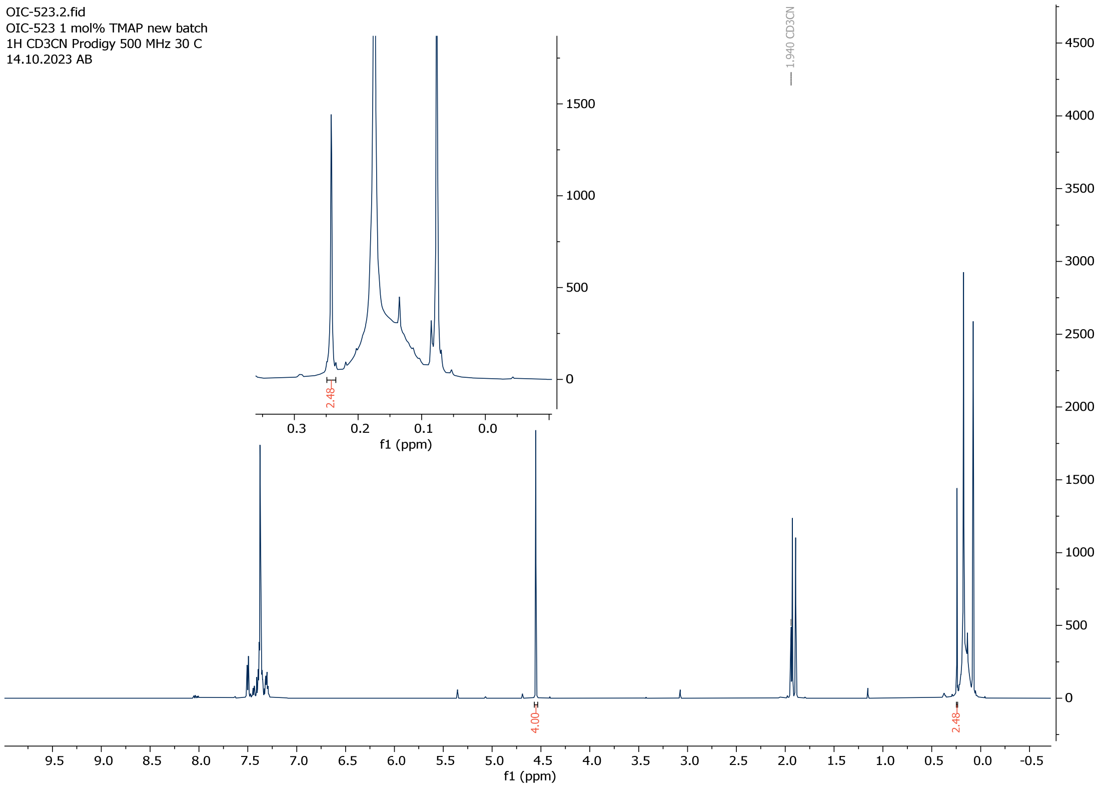

2. Last ^{1}H NMR scan of the reaction mixture, 68 min after TMAP addition (zoomed area – Ph-CC-Si(C***H***$_3$)$_3$ signals).

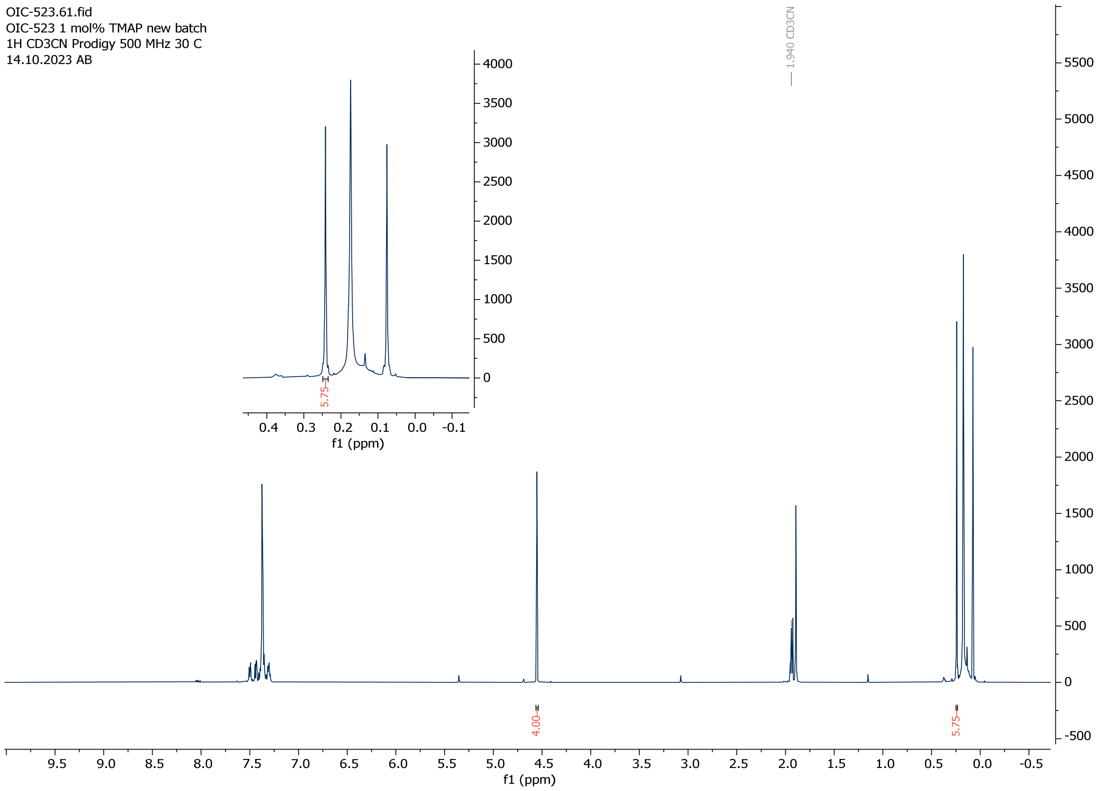

3. Experimental reaction profiles of two independent mesurments and exponential fits for **2a** formation. The relative rates can be obtained from the exponentials (k_H = 0.0494± 0.002 mM min^{-1}).

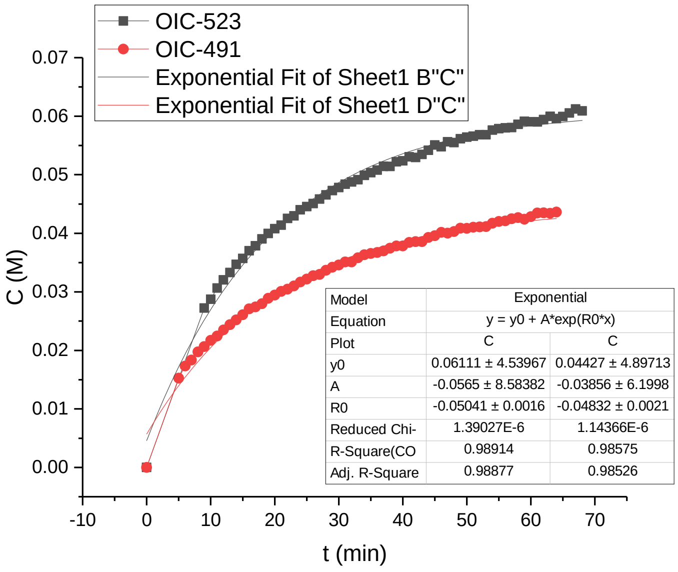

3.3.1.2 ^{1}H NMR experiment for **2g**

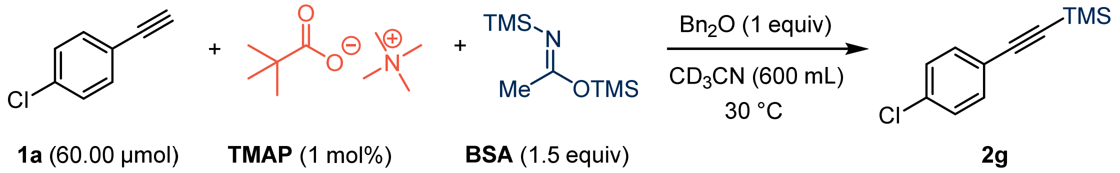

1. First ^{1}H NMR scan of the reaction mixture, 9 min after TMAP addition (zoomed area – Ar-CC-Si(C***H***$_3$)$_3$ signals).

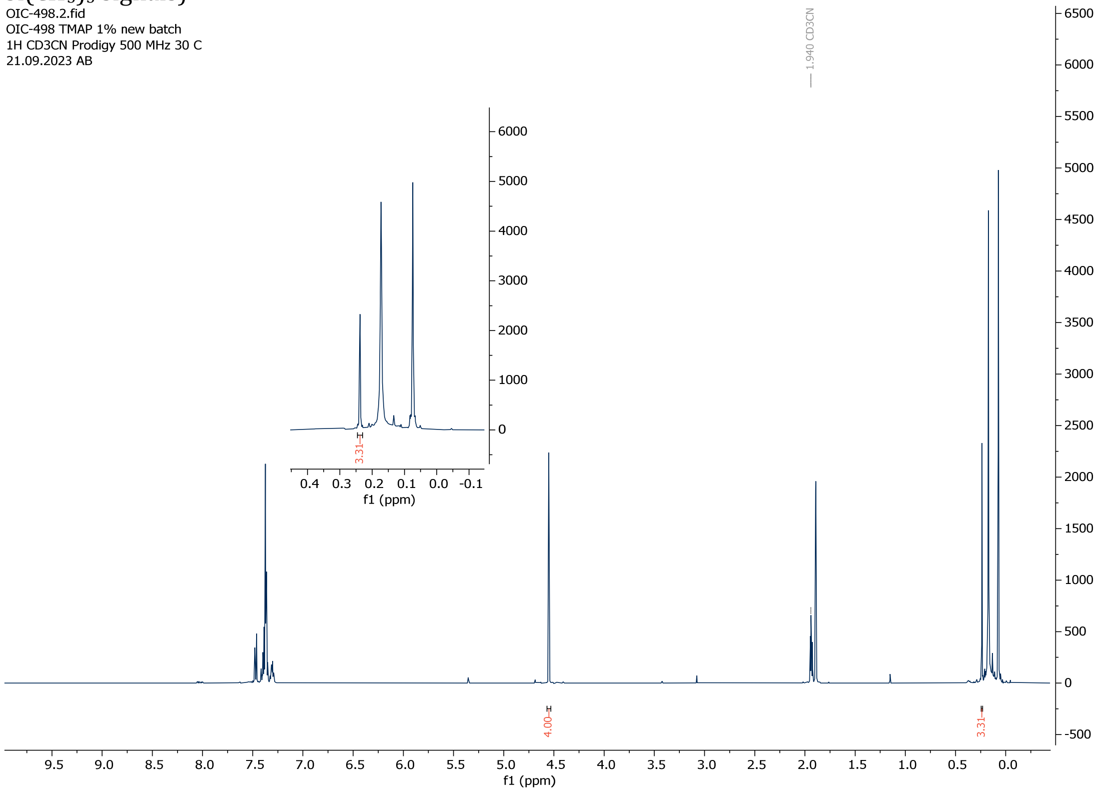

2. Last ^{1}H NMR scan of the reaction mixture, 68 min after TMAP addition (zoomed area – Ar - CC-Si(C***H***$_3$)$_3$ signals).

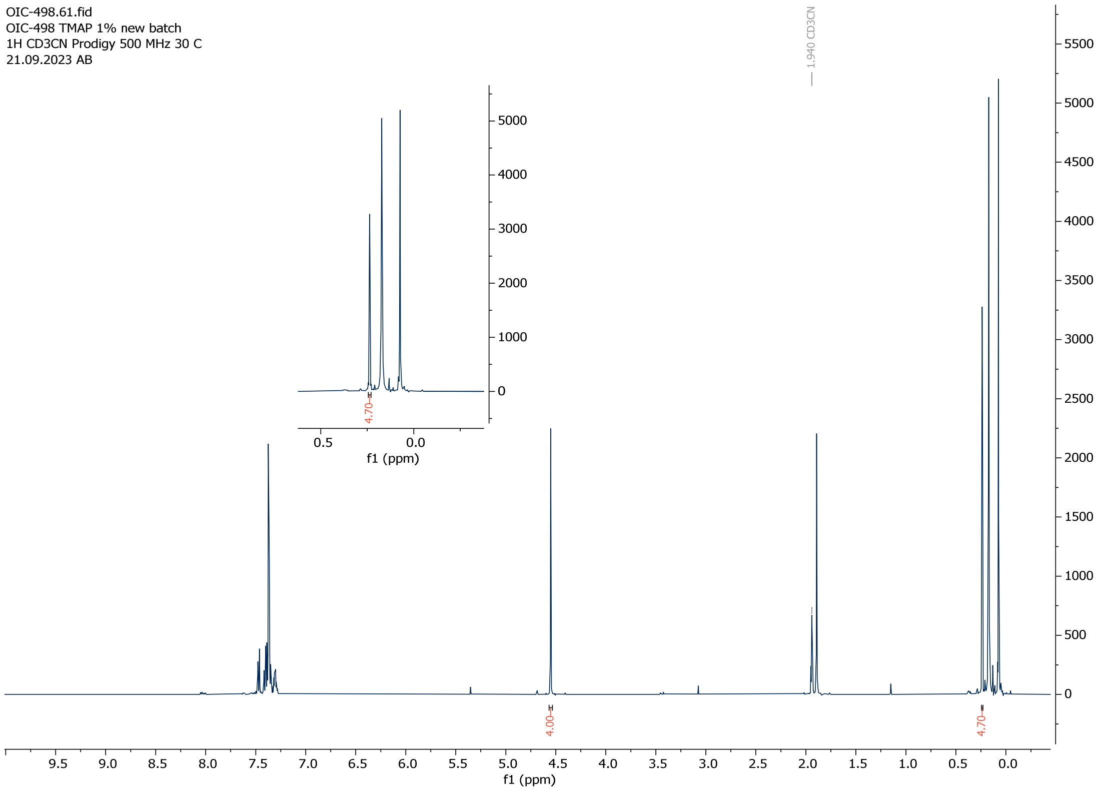

3. Experimental reaction profiles and exponential fits for **2g** formation. The relative rates can be obtained from the exponentials (k_{Cl} = 0.1325± 0.0027 mM min^{-1}).

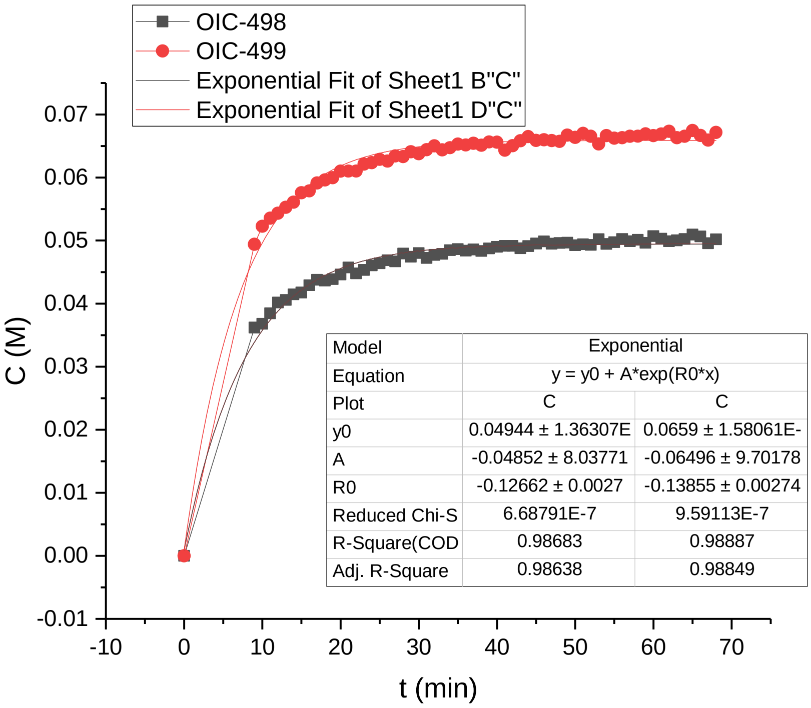

3.3.1.3 ^{1}H NMR experiment for **2h**.

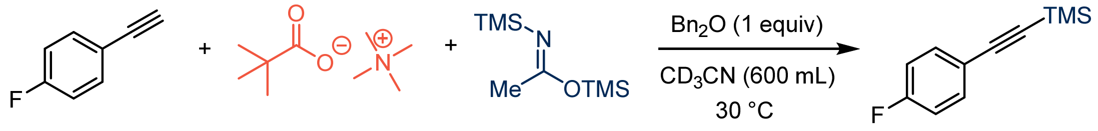

1h (60.00 μmol) **TMAP** (1 mol%) **BSA** (1.5 equiv) **2h**

1. First ^{1}H NMR scan of the reaction mixture, 9 min after TMAP addition (zoomed area – Ar - CC-Si(C***H***$_3$)$_3$ signals).

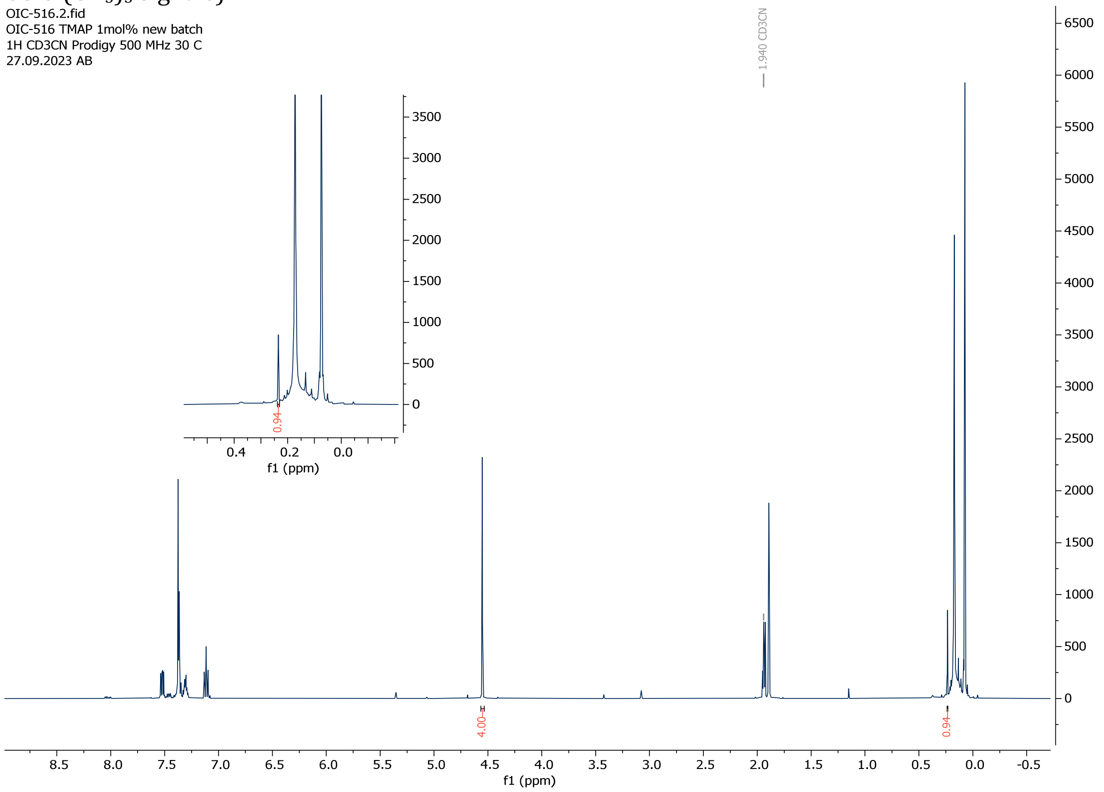

2. Last ^{1}H NMR scan of the reaction mixture, 68 min after TMAP addition (zoomed area – Ar - CC-Si(C***H***$_3$)$_3$ signals).

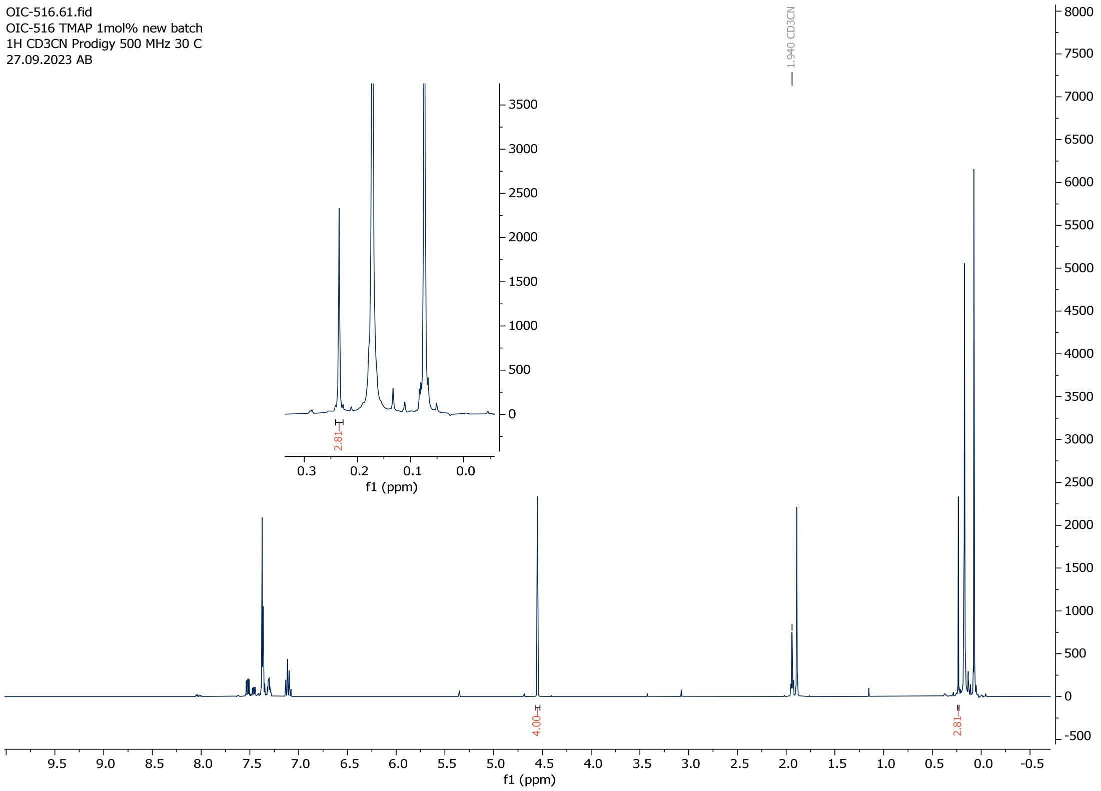

3. Experimental reaction profiles and exponential fits for **2h** formation. The relative rates can be obtained from the exponentials (k_F = 0.0523 ± 0.0018 mM min-1).

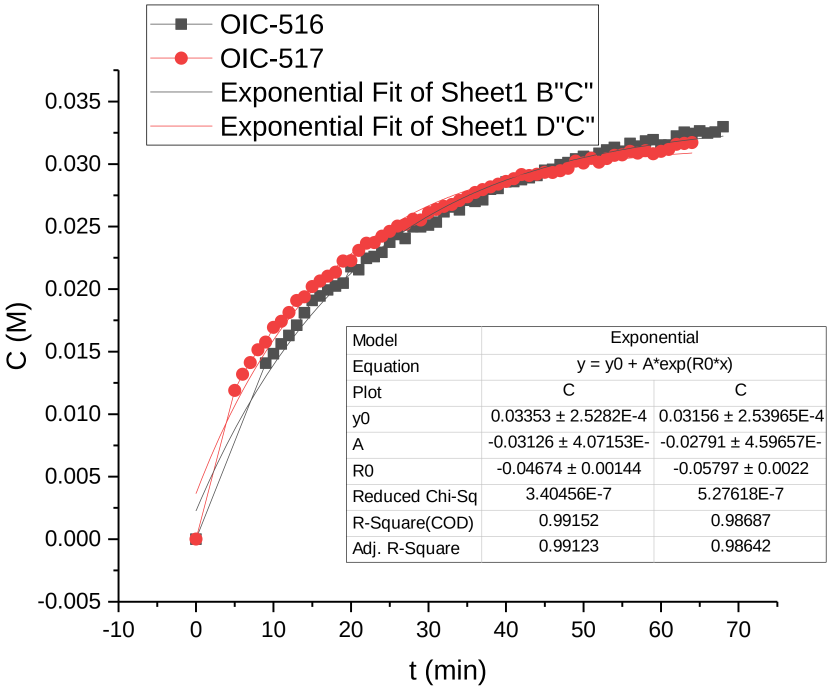

3.3.1.4 ^{1}H NMR experiment for **2c**

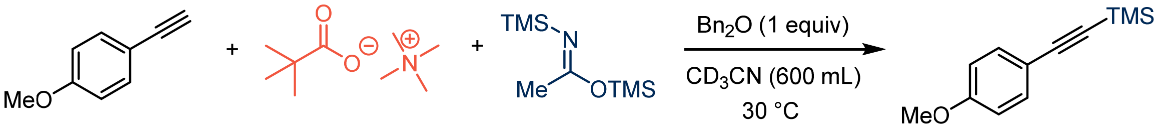

1c (60.00 µmol) **TMAP** (1 mol%) **BSA** (1.5 equiv) **2c**

1. First ^{1}H NMR scan of the reaction mixture, 8 min after TMAP addition (zoomed area – Ar - CC-Si(C***H***$_3$)$_3$ signals).

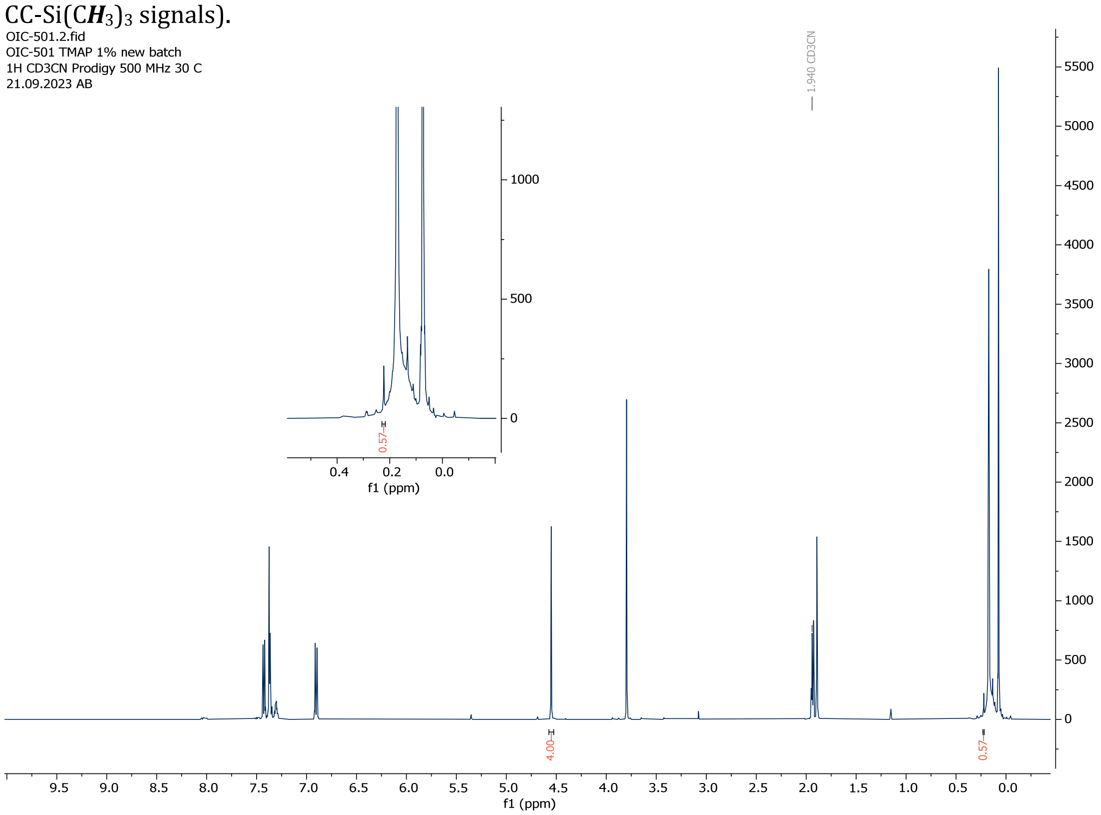

2. Last ^{1}H NMR scan of the reaction mixture, 67 min after TMAP addition (zoomed area – Ar - CC-Si(C***H***$_3$)$_3$ signals).

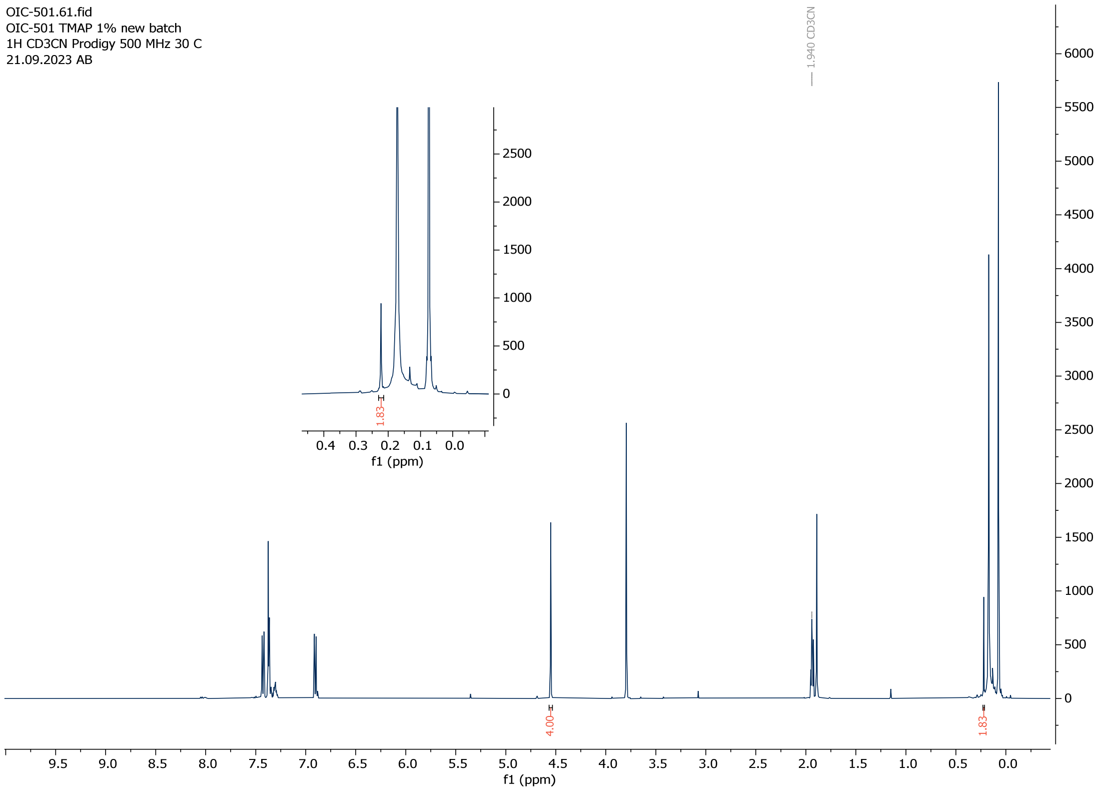

3. Experimental reaction profiles and exponential fits for **2c** formation. The relative rates can be obtained from the exponentials (k_{OMe} = 0.0254± 0.0029 mM min-1).

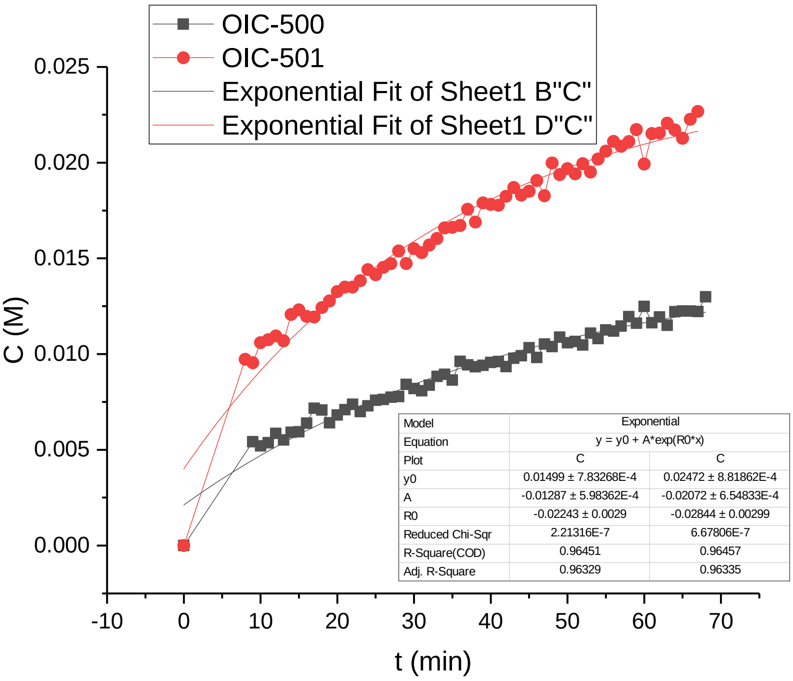

3.3.1.5 ^{1}H NMR experiment for **2e**

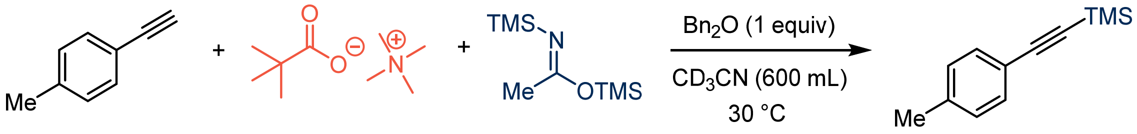

1e (60.00 μmol) **TMAP** (1 mol%) **BSA** (1.5 equiv) **2e**

1. First ^{1}H NMR scan of the reaction mixture, 9 min after TMAP addition (zoomed area – Ar-CC-Si(C***H***$_3$)$_3$ signals).

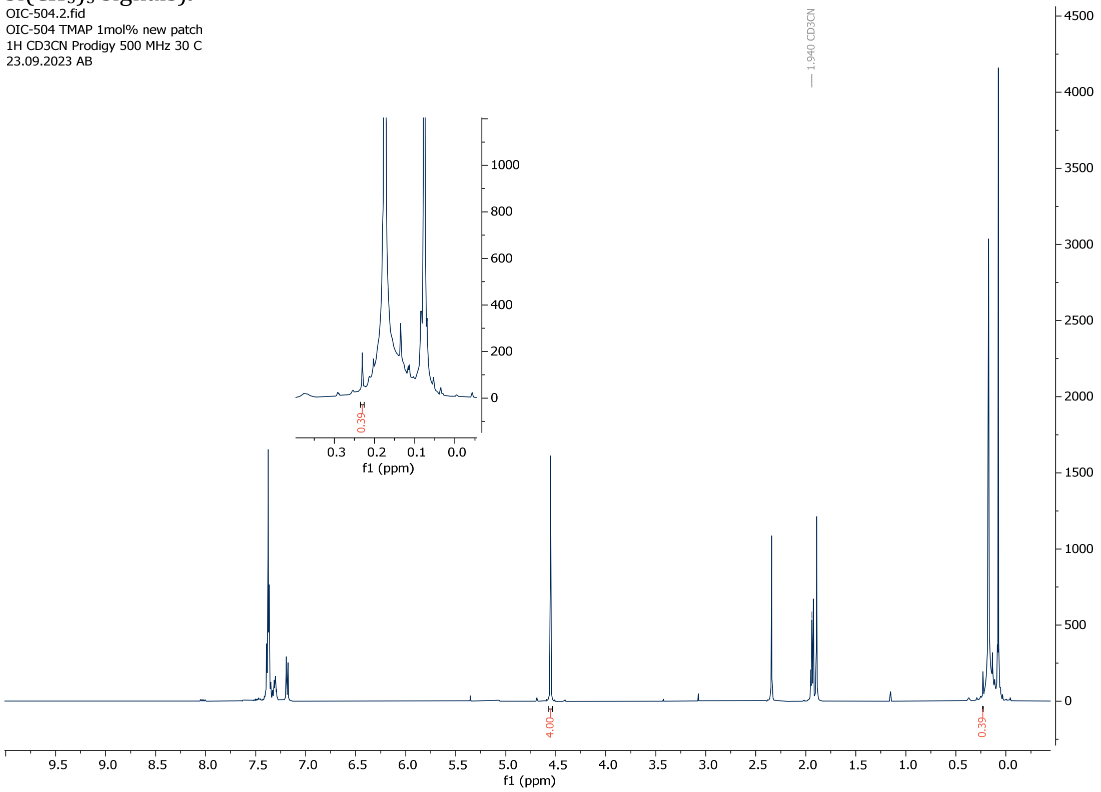

2. Last ^{1}H NMR scan of the reaction mixture, 68 min after TMAP addition (zoomed area – Ar-CC-Si(C***H***$_3$)$_3$ signals).

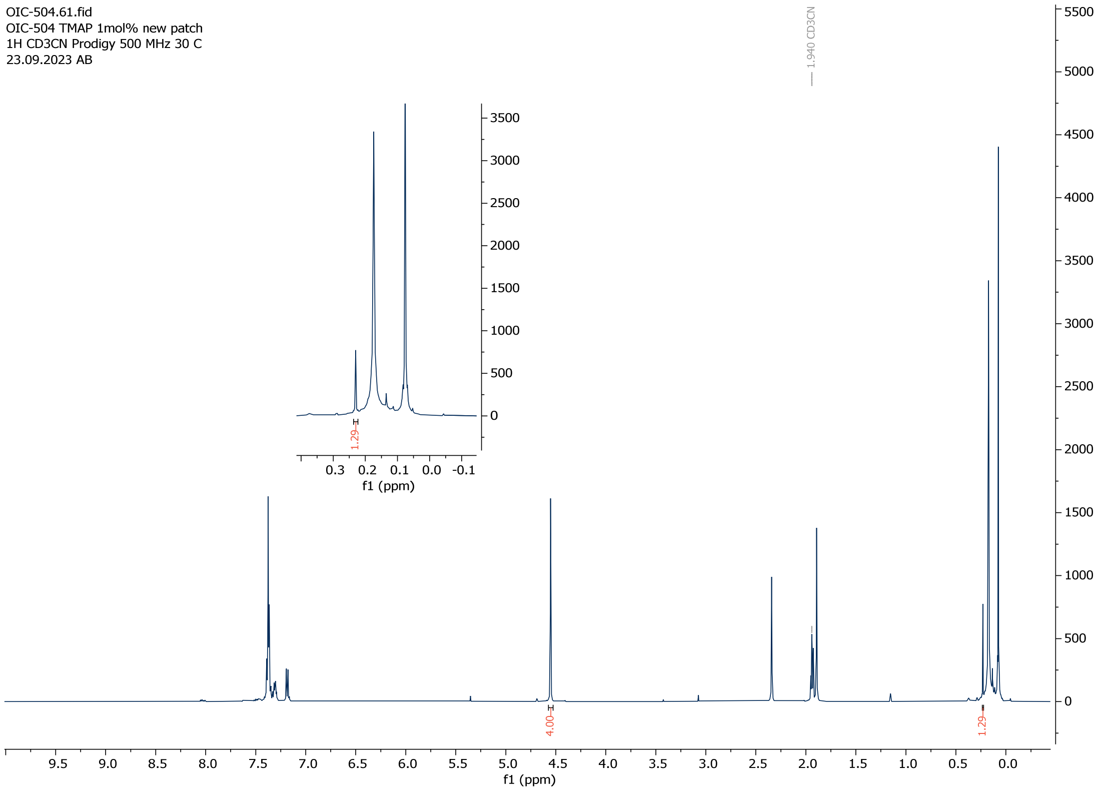

3. Experimental reaction profile and exponential fits for **2e** formation. The relative rates can be obtained from the exponentials (k_{Me} = 0.0246± 0.0021 mM min-1).

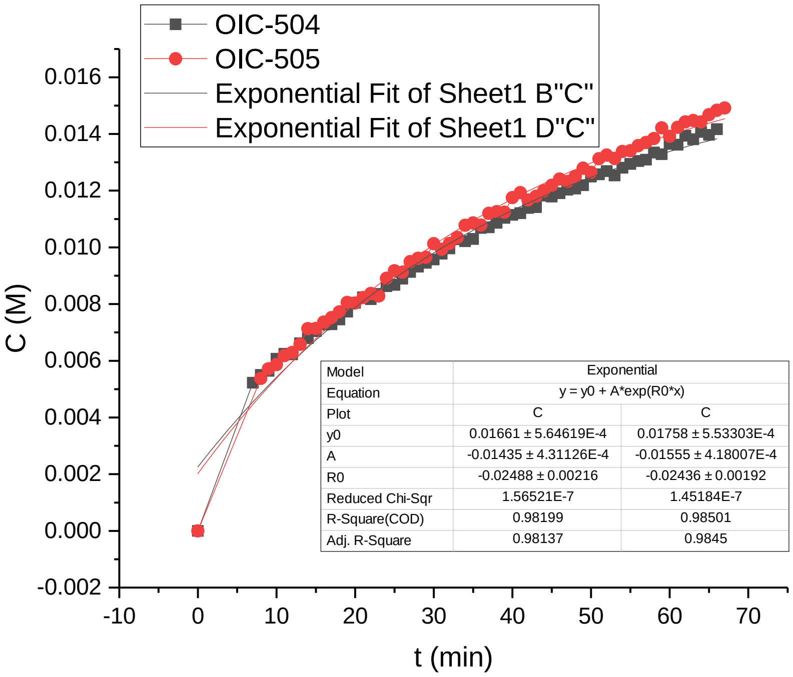

3.3.1.6 ^{1}H NMR experiment for **2d**

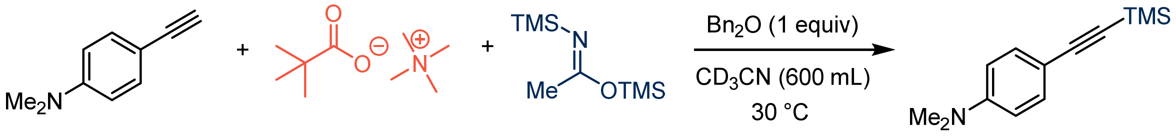

1d (60.00 μmol) **TMAP** (1 mol%) **BSA** (1.5 equiv) **2d**

1. First ^{1}H NMR scan of the reaction mixture, 9 min after TMAP addition (zoomed area – Ar-CC-Si(C***H***$_3$)$_3$ signals).

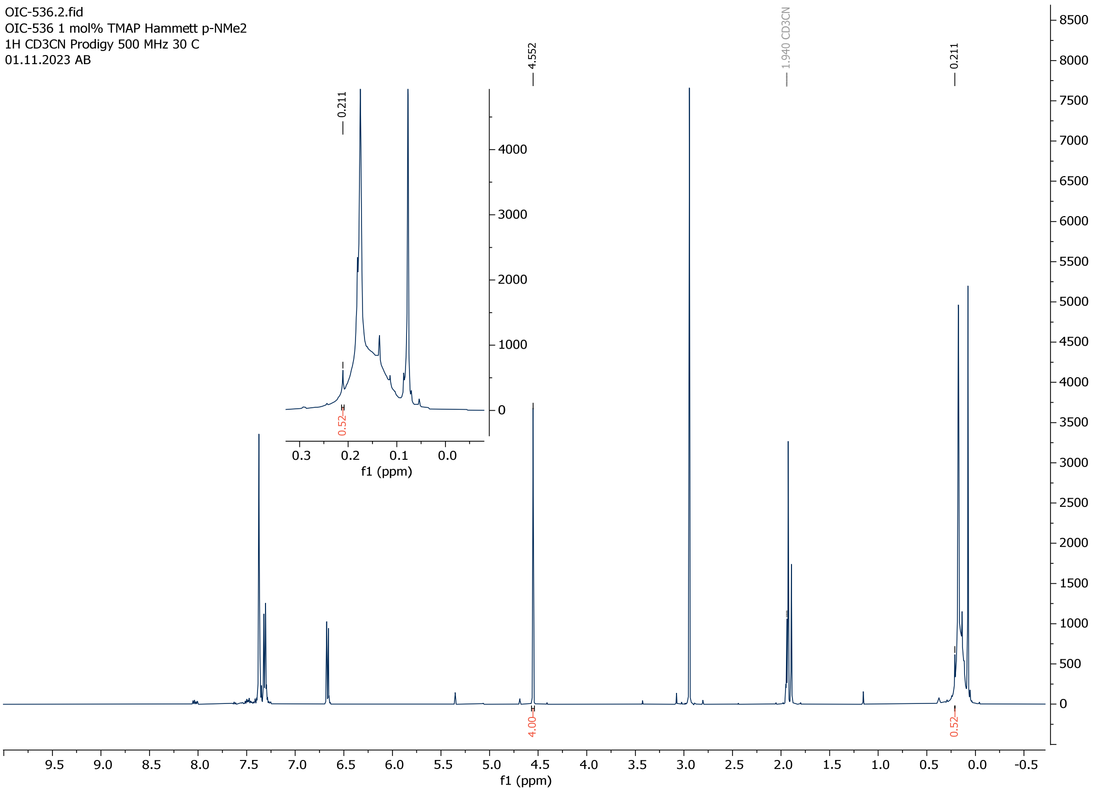

2. Last ^{1}H NMR scan of the reaction mixture, 68 min after TMAP addition (zoomed area – Ar-CC-Si(C***H***$_3$)$_3$ signals).

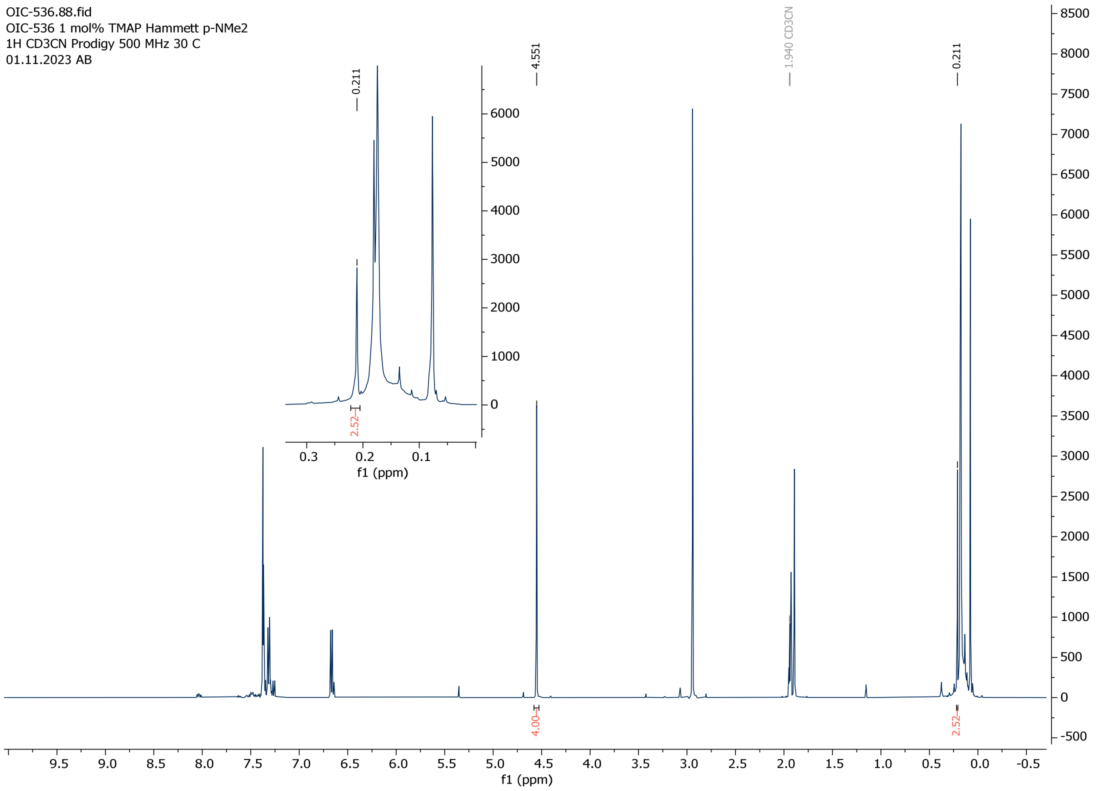

3. Experimental reaction profile and exponential fits for **2d** formation (reddish orange spots). The relative rates can be obtained from the exponentials (k_{NMe2} = 0.0040± 0.0004 mM min-1).

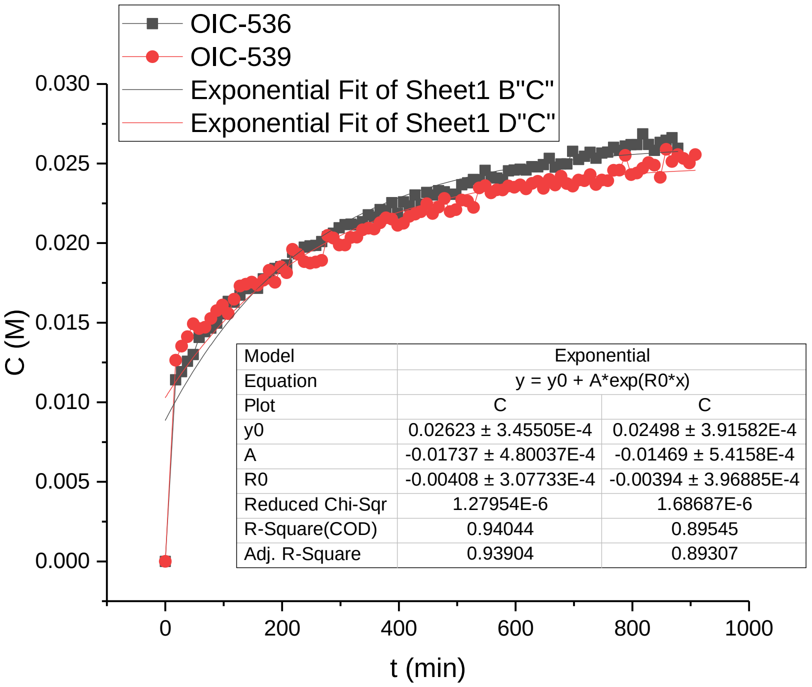

3.3.2 Hammett plot

The standard errors of the mean $\sigma_{\overline{rate}}$ for the average rates were calculated by Microsoft Excel from the standard deviations of the measurements.

$$\sigma_{\overline{rate}} = \frac{\sigma_{rate}}{\sqrt{N}}$$

Table S1. Reaction rates and their deviations.

Entry	1st run	2nd run	k_x
***p*-H**	0.05041	0.04832	0.049 ± 0.001
***p*-OMe**	0.02243	0.02844	0.025 ± 0.002
***p*-Me**	0.02488	0.02436	0.0246 ± 0.0002
***p*-F**	0.04674	0.05797	0.052 ± 0.004
***p*-Cl**	0.12660	0.13850	0.133 ± 0.004
***p*-NMe$_2$**	0.00408	0.00394	0.00401 ± 0.00005

Table S2. Hammett relationships.

Entry	σ_p	$\log(k_x/k_H)$	k_H	k_x
***p*-H**	0	0	0.049	0.049
***p*-OMe**	-0.268	-0.288	0.049	0.025
***p*-Me**	-0.170	-0.302	0.049	0.0246
***p*-F**	0.062	0.025	0.049	0.052
***p*-Cl**	0.227	0.429	0.049	0.133
***p*-NMe$_2$**	-0.830	-1.091	0.049	0.004

The rates were then used to generate a Hammett plot (Figure S1).

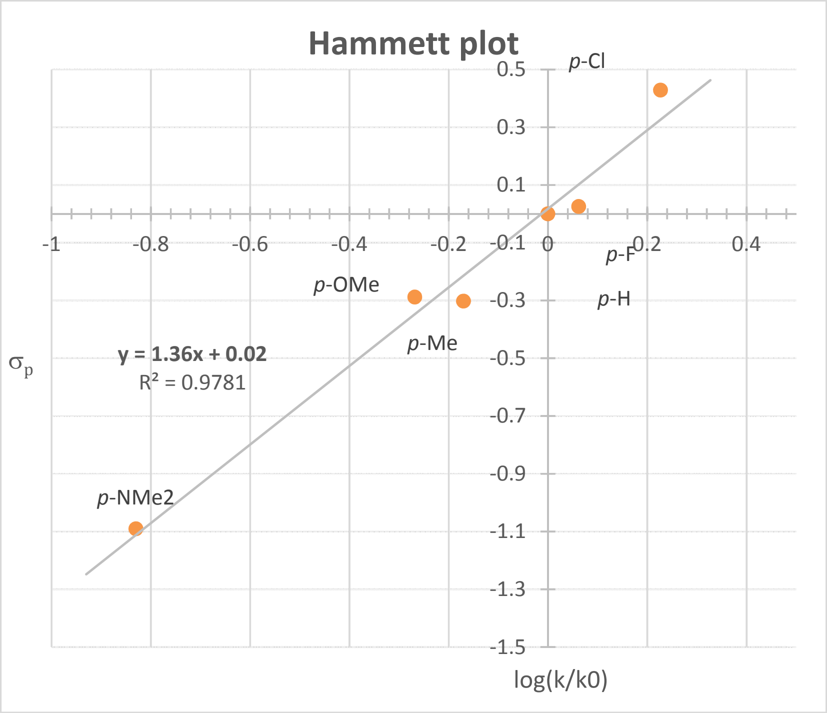

Equation	y = a + b*x
Plot	log(kx/kH)
Weight	No Weighting
Intercept	0.01745 ± 0.03821
Slope	1.36074 ± 0.10181
Residual Sum of Squares	0.02841
Pearson's r	0.98899
R-Square(COD)	0.9781
Adj. R-Square	0.97262

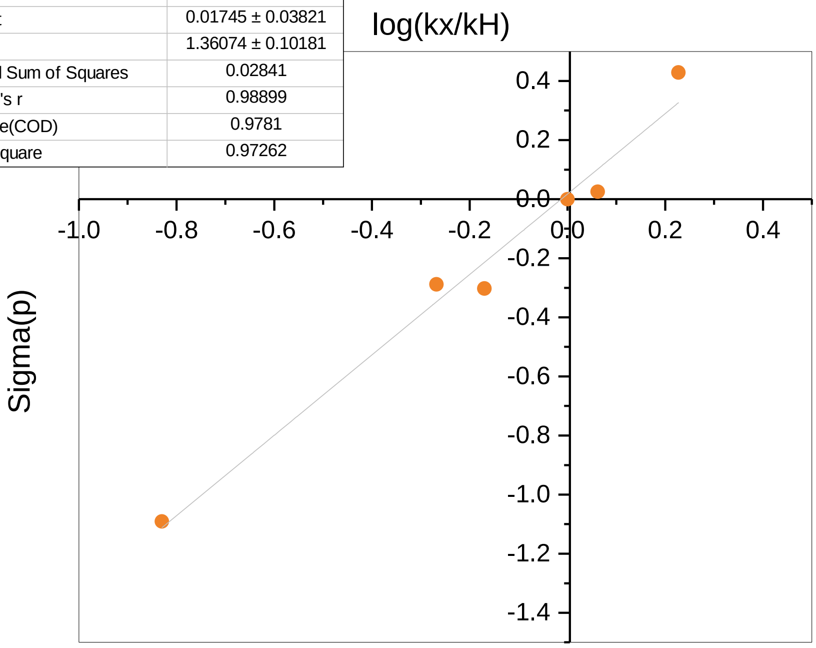

Figure S1. Hammett plots with different *p*-substituted arylacetylene substrates. The second plot (without labels) is from Origin Pro, showing the error of fit.

3.4 Crystallographic data

**Compound name**	**2x**
CCDC deposition number	2313380
Empirical formula	$C_{11}H_{21}NO_2Si$
Formula weight	227.35
Temperature (K)	262(50)
Crystal system, space group	Monoclinic, Cc
a, b, c (Å)	17.31037(13), 17.11040(15), 28.4095(2)
α, β, γ (°)	90, 95.0143(7), 90
Volume (Å^3)	8382.32(12)
Z	24
ρ_{calc} (g/cm^3)	1.081
μ (mm^{-1})	1.361
F(000)	2976.0
Crystal size (mm^3)	0.17 × 0.1 × 0.07
Diffractometer	XtaLAB Synergy R
Detector	HyPix-Arc 100
Radiation	Cu Kα (λ = 1.54184)
2Θ range for data collection (°)	6.246 to 133.496
Index ranges	-20 ≤ h ≤ 20, -20 ≤ k ≤ 20, -33 ≤ l ≤ 33
Reflections collected	76041
Independent reflections	13944 [R_{int} = 0.0432, R_{sigma} = 0.0307]
Data/restraints/parameters	13944/2/872
Goodness-of-fit on F^2	1.053
Final R indexes [I>=2σ (I)]	R_1 = 0.0324, wR_2 = 0.0855
Final R indexes [all data]	R_1 = 0.0345, wR_2 = 0.0866
Largest diff. peak/hole (e Å^{-3})	0.28/-0.22
Absorption correction	Empirical absorption correction using spherical

	harmonics, implemented in SCALE3 ABSPACK scaling algorithm.
Refinement description	All non-hydrogen atoms were refined anisotropicaly. The hydrogen atoms were calculated to their idealised positions as riding atoms with isotropic thermal parameters as 1.2 x C for C(H,H) and 1.5 x C for C(H,H,H). Amide N(H) proton was located from the electron density map and refined isotopically.
T_{min}, T_{max}	0.69139, 1.00000
Flack parameter	0.023(8)

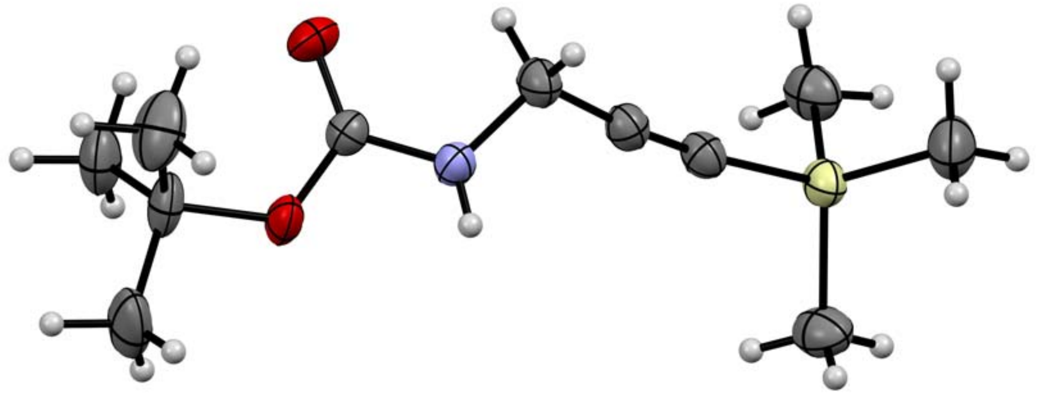

Figure S2. The ORTEP plot of **2x.** The thermal displacement parameters are shown at 50% probability level.

**Compound name**	**2v**
CCDC deposition number	2313373
Empirical formula	$C_{29}H_{48}O_2Si_3$
Formula weight	512.94
Temperature (K)	120.01(10)
Crystal system, space group	Orthorhombic, $P2_12_12_1$
a, b, c (Å)	7.05206(17),17.6437(4), 24.7049(7)
α, β, γ (°)	90, 90, 90
Volume (Å^3)	3073.90(13)
Z	4
ρ_{calc} (g/cm^3)	1.108
μ (mm^{-1})	1.582
F(000)	1120.0
Crystal size (mm^3)	0.111 × 0.08 × 0.044
Diffractometer	XtaLAB Synergy R
Detector	HyPix-Arc 100
Radiation	Cu Kα (λ = 1.54184)
2Θ range for data collection (°)	6.156 to 158.854
Index ranges	-7 ≤ h ≤ 8, -22 ≤ k ≤ 22, -31 ≤ l ≤ 30
Reflections collected	24251
Independent reflections	6339 [R_{int} = 0.0556, R_{sigma} = 0.0505]
Data/restraints/parameters	6339/0/317
Goodness-of-fit on F^2	1.047
Final R indexes [I>=2σ (I)]	R_1 = 0.0463, wR_2 = 0.1103
Final R indexes [all data]	R_1 = 0.0569, wR_2 = 0.1162
Largest diff. peak/hole (e Å^{-3})	0.23/-0.35
Absorption correction	Empirical absorption correction using spherical harmonics, implemented in SCALE3 ABSPACK scaling algorithm.

Refinement description	All non-hydrogen atoms were refined anisotropicaly. The hydrogen atoms were calculated to their idealised positions as riding atoms with isotropic thermal parameters as 1.2 x C for C(H), C(H,H) and 1.5 x C for C(H, H, H).
T_{min}, T_{max}	0.88474, 1.00000
Flack parameter	-0.01(2)

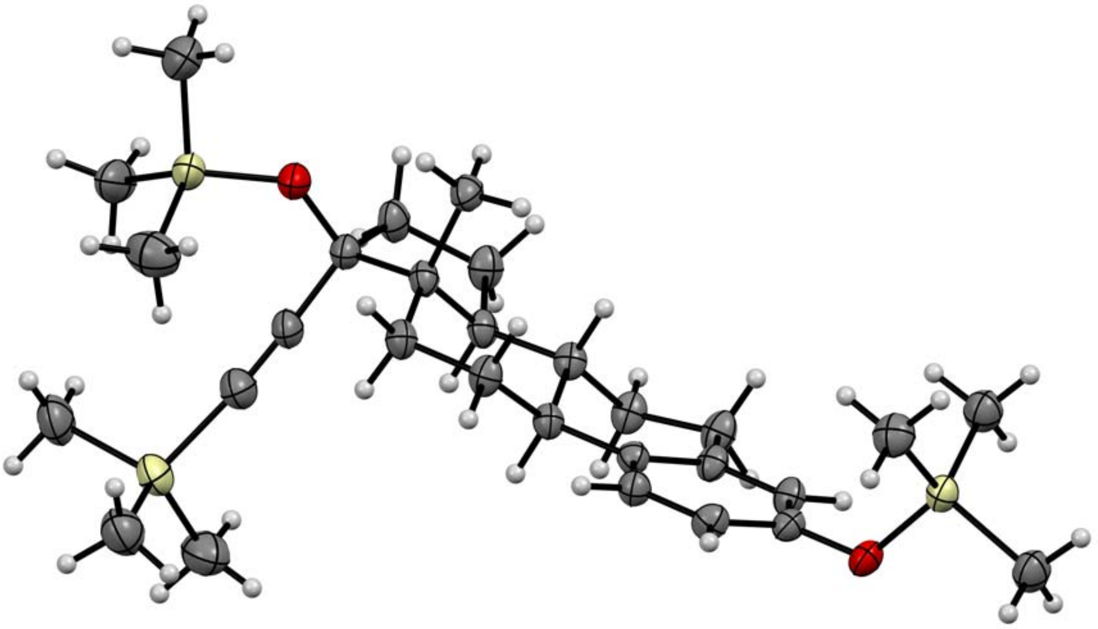

Figure S3. The ORTEP plot of **2v.** The thermal displacement parameters are shown at 50% probability level.

3.5 References

[1] Bellotti, P.; Rogge, T.; Paulus, F.; Laskar, R.; Rendel, N.; Ma, J.; Houk, K. N.; Glorius F. Visible-Light Photocatalyzed *peri*-(3 + 2) Cycloadditions of Quinolines. *J. Am. Chem. Soc.* **2022**, *144*, 34, 15662–15671. DOI: 10.1021/jacs.2c05687

[2] Turnu, F.; Luridiana, A.; Cocco, A.; Porcu, S.; Frongia, A.; Sarais, G.; Secci F. Catalytic Tandem Friedel–Crafts Alkylation/C4–C3 Ring-Contraction Reaction: An Efficient Route for the Synthesis of Indolyl Cyclopropanecarbaldehydes and Ketones. *Org. Lett.* **2019**, *21*, 18, 7329–7332. DOI: 10.1021/acs.orglett.9b02617

[3] Molander, G.; Cadoret F. Synthesis of the Stereogenic Triad of the Halicyclamine A Core. *Tetrahedron Lett.* **2011**, *52*, 17, 2199-2202. DOI: 10.1016/j.tetlet.2010.11.162

[4] Zhou, Y.; Porco, J. A.; Snyder. J. K. Synthesis of 5,6,7,8-Tetrahydro-1,6-naphthyridines and Related Heterocycles by Cobalt-Catalyzed [2 + 2 + 2] Cyclizations. *Org. Lett.* **2007**, *9*, 3, 393–396. DOI: 10.1021/ol0625280

[5] Romashov, L. V.; Ananikov, V. P. Alkynylation of Bio-Based 5-Hydroxymethylfurfural to Connect Biomass Processing with Conjugated Polymers and Furanic Pharmaceuticals. *Chem. Asian J.* **2017**, *12*, 2652. DOI: 10.1002/asia.201700940

[6] Helmecke, L.; Spittler, M.; Schmidt, B. M.; Czekelius, C. Metal-Free Iodoperfluoroalkylation: Photocatalysis versus Frustrated Lewis Pair Catalysis. *Synthesis* **2021**, *53*, 1, 123-134. DOI: 10.1055/s-0040-1707232

[7] Upadhyay, N. S.; Jayakumara, J.; Cheng, C.-H. Facile one-pot synthesis of 2,3-dihydro-1*H*-indolizinium derivatives by rhodium(III)-catalyzed intramolecular oxidative annulation *via* C–H activation: application to ficuseptine synthesis. *Chem. Commun.* **2017**, *53*, 2491-2494. DOI: 10.1039/C7CC00008A

[8] CrysAlisPro 1.171.42.80a, **2023**, Rigaku Oxford Diffraction.

[9] Sheldrick, G.M. (**2015**). *Acta Cryst.* A71, 3-8.

[10] Dolomanov, O.V., Bourhis, L.J., Gildea, R.J, Howard, J.A.K. & Puschmann, H. (**2009**), *J. Appl. Cryst.* 42, 339-341.

[11] Sheldrick, G.M. (**2015**). *Acta Cryst.* C71, 3-8.

[12] Mercury 2022.3.0 (Build 364735).

[13] Riuttamäki, S.; Laczkó, G.; Madarász, Á.; Földes, T.; Pápai, I.; Bannykh, A.; Pihko, P. M. Carboxylate Catalyzed Isomerization of β,γ-Unsaturated *N*-Acetylcysteamine Thioesters. *Chem. Eur. J.* **2022**, *28*, e202201030. DOI: 10.1002/chem.202201030

[14] Kuciński K.; Hreczycho G. Transition Metal-Free Catalytic C–H Silylation of Terminal Alkynes with bis(Trimethylsilyl)acetylene Initiated by KHMDS. *Chem. Cat. Chem* **2022**, *14*, e202200794. DOI: 10.1002/cctc.202200794

[15] He T.; Qu Z.-W.; Klare H. F. T.; Grimme S.; Oestreich M. Intermolecular Carbosilylation of α-Olefins with C(sp3)–C(sp) Bond Formation Involving Silylium-Ion Regeneration. *Angew. Chem. Int. Ed.* **2022**, *61*, e202203347. DOI: 10.1002/anie.202203347

[16] Harada Y.; Nakanishi J.; Fujihara H.; Tobisu M.; Fukumoto Y.; Chatani N. Rh(I)-Catalyzed Carbonylative Cyclization Reactions of Alkynes with 2-Bromophenylboronic Acids Leading to Indenones. *J. Am. Chem. Soc.* **2007**, *129*, 17, 5766–5771. DOI: 10.1021/ja070107n

[17] Medina I.-M. R.; Rohdenburg M.; Lork E.; Staubitz A. Aggregation Induced Emission – Emissive Stannoles in the Solid State. *Chem. Commun*. **2020**, *56*, 9775-9778. DOI: 10.1039/D0CC04525J.

[18] Štěpnička P.; Gyepes R.; Císařová I.; Varga V.; Polášek M.; Horáček M.; Mach K. Synthesis and Structure of Titanocene Complexes with η^2-Coordinated Internal Ferrocenylacetylenes. *Organometallics* **1999**, *18*, 4, 627–633. DOI: 10.1021/om980832u

[19] Hachiya S.; Asai K.; Konishi G. Unique Solvent-Dependent Fluorescence of Nitro-Group-Containing Naphthalene Derivatives with Weak Donor–Strong Acceptor System. *Tet. Lett.* **2013**, *54*, 1839–1841. DOI: 10.1016/j.tetlet.2013.01.096

[20] Arde P.; Reddy V.; Vijaya A. R. NHC catalysed trimethylsilylation of terminal alkynes and indoles with Ruppert's reagent under solvent free conditions. *RSC Adv.*, **2014**, *4*, 49775-497 10. DOI: 1039/C4RA08727E

[21] Cahiez, G.; Gager, O.; Buendia, J. Copper-Catalyzed Cross-Coupling of Alkyl and Aryl Grignard Reagents with Alkynyl Halides. *Angewandte Chemie International Edition*, **2010**, *49*, 1278-1281. https://doi.org/10.1002/anie.200905816

[22] Ye F.; Ma X.; Xiao Q.; Li H.; Zhang Y.; Wang J. C(sp)–C(sp^3) Bond Formation through Cu-Catalyzed Cross-Coupling of *N*-Tosylhydrazones and Trialkylsilylethynes. *J. Am. Chem. Soc.* **2012**, *134*, 13, 5742–5745.

[23] Varela J. A.; Castedo L.; Saá C. Synthesis of Annelated Substituted Bipyridines and Terpyridines by Cobalt(I)-Catalyzed [2 + 2 + 2] Cycloaddition. *J. Org. Chem.* **1997**, *62*, 12, 4189–4192. DOI: 10.1021/jo9618886

[24] Peters R. H.; Crowe D. F.; Tanabe M.; Avery M. A.; Chong W. K. M. Steroidal silicon side-chain analogs as potential antifertility agents. *J. Med. Chem.* **1987**, *30* (4), 646-652. DOI: 10.1021/jm00387a011

[25] Wissing M., Studer A., Tuning the Selectivity of AuPd Nanoalloys towards Selective Dehydrogenative Alkyne Silylation. *Chem. Eur. J.* **2019**, *25*, 5870.
