# Supplementary material for: Carboxylate-Catalyzed C-Silylation of Terminal Alkynes
Source: Org Lett. 2024 Mar 1;26(10):1991–5. doi: 10.1021/acs.orglett.3c04213 (PMC10949233; doi:10.1021/acs.orglett.3c04213)

Supporting Information, Part II: NMR spectra

Carboxylate catalyzed *C*-silylation of terminal alkynes

Anton Bannykh, Petri M. Pihko *

Department of Chemistry and Nanoscience Center,. P.O.B. 35, 40014 University of Jyväskylä, FINLAND

E-mail: Petri.Pihko@jyu.fi

Contents

1.1 Trimethyl(phenylethynyl)silane (2a), ^{1}H NMR spectrum ... 5
1.2 Trimethyl(phenylethynyl)silane (2a), ^{13}C NMR spectrum ... 6
1.3 Trimethyl((4-(trifluoromethyl)phenyl)ethynyl)silane (2b), ^{1}H NMR spectrum ... 7
1.4 Trimethyl((4-(trifluoromethyl)phenyl)ethynyl)silane (2b), ^{13}C NMR spectrum ... 8
1.5 ((4-Methoxyphenyl)ethynyl)trimethylsilane (2c), ^{1}H NMR spectrum ... 9
1.6 ((4-Methoxyphenyl)ethynyl)trimethylsilane (2c), ^{13}C NMR spectrum ... 10
1.7 *N,N*-Dimethyl-4-((trimethylsilyl)ethynyl)aniline (2d), ^{1}H NMR spectrum ... 11
1.8 *N,N*-Dimethyl-4-((trimethylsilyl)ethynyl)aniline (2d), ^{13}C NMR spectrum ... 12
1.9 Trimethyl(*p*-tolylethynyl)silane (2e), ^{1}H NMR spectrum ... 13
1.10 Trimethyl(*p*-tolylethynyl)silane (2e), ^{13}C NMR spectrum ... 14
1.11 ((4-Fluorophenyl)ethynyl)trimethylsilane (2f), ^{1}H NMR spectrum ... 15
1.12 ((4-Fluorophenyl)ethynyl)trimethylsilane (2f), ^{13}C NMR spectrum ... 16
1.13 ((4-chlorophenyl)ethynyl)trimethylsilane (2g), ^{1}H NMR spectrum ... 17
1.14 ((4-chlorophenyl)ethynyl)trimethylsilane (2g), ^{13}C NMR spectrum ... 18
1.15 ((2-Chlorophenyl)ethynyl)trimethylsilane (2h), ^{1}H NMR spectrum ... 19
1.16 ((2-Chlorophenyl)ethynyl)trimethylsilane (2h), ^{13}C NMR spectrum ... 20
1.17 ((3,5-Bis(trifluoromethyl)phenyl)ethynyl)trimethylsilane (2i), ^{1}H NMR spectrum ... 21
1.18 ((3,5-Bis(trifluoromethyl)phenyl)ethynyl)trimethylsilane (2i), ^{13}C NMR spectrum ... 22
1.19 Ferrocenylethynetrimethylsilane (2j), ^{1}H NMR spectrum ... 23
1.20 Ferrocenylethynetrimethylsilane (2j), ^{13}C NMR spectrum ... 24

1.21 3-((Trimethylsilyl)ethynyl)pyridine (2k), ^{1}H NMR spectrum 25
1.22 3-((Trimethylsilyl)ethynyl)pyridine (2k), ^{13}C NMR spectrum 26
1.23 Trimethyl(thiophen-3-ylethynyl)silane (2l), ^{1}H NMR spectrum 27
1.24 Trimethyl(thiophen-3-ylethynyl)silane (2l), ^{13}C NMR spectrum 28
1.25 ((6-methoxynaphthalen-2-yl)ethynyl)trimethylsilane (2m), ^{1}H NMR spectrum 29
1.26 ((6-methoxynaphthalen-2-yl)ethynyl)trimethylsilane (2m), ^{13}C NMR spectrum 30
1.27 *tert*-Butyldimethyl((3-(trimethylsilyl)prop-2-yn-1-yl)oxy)silane (2n), ^{1}H NMR spectrum 31
1.28 *tert*-Butyldimethyl((3-(trimethylsilyl)prop-2-yn-1-yl)oxy)silane (2n), ^{13}C NMR spectrum 32
1.29 Trimethyl(3-(oxiran-2-ylmethoxy)prop-1-yn-1-yl)silane (2o), ^{1}H NMR spectrum 33
1.30 Trimethyl(3-(oxiran-2-ylmethoxy)prop-1-yn-1-yl)silane (2o), ^{13}C NMR spectrum 34
1.31 1-(3-(trimethylsilyl)prop-2-yn-1-yl)-1H-indole (2p), ^{1}H NMR spectrum 35
1.32 *1-(3-(trimethylsilyl)prop-2-yn-1-yl)-1H-indole* (2p), ^{13}C NMR spectrum 36
1.33 (Cyclohex-1-en-1-ylethynyl)trimethylsilane (2q), ^{1}H NMR spectrum 37
1.34 (Cyclohex-1-en-1-ylethynyl)trimethylsilane (2q), ^{13}C NMR spectrum 38
1.35 Hex-1-yn-1-yltrimethylsilane (2r), ^{1}H NMR spectrum 39
1.36 Hex-1-yn-1-yltrimethylsilane (2r), ^{13}C NMR spectrum 40
1.37 Trimethyl(4-phenylbut-1-yn-1-yl)silane (2s), ^{1}H NMR spectrum 41
1.38 Trimethyl(4-phenylbut-1-yn-1-yl)silane (2s), ^{13}C NMR spectrum 42
1.39 6-(Trimethylsilyl)hex-5-ynenitrile (2t), ^{1}H NMR spectrum 43
1.40 6-(Trimethylsilyl)hex-5-ynenitrile (2t), ^{13}C NMR spectrum 44
1.41 1,3-Bis((trimethylsilyl)ethynyl)benzene (2u), ^{1}H NMR spectrum 45
1.42 1,3-Bis((trimethylsilyl)ethynyl)benzene (2u), ^{13}C NMR spectrum 46
1.43 (((*8R,9S,13S,14S,17S*)-13-Methyl-17-((trimethylsilyl)ethynyl)-7,8,9,11,12,13,14,15,16,17-decahydro-6H-cyclopenta[*a*]phenanthrene-3,17-diyl)bis(oxy))bis(trimethylsilane) (2v), ^{1}H NMR spectrum. (*) indicates impurity (bis-*O*-silylated product with free alkyne-C-H) 47

1.44 (((*8R,9S,13S,14S,17S*)-13-Methyl-17-((trimethylsilyl)ethynyl)-7,8,9,11,12,13,14,15,16,17-decahydro-6H-cyclopenta[*a*]phenanthrene-3,17-diyl)bis(oxy))bis(trimethylsilane) (2v), ^{13}C NMR spectrum....48

1.45 *tert*-Butyl (trimethylsilyl)(3-(trimethylsilyl)prop-2-yn-1-yl)carbamate (2w), ^{1}H NMR spectrum....49

1.46 *tert*-Butyl (trimethylsilyl)(3-(trimethylsilyl)prop-2-yn-1-yl)carbamate (2w), ^{13}C NMR spectrum....50

1.47 *tert*-Butyl (3-(trimethylsilyl)prop-2-yn-1-yl)carbamate (2x), ^{1}H NMR spectrum....51

1.48 *tert*-Butyl (3-(trimethylsilyl)prop-2-yn-1-yl)carbamate (2x), ^{13}C NMR spectrum....52

1.49 *tert*-Butyldimethyl(phenylethynyl)silane (4), ^{1}H NMR spectrum....53

1.50 *tert*-Butyldimethyl(phenylethynyl)silane (4), ^{13}C NMR spectrum....54

1.1 Trimethyl(phenylethynyl)silane (**2a**), ^{1}H NMR spectrum

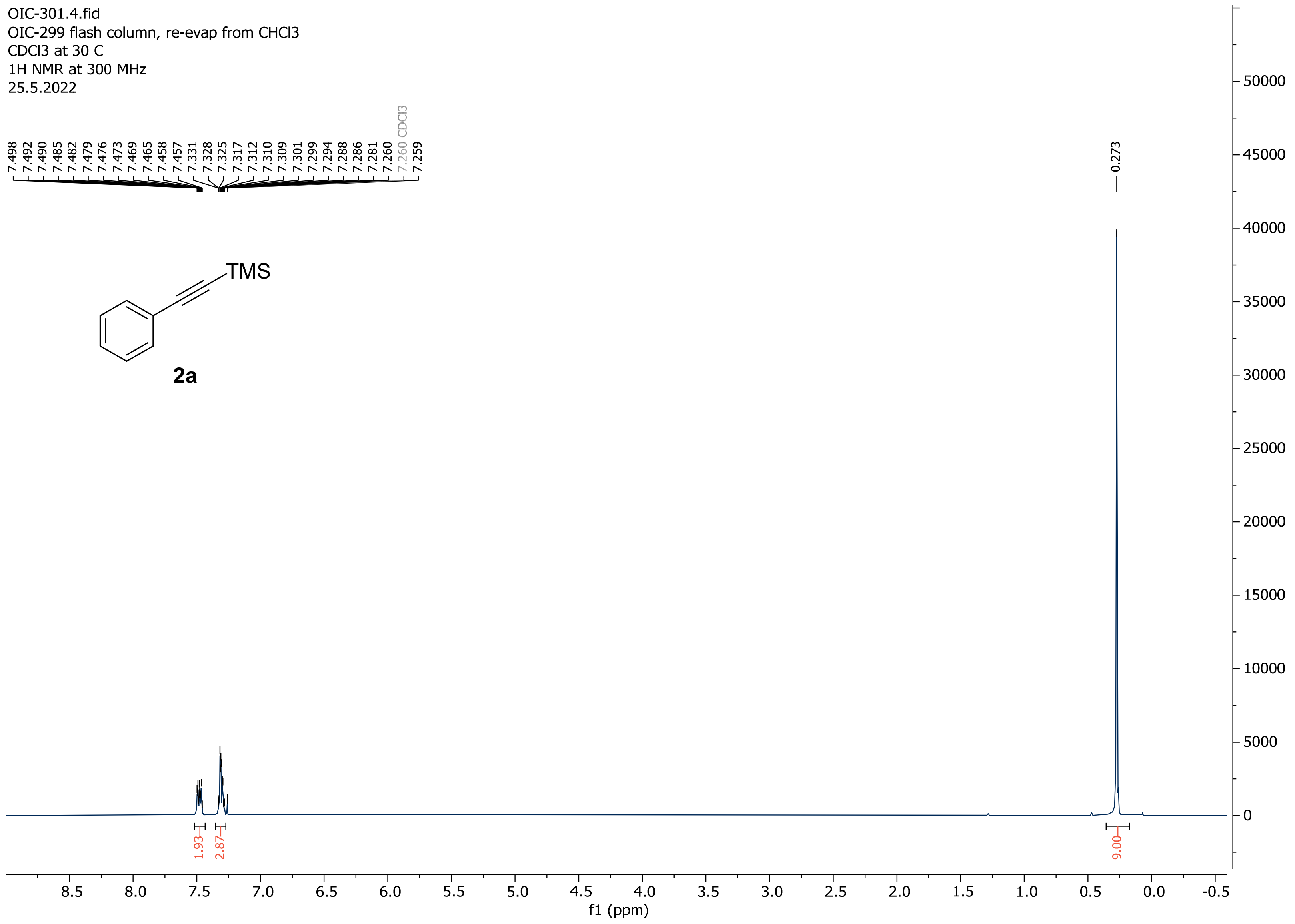

1.2 Trimethyl(phenylethynyl)silane (**2a**), ^{13}C NMR spectrum

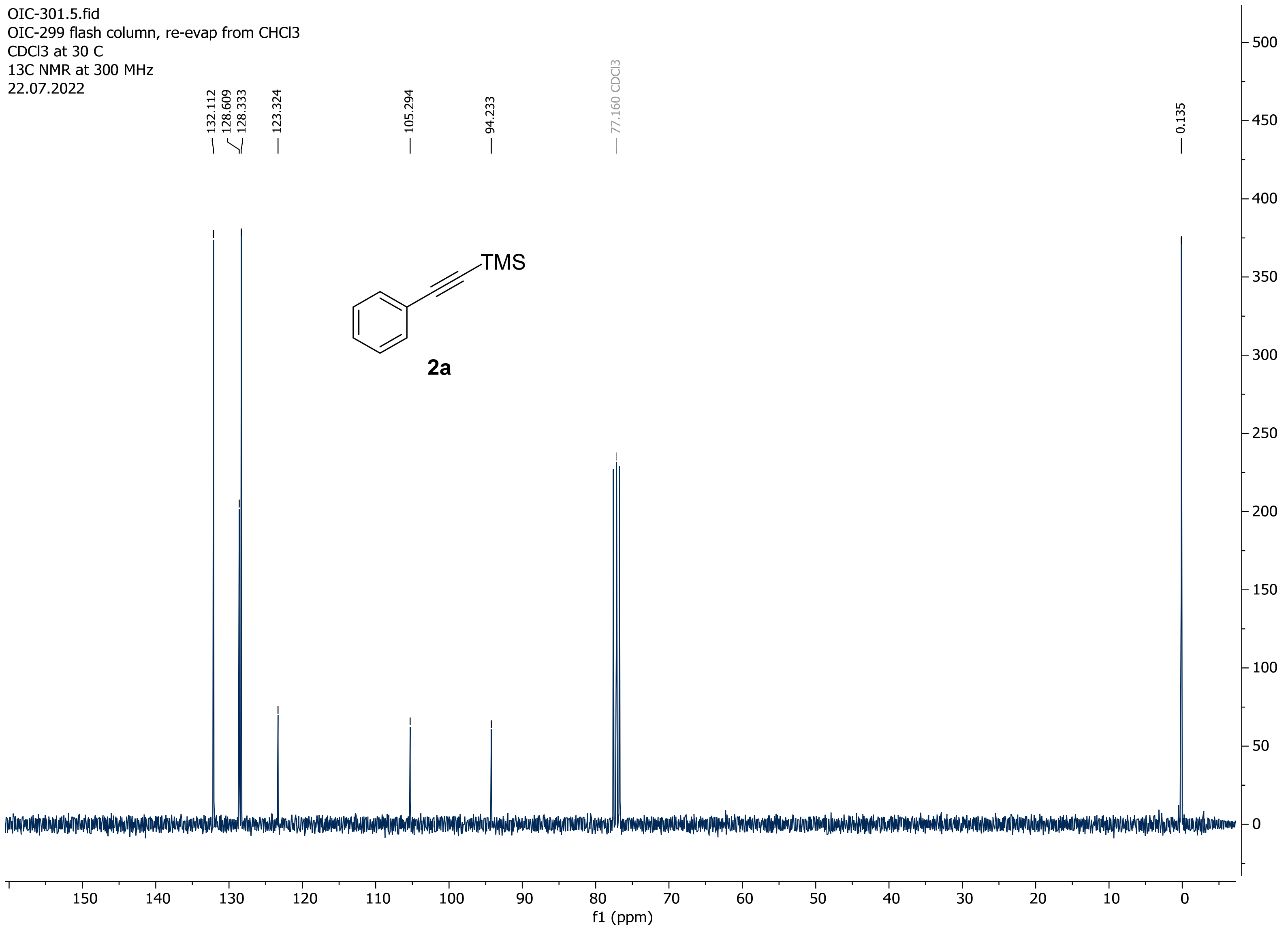

1.3 Trimethyl((4-(trifluoromethyl)phenyl)ethynyl)silane (**2b**), ^{1}H NMR spectrum

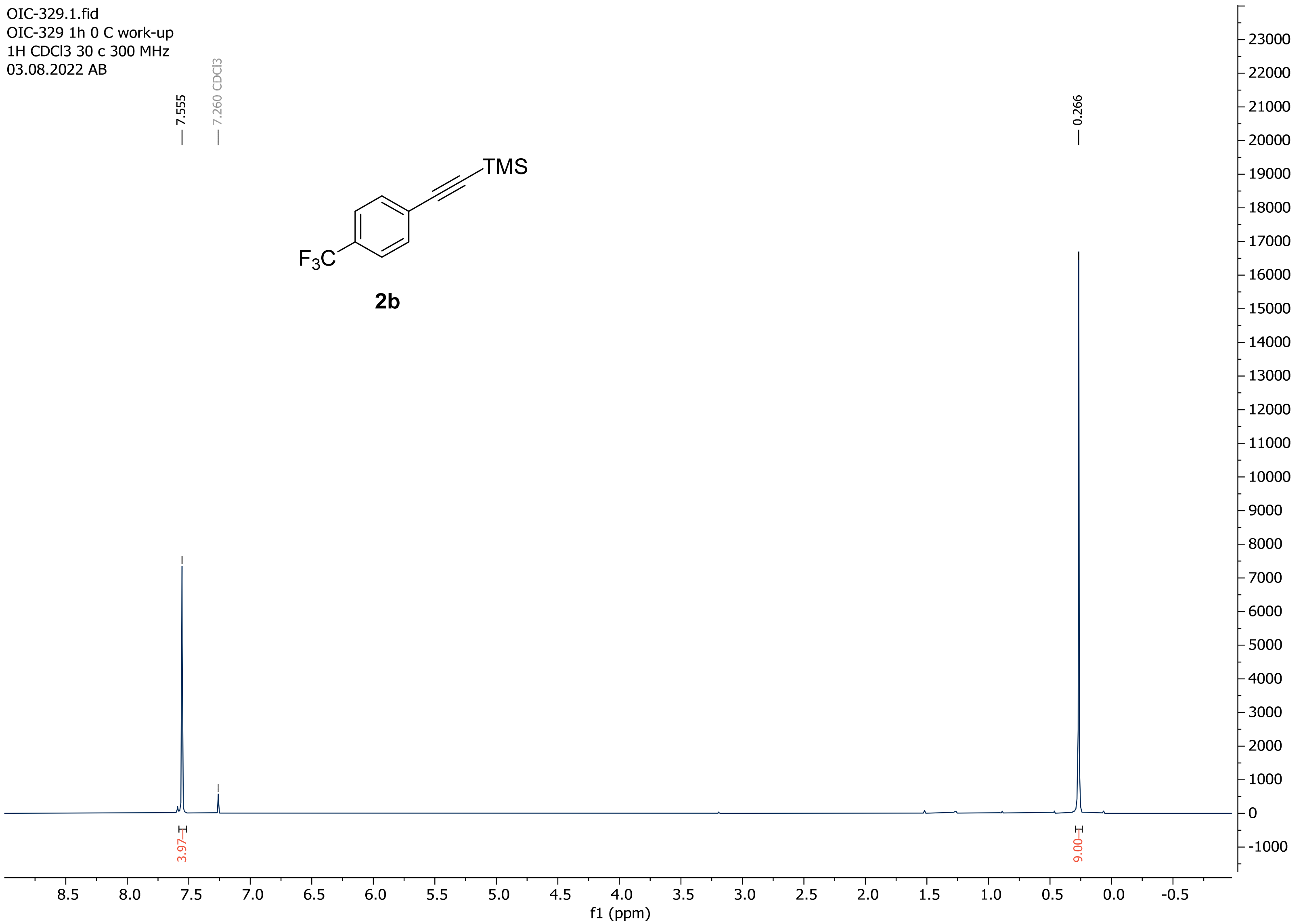

1.4 Trimethyl((4-(trifluoromethyl)phenyl)ethynyl)silane (**2b**), ^{13}C NMR spectrum

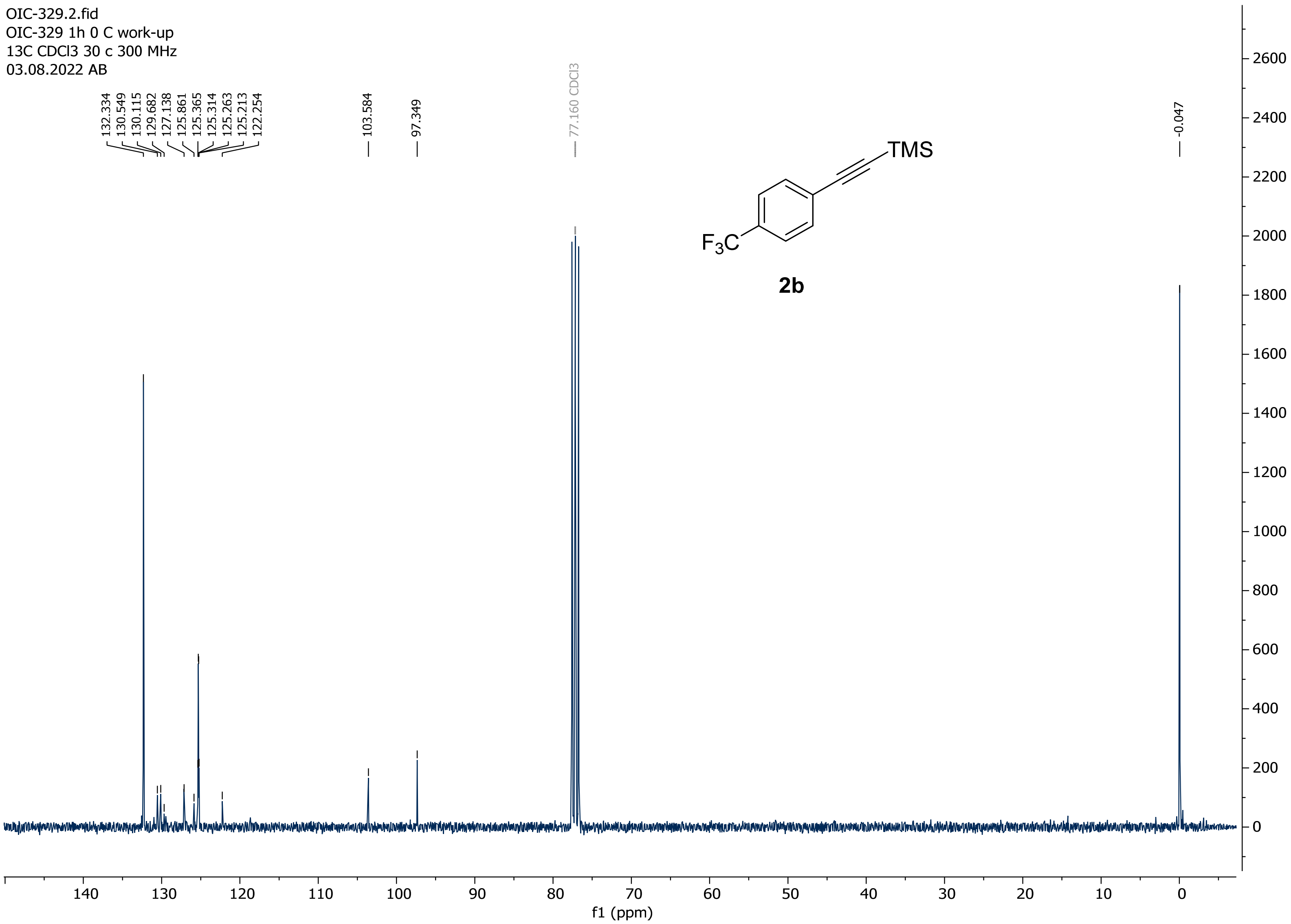

1.5 ((4-Methoxyphenyl)ethynyl)trimethylsilane (**2c**), ^{1}H NMR spectrum

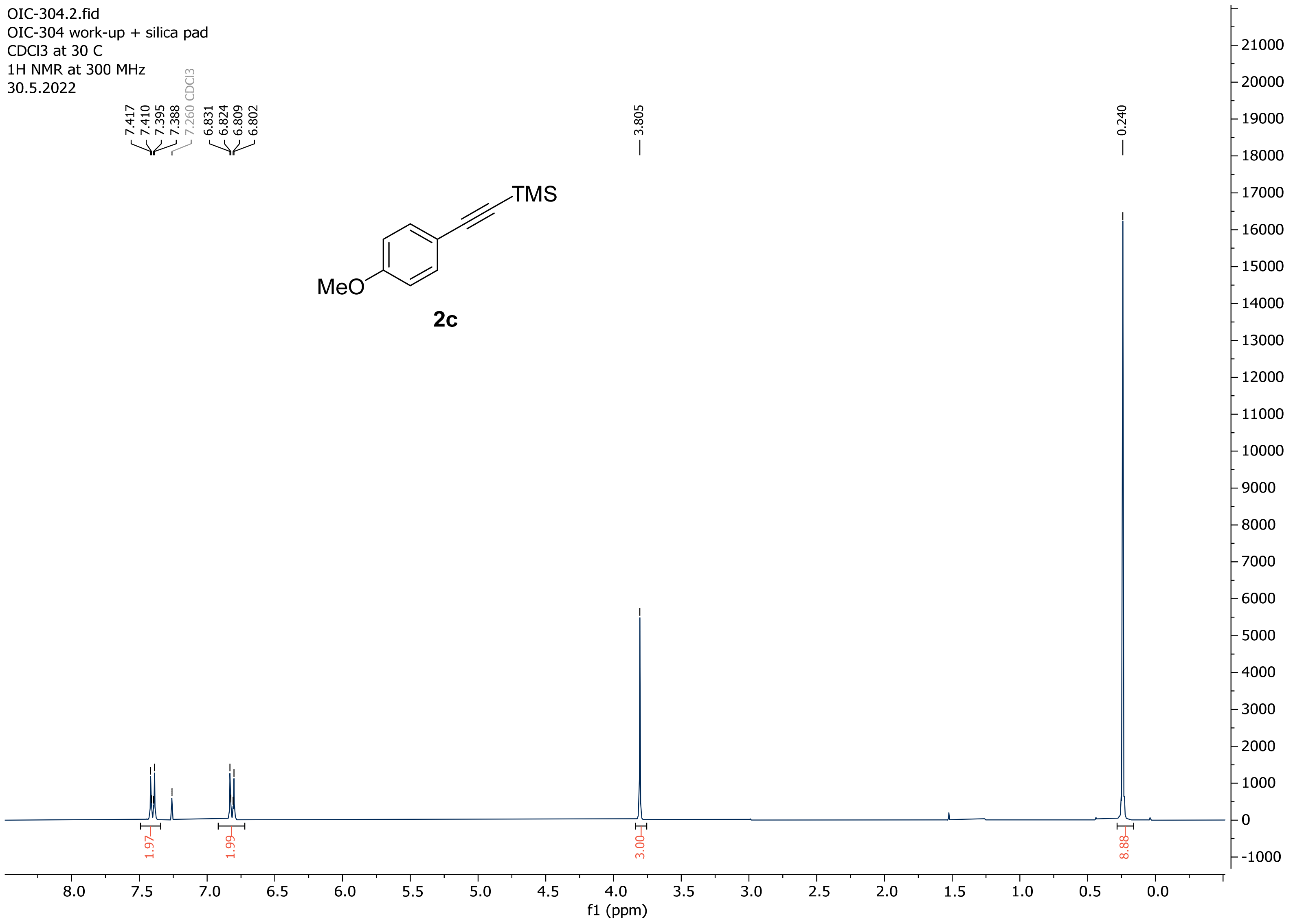

1.6 ((4-Methoxyphenyl)ethynyl)trimethylsilane (**2c**), ^{13}C NMR spectrum

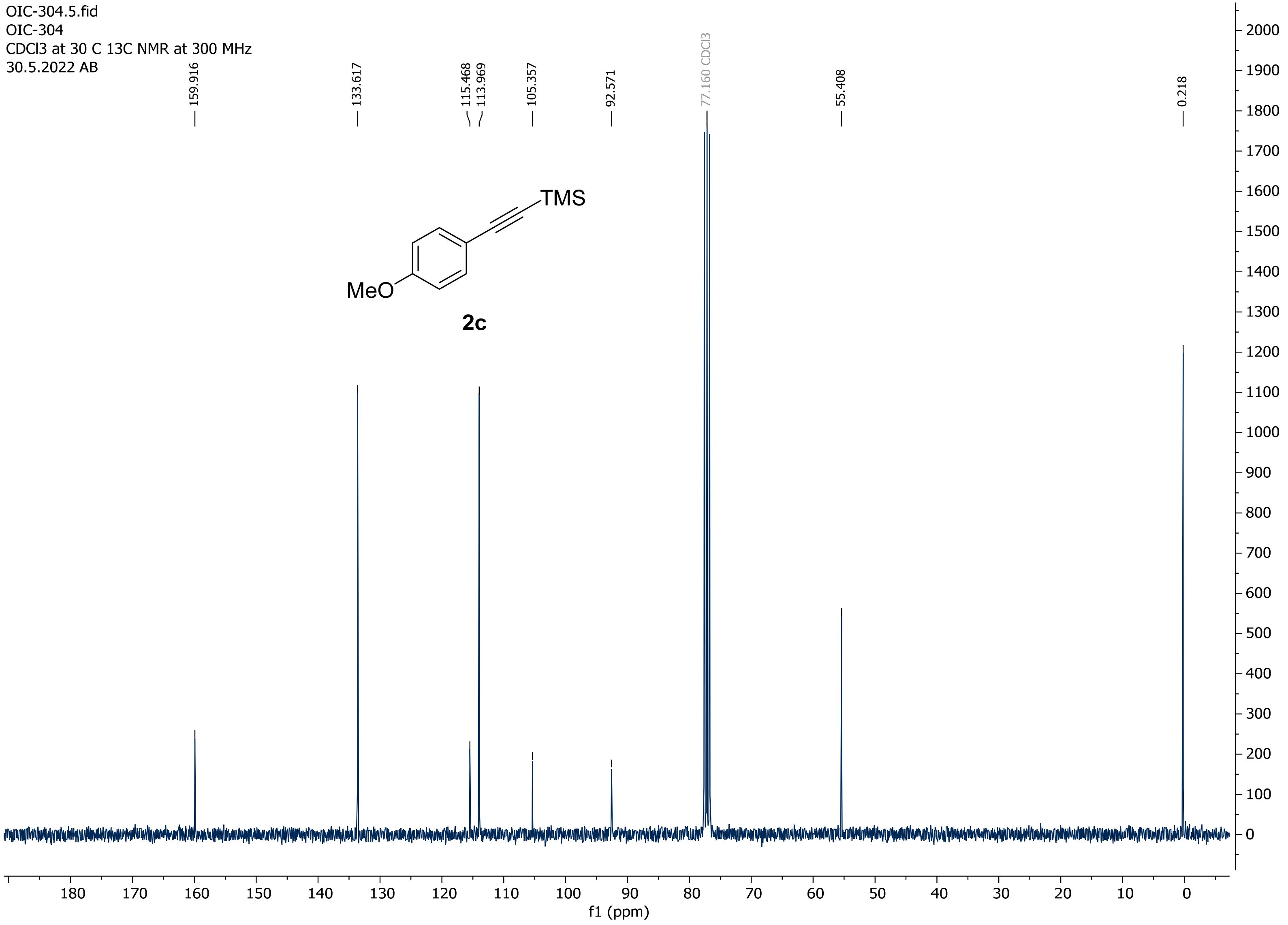

1.7 *N,N*-Dimethyl-4-((trimethylsilyl)ethynyl)aniline (**2d**), ^{1}H NMR spectrum

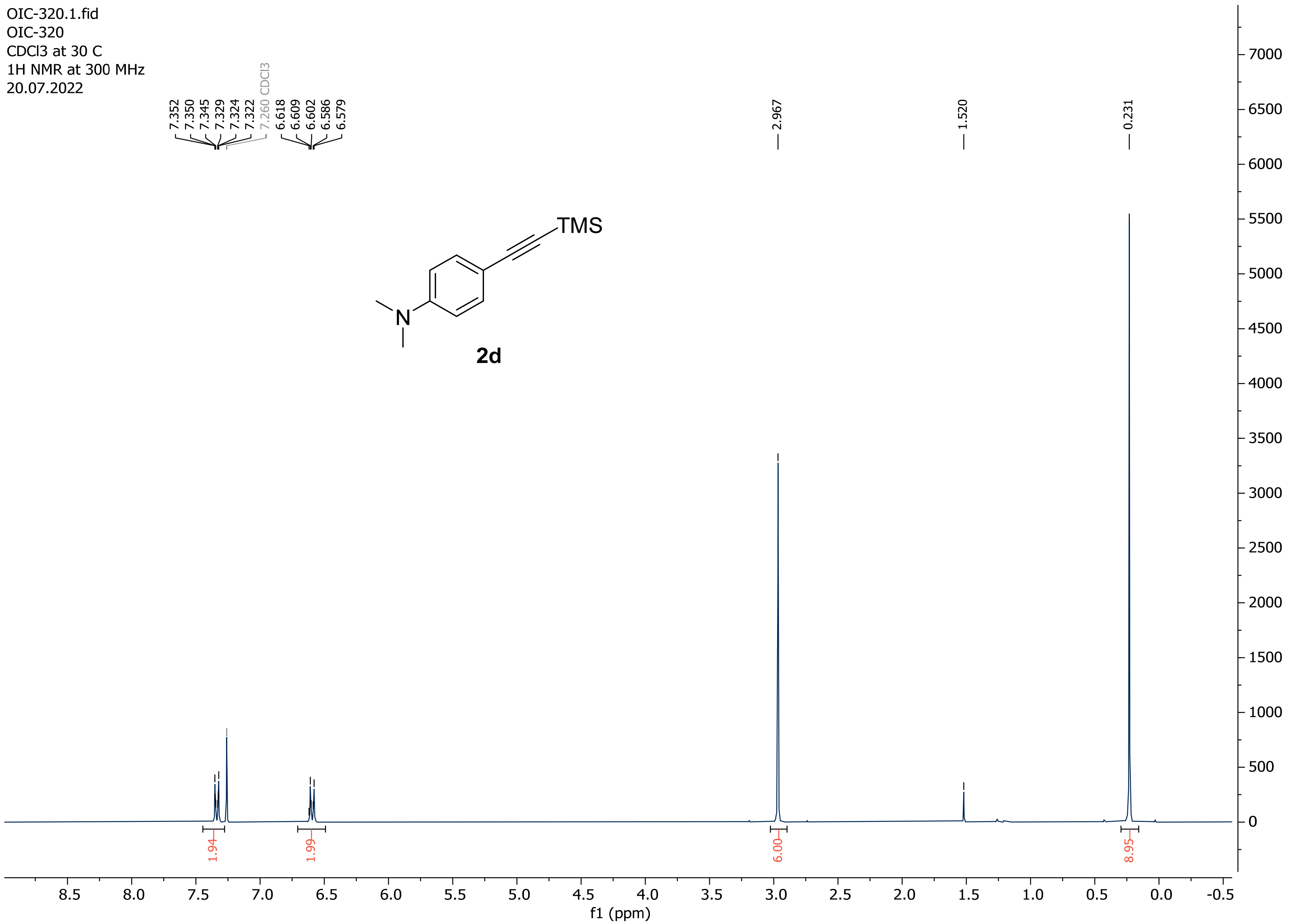

1.8 *N,N*-Dimethyl-4-((trimethylsilyl)ethynyl)aniline (**2d**), ^{13}C NMR spectrum

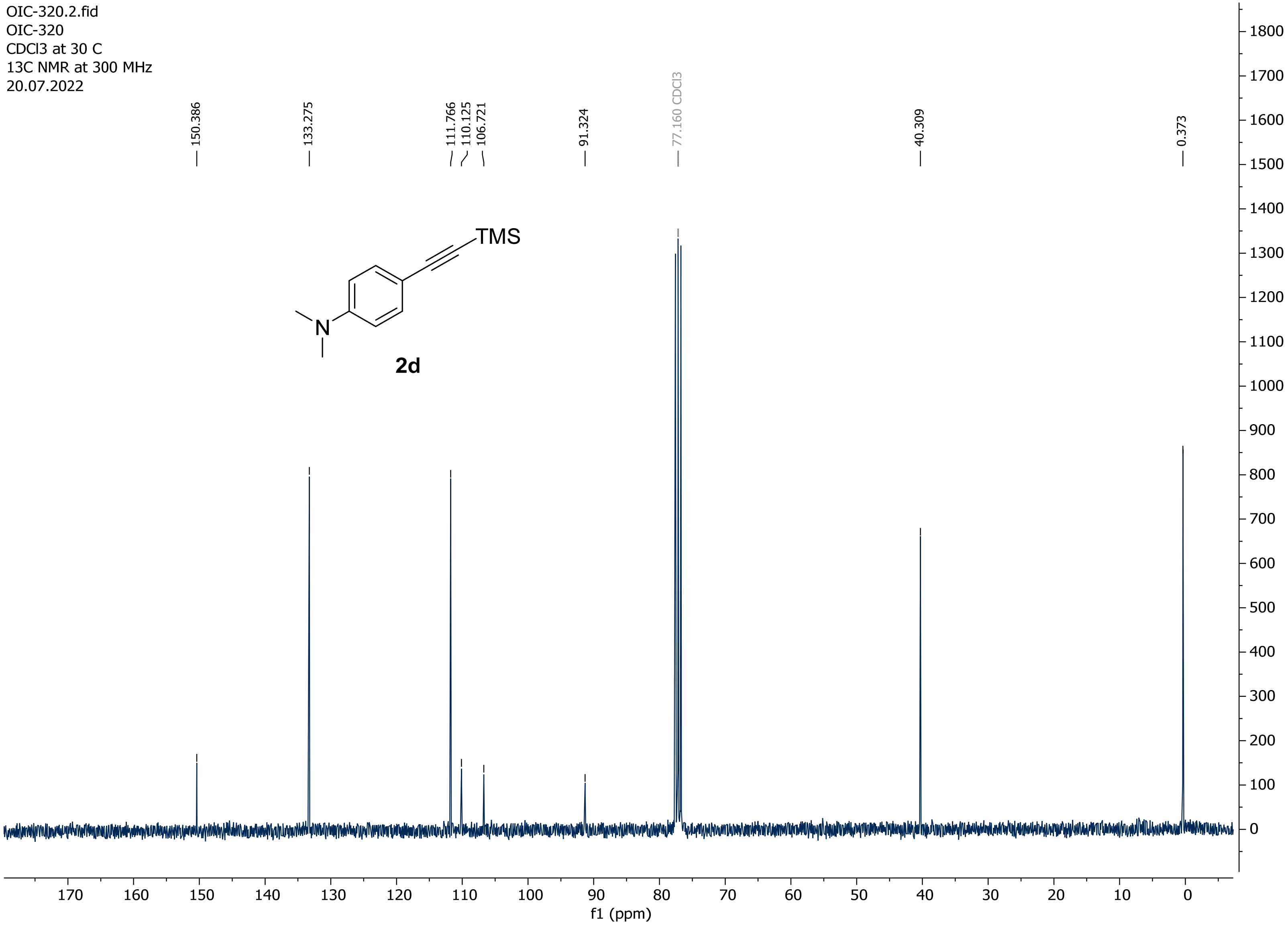

1.9 Trimethyl(*p*-tolylethynyl)silane (**2e**), ^{1}H NMR spectrum

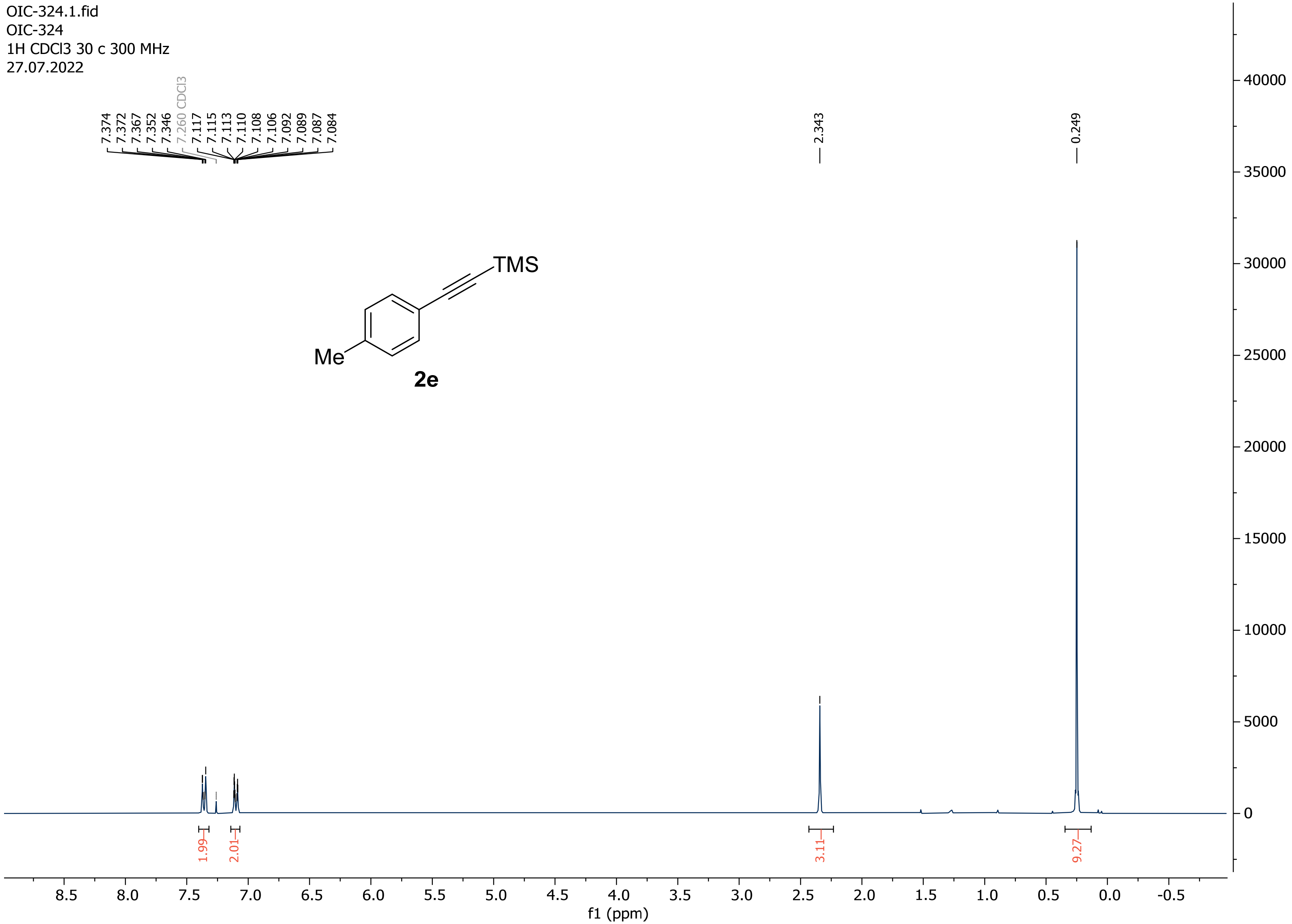

1.10 Trimethyl(*p*-tolylethynyl)silane (**2e**), ^{13}C NMR spectrum

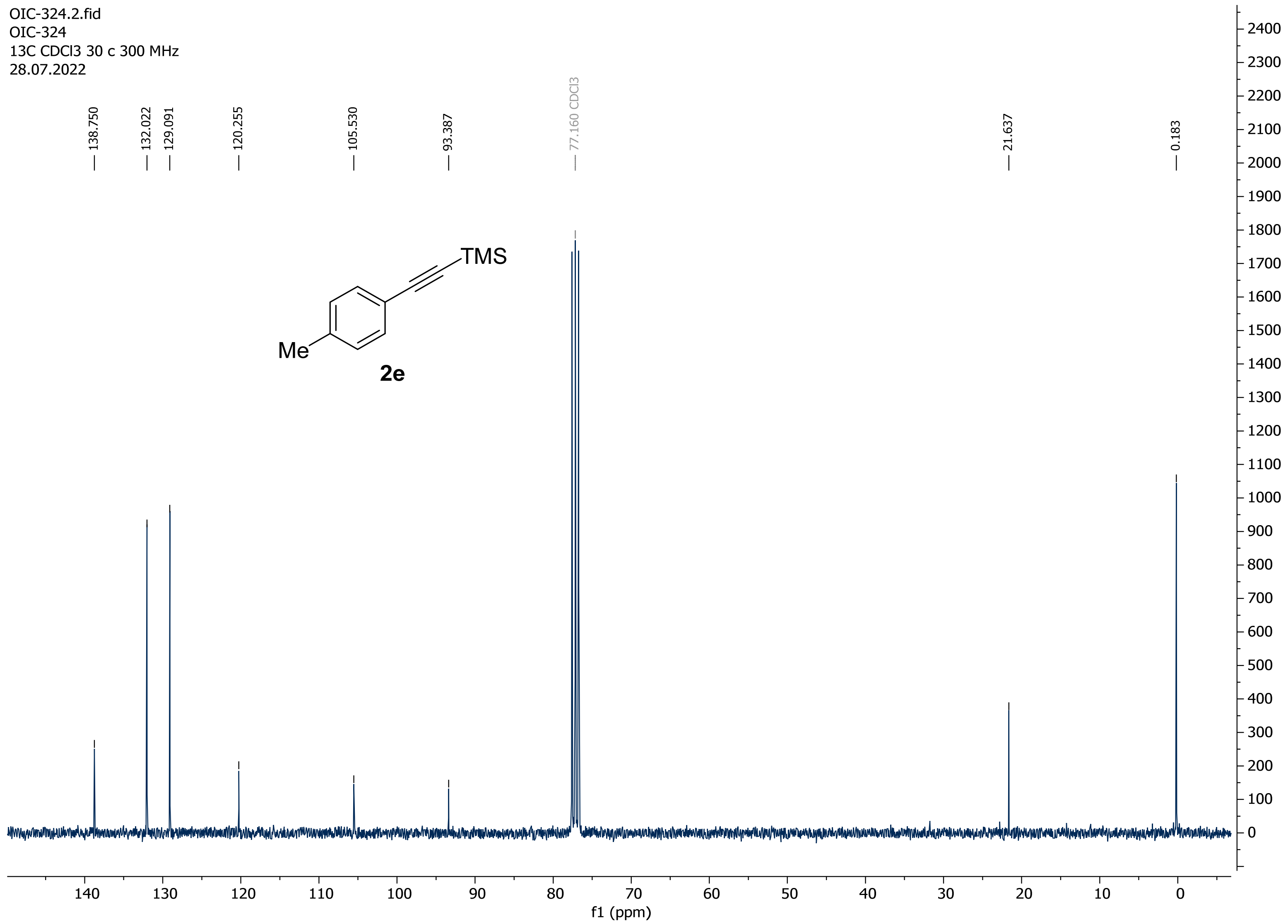

1.11 ((4-Fluorophenyl)ethynyl)trimethylsilane (**2f**), ^{1}H NMR spectrum

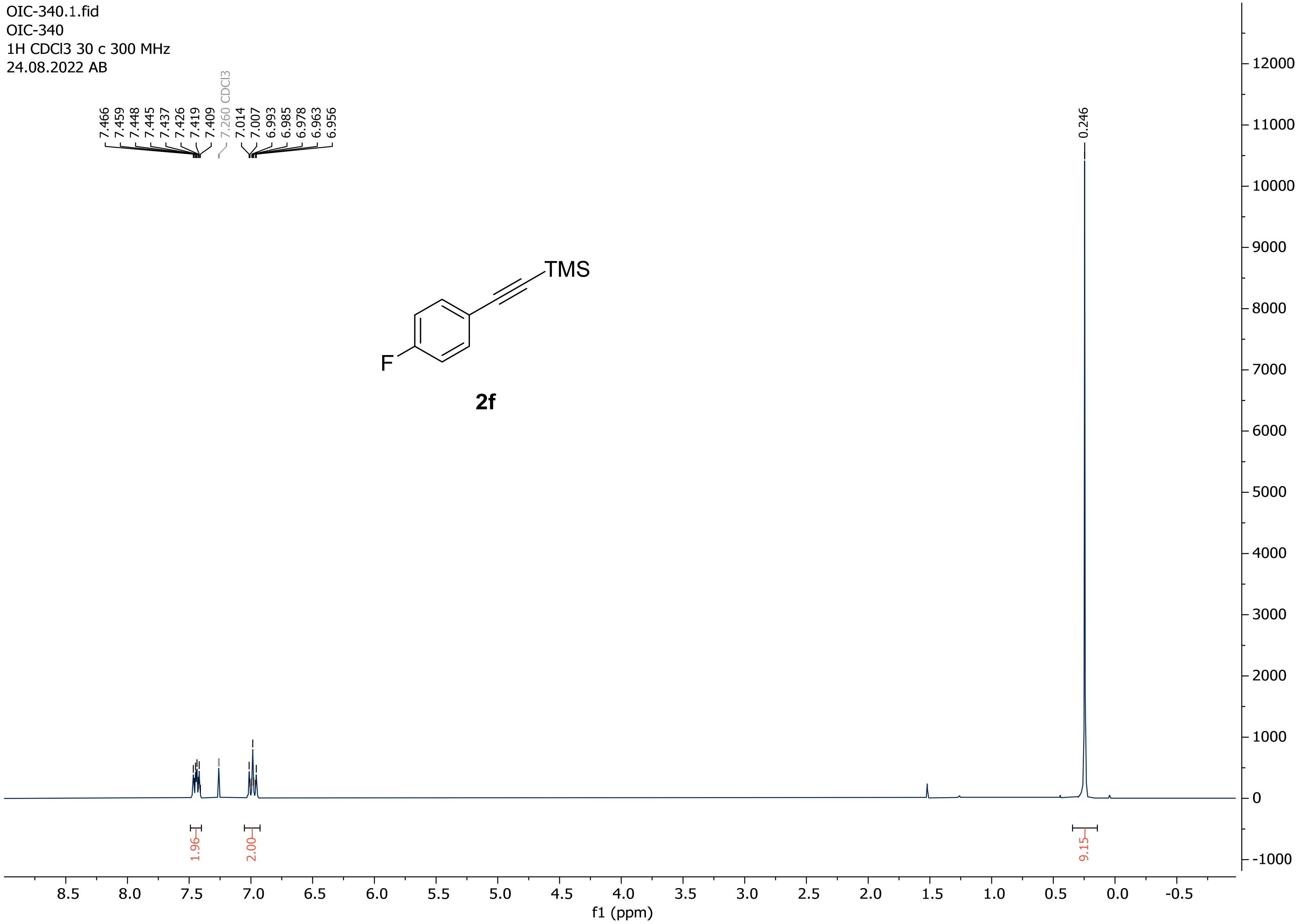

1.12 ((4-Fluorophenyl)ethynyl)trimethylsilane (**2f**), ^{13}C NMR spectrum

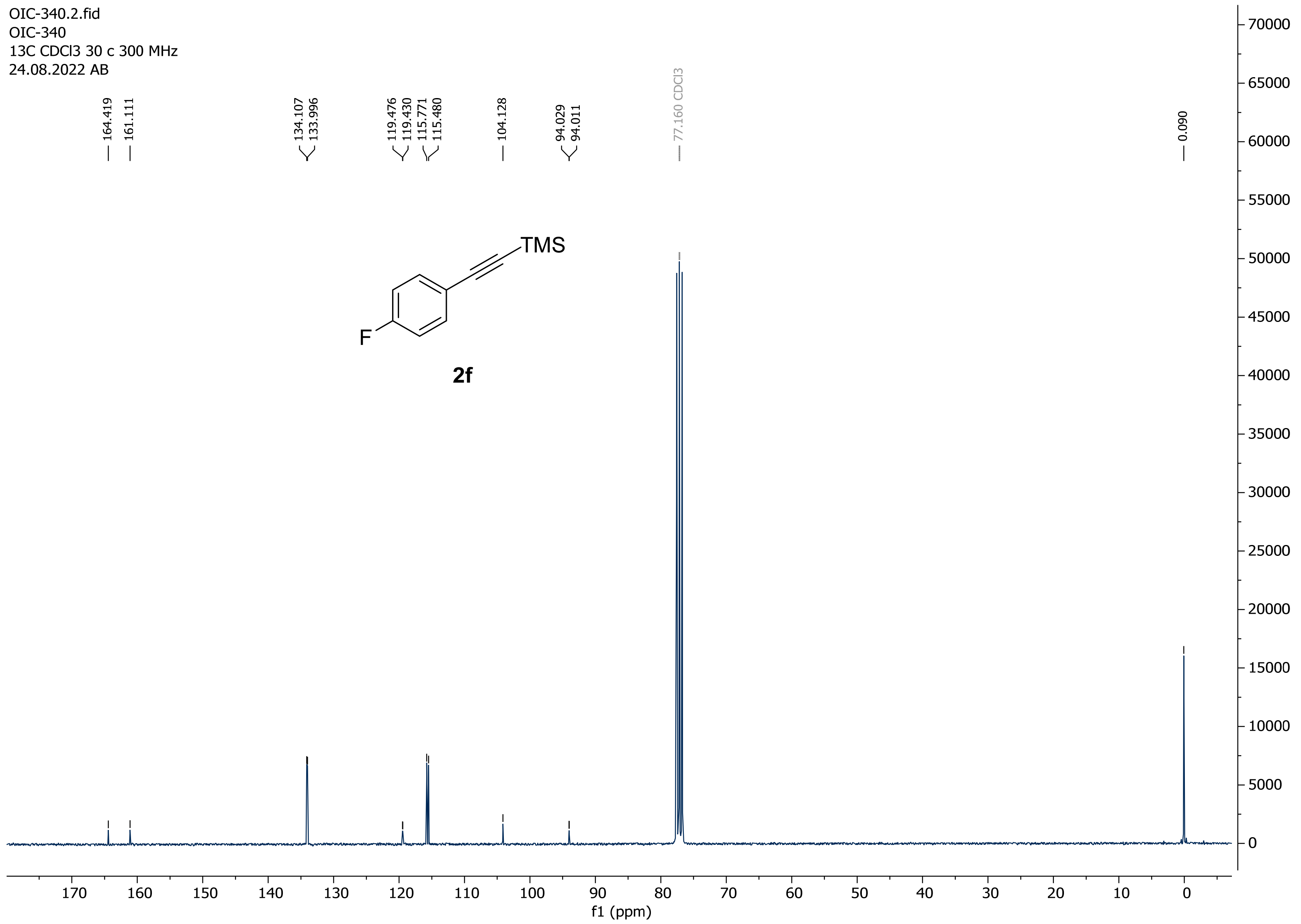

1.13 ((4-chlorophenyl)ethynyl)trimethylsilane (**2g**), ^{1}H NMR spectrum

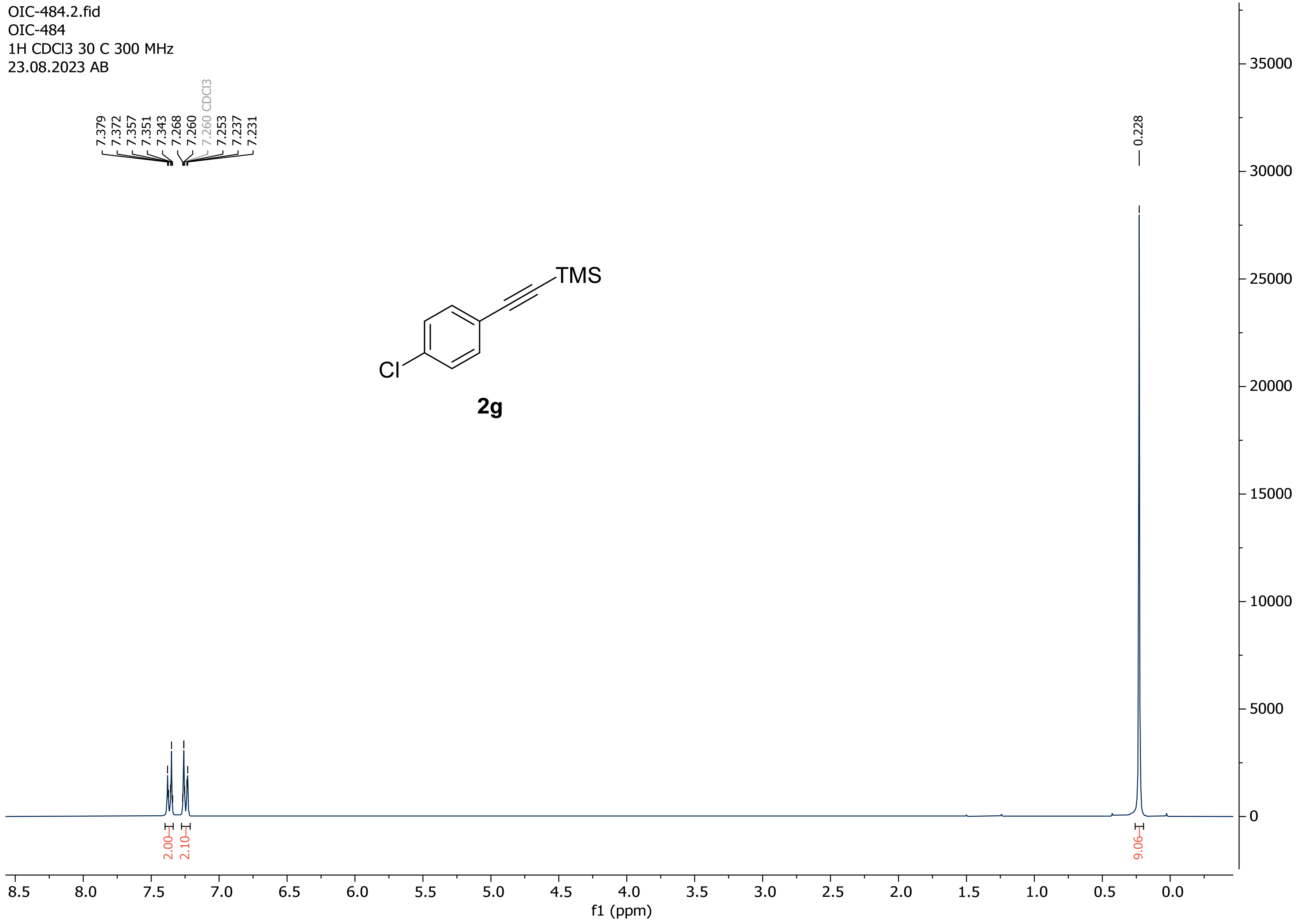

1.14 ((4-chlorophenyl)ethynyl)trimethylsilane (**2g**), ^{13}C NMR spectrum

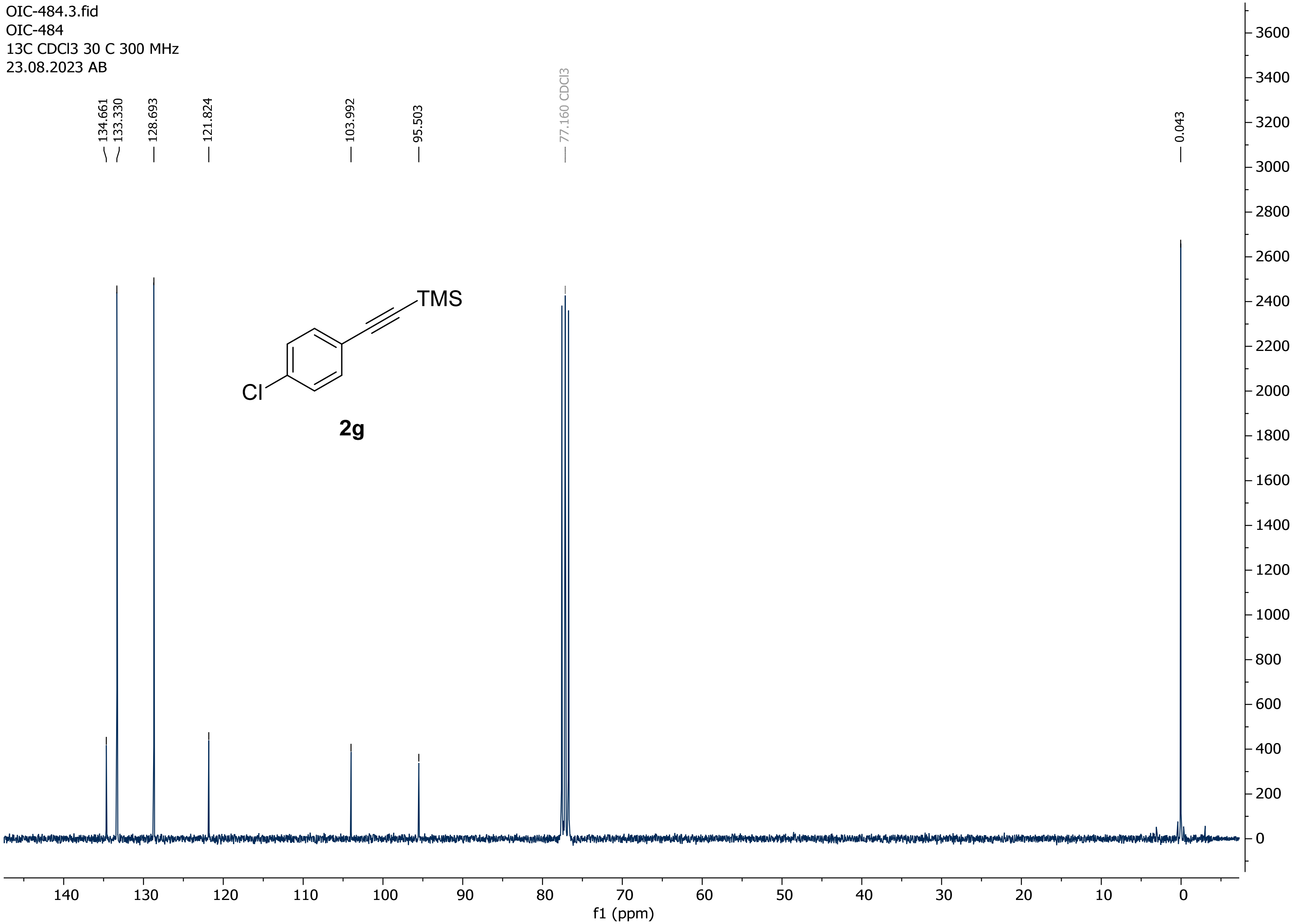

1.15 ((2-Chlorophenyl)ethynyl)trimethylsilane (**2h**), ^{1}H NMR spectrum

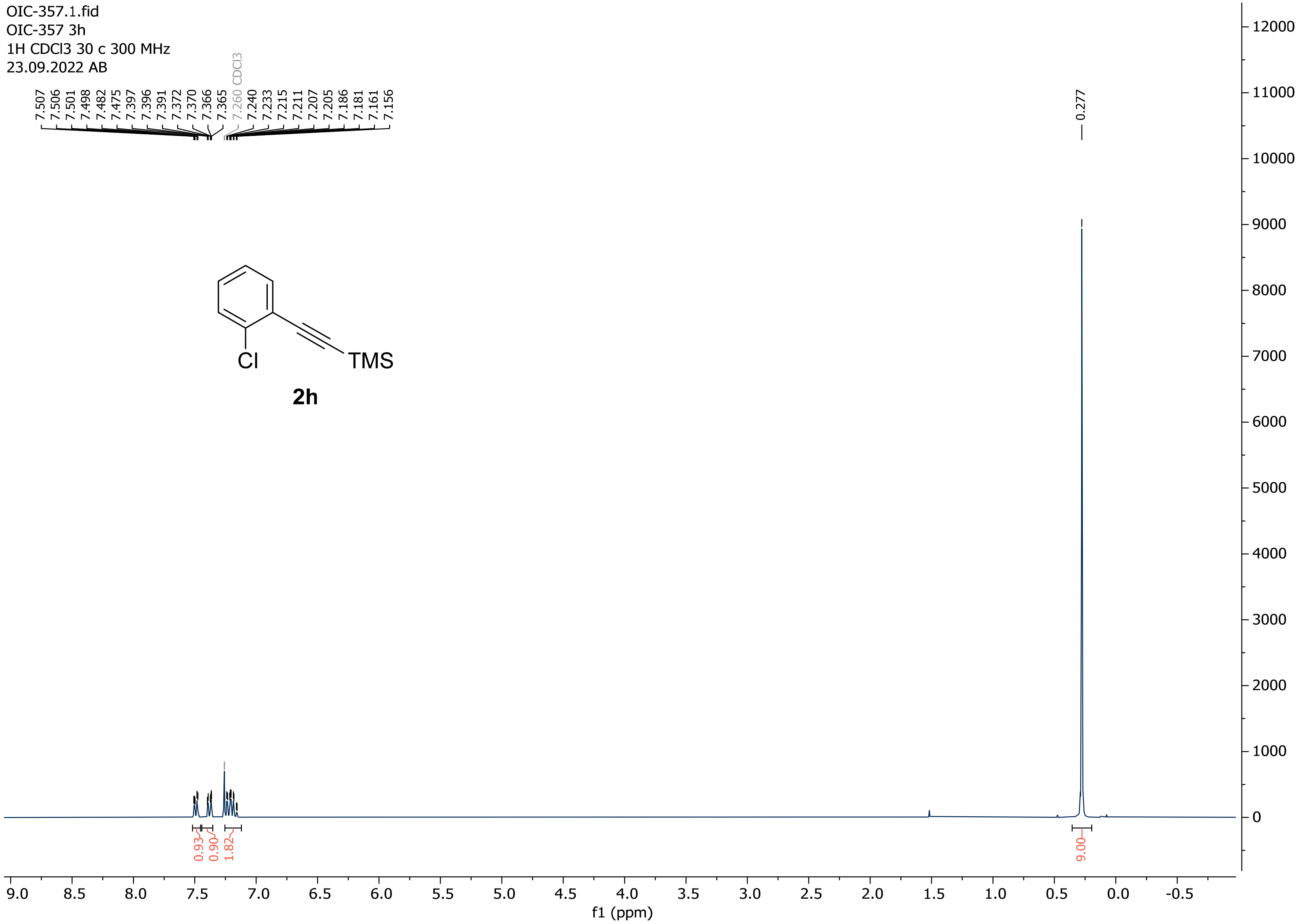

1.16 ((2-Chlorophenyl)ethynyl)trimethylsilane (**2h**), ^{13}C NMR spectrum

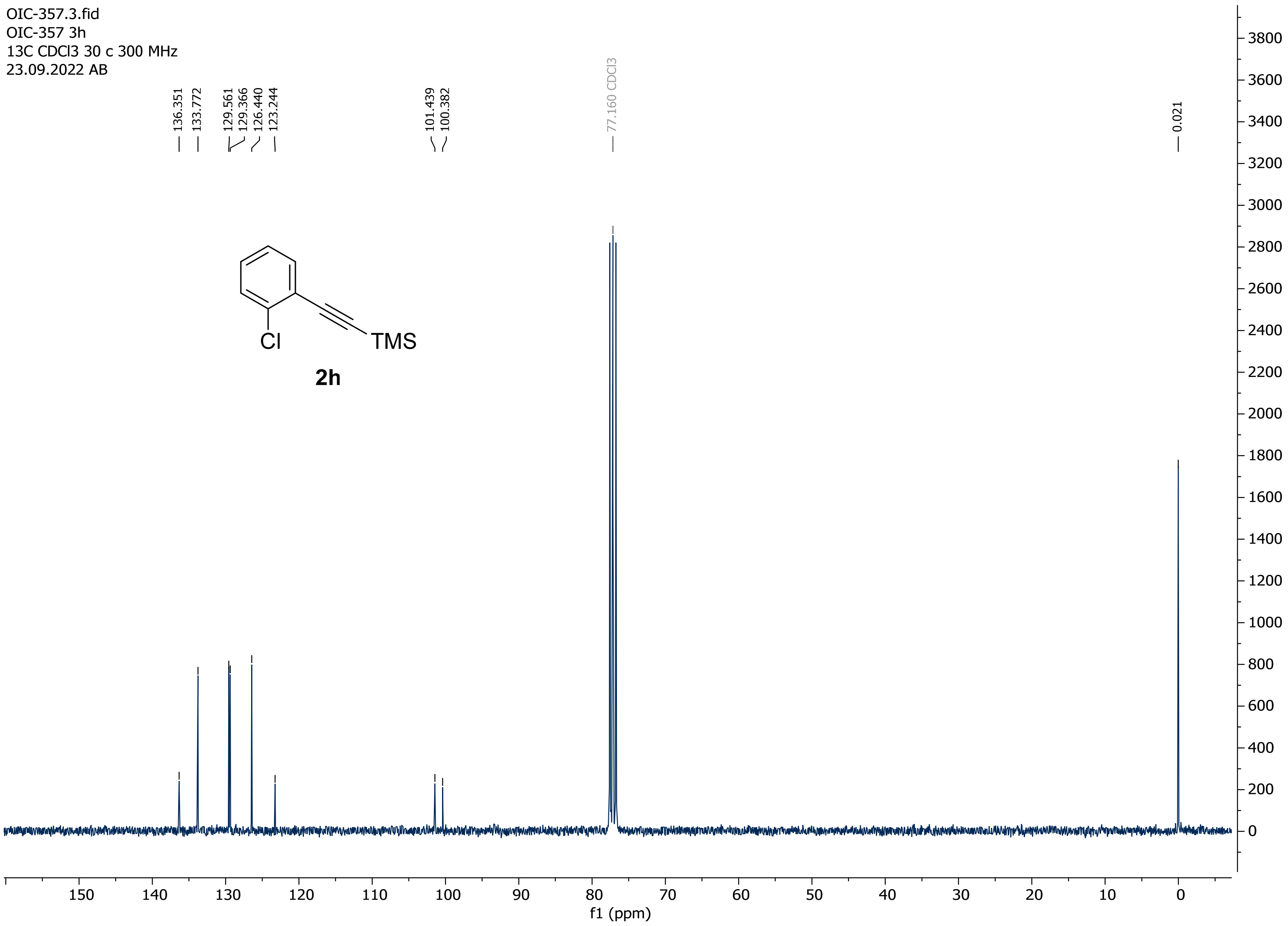

1.17 ((3,5-Bis(trifluoromethyl)phenyl)ethynyl)trimethylsilane (**2i**), ^{1}H NMR spectrum

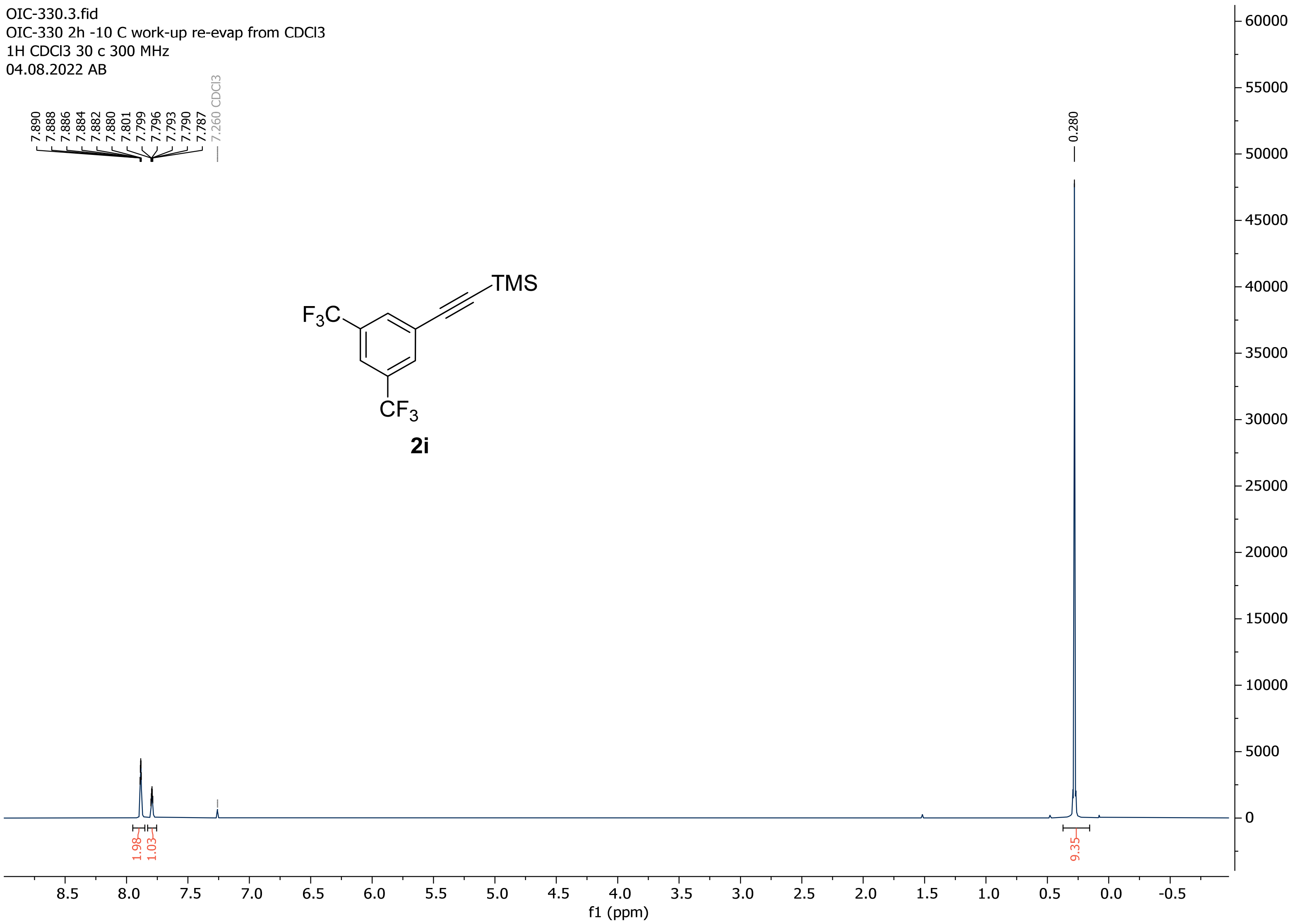

1.18 ((3,5-Bis(trifluoromethyl)phenyl)ethynyl)trimethylsilane (**2i**), ^{13}C NMR spectrum

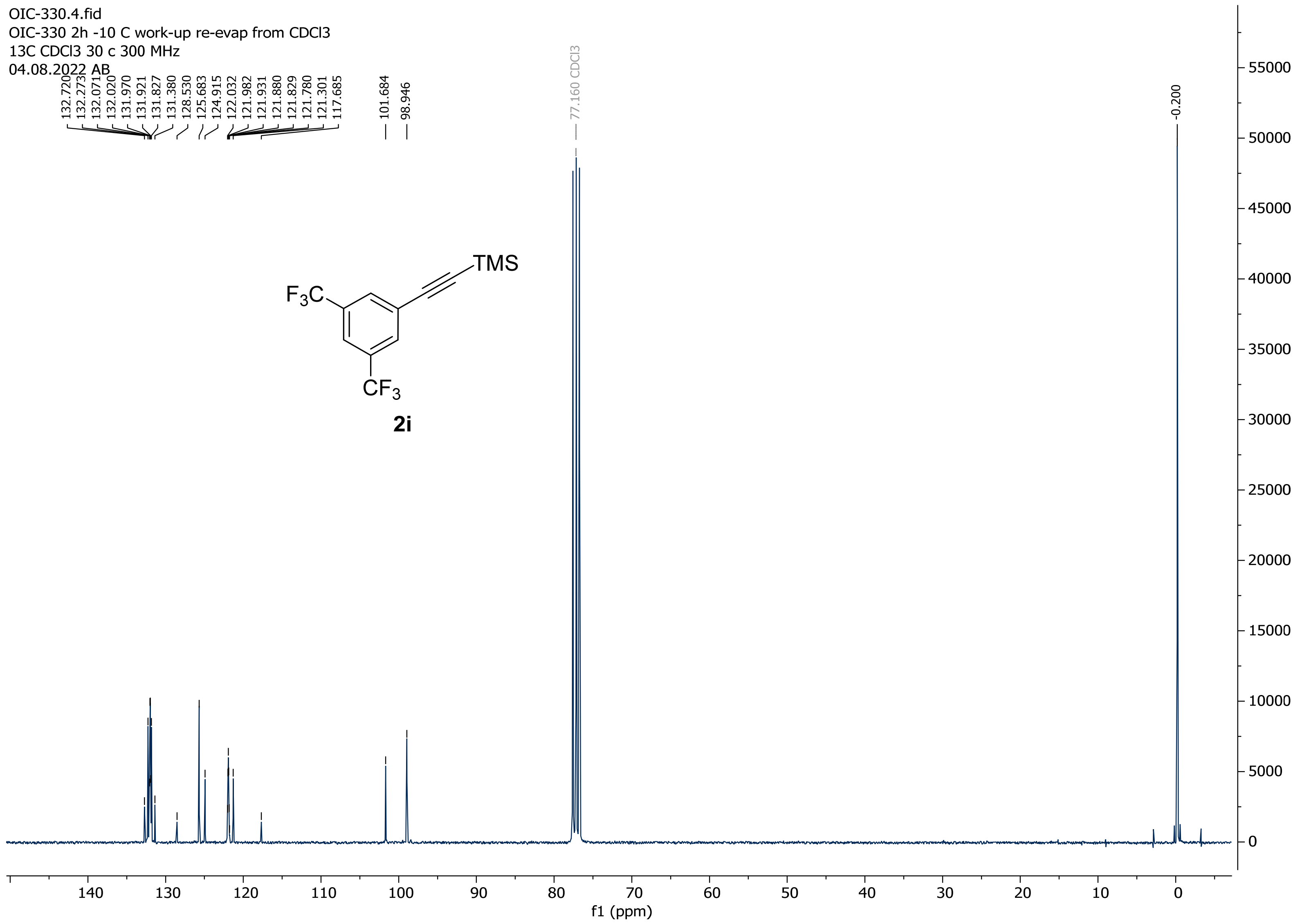

1.19 Ferrocenylethynetrimethylsilane (**2j**), ^{1}H NMR spectrum

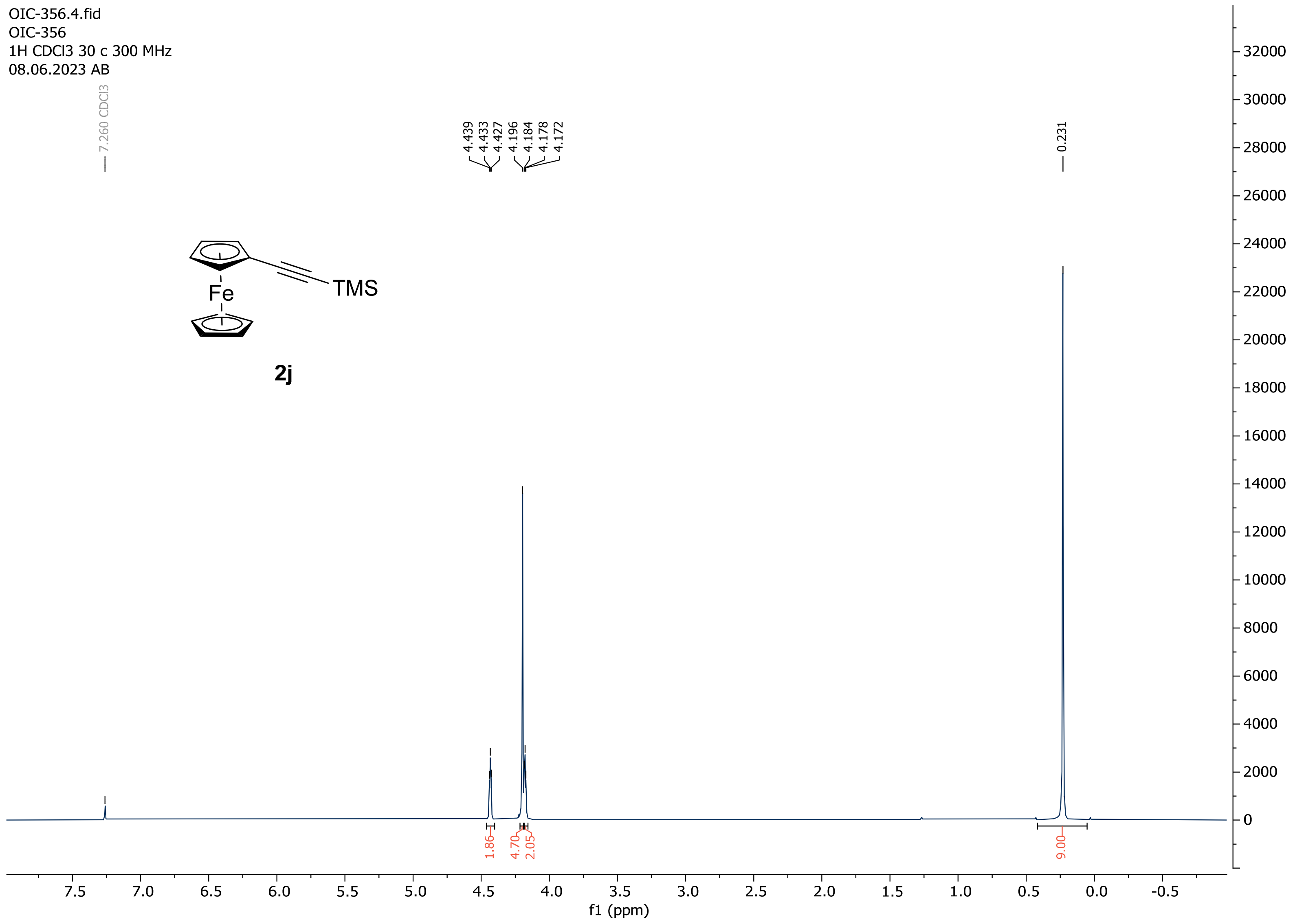

1.20 Ferrocenylethynetrimethylsilane (**2j**), ^{13}C NMR spectrum

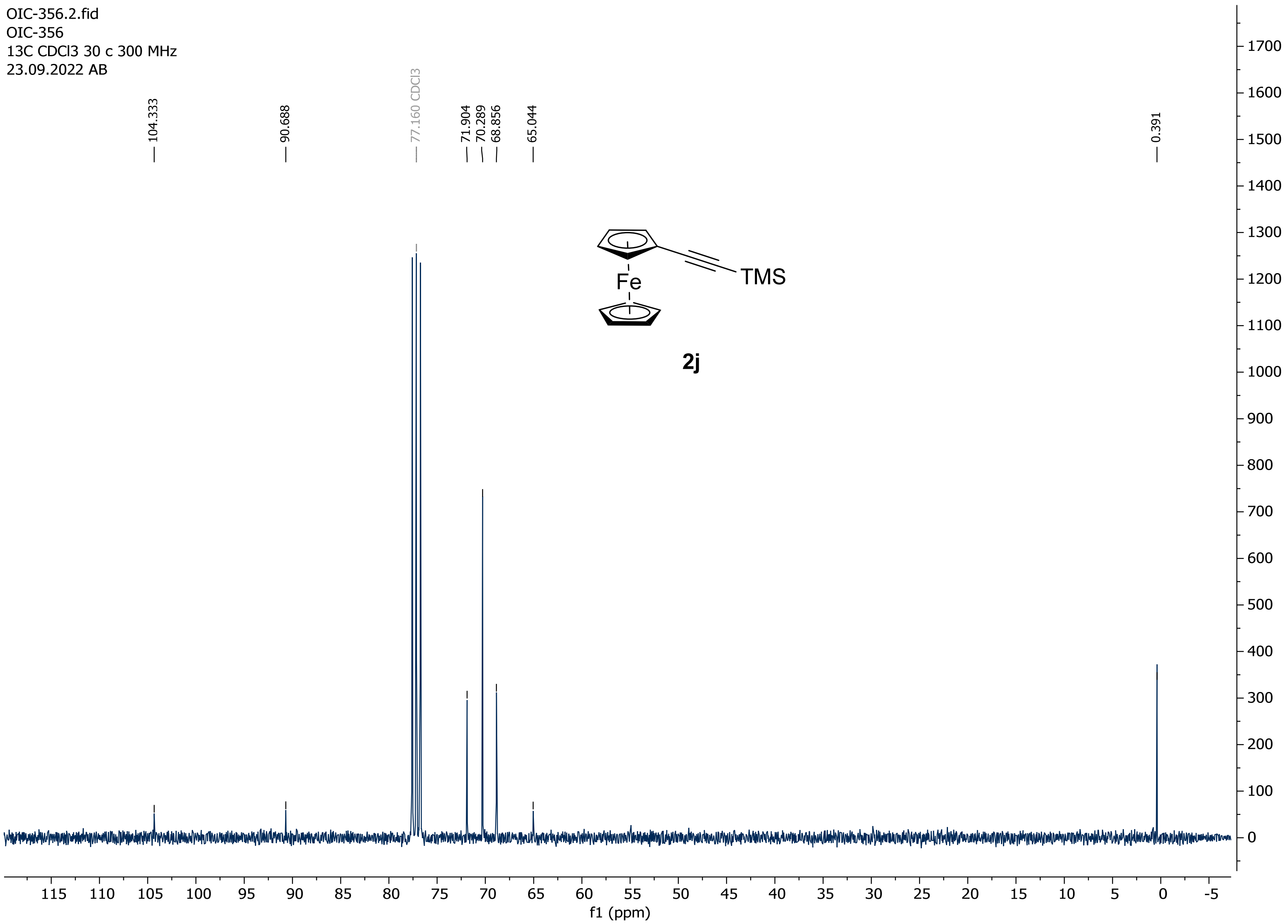

1.21 3-((Trimethylsilyl)ethynyl)pyridine (**2k**), ¹H NMR spectrum

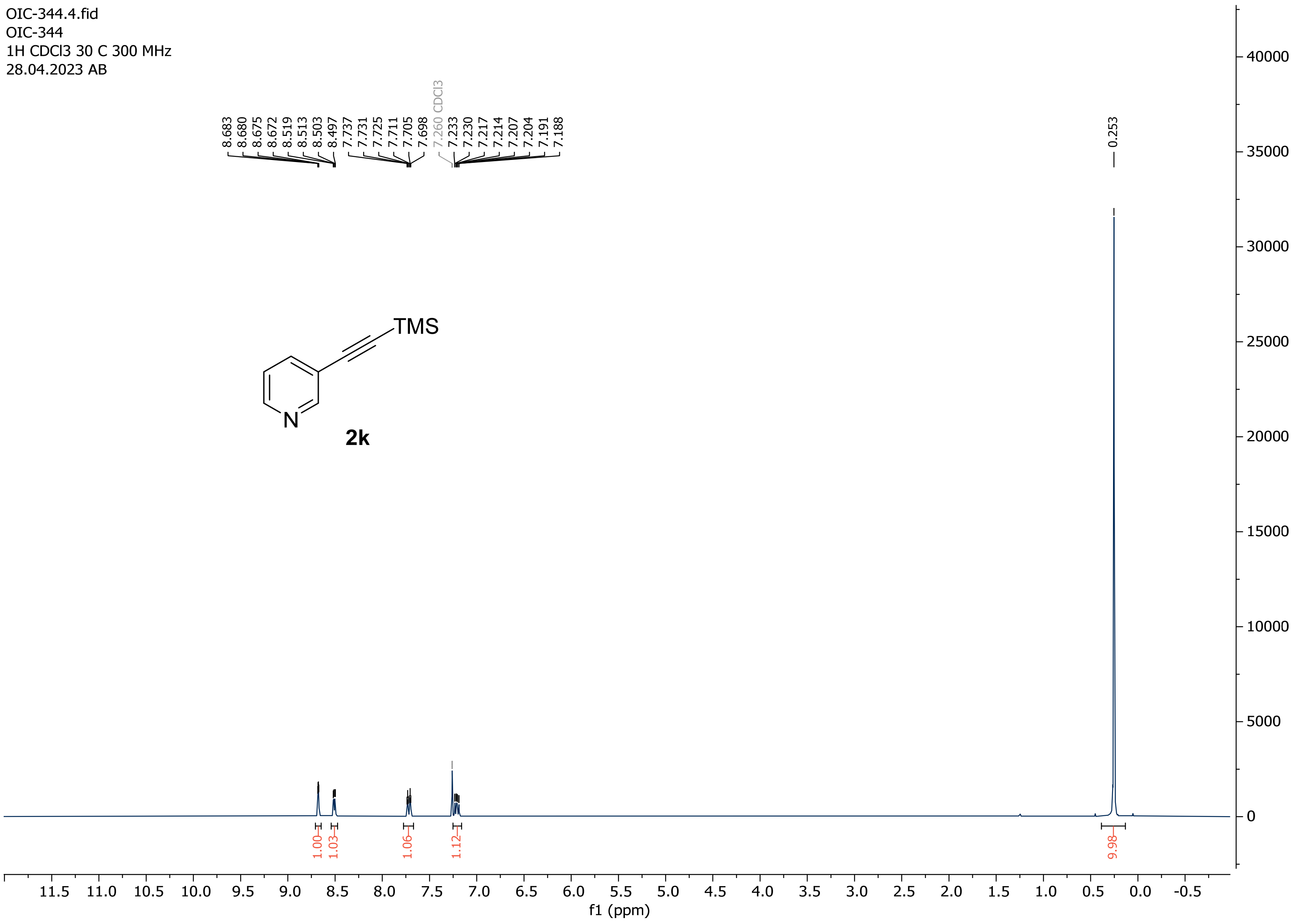

1.22 3-((Trimethylsilyl)ethynyl)pyridine (**2k**), ^{13}C NMR spectrum

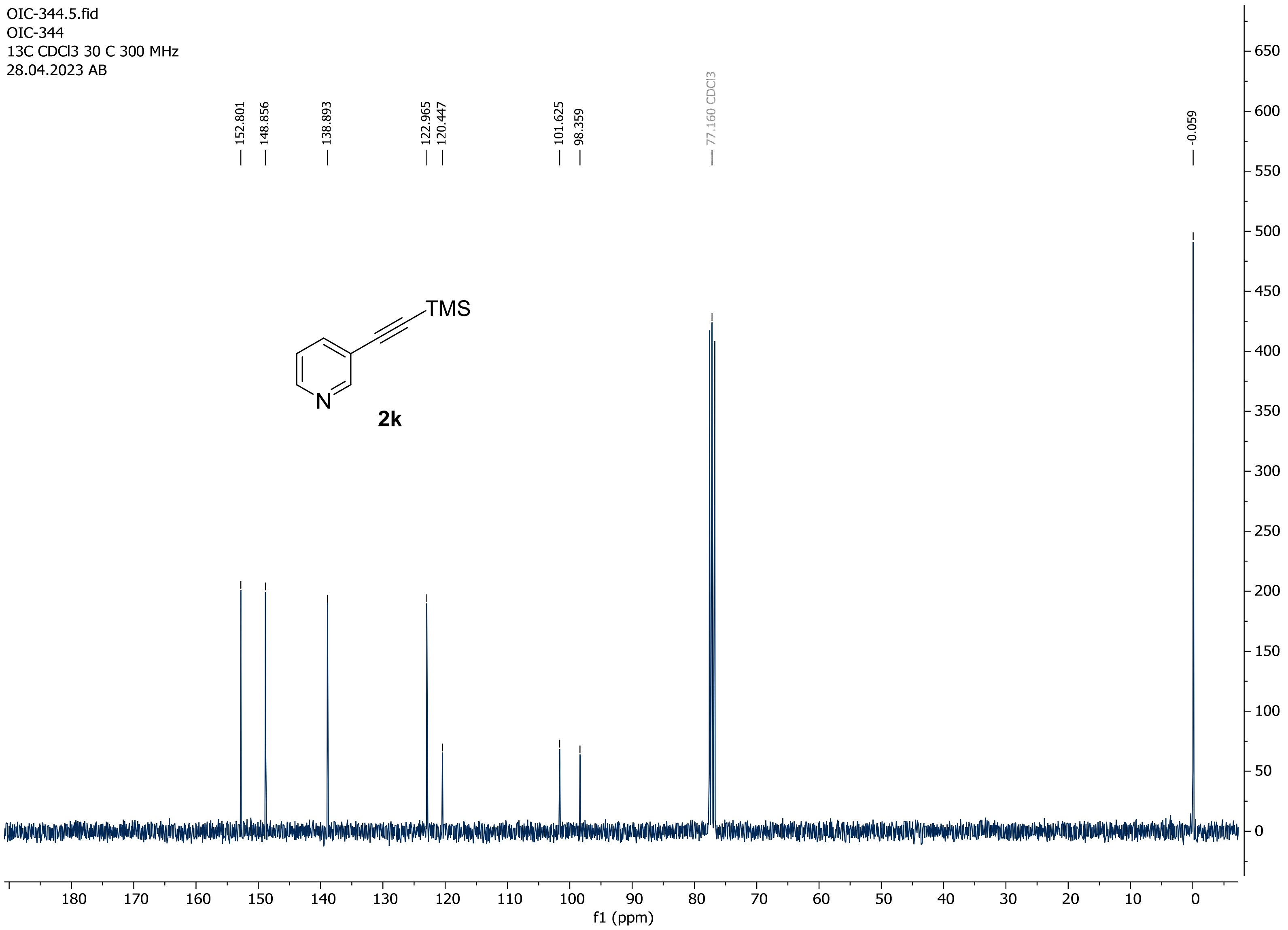

1.23 Trimethyl(thiophen-3-ylethynyl)silane (**2l**), ^{1}H NMR spectrum

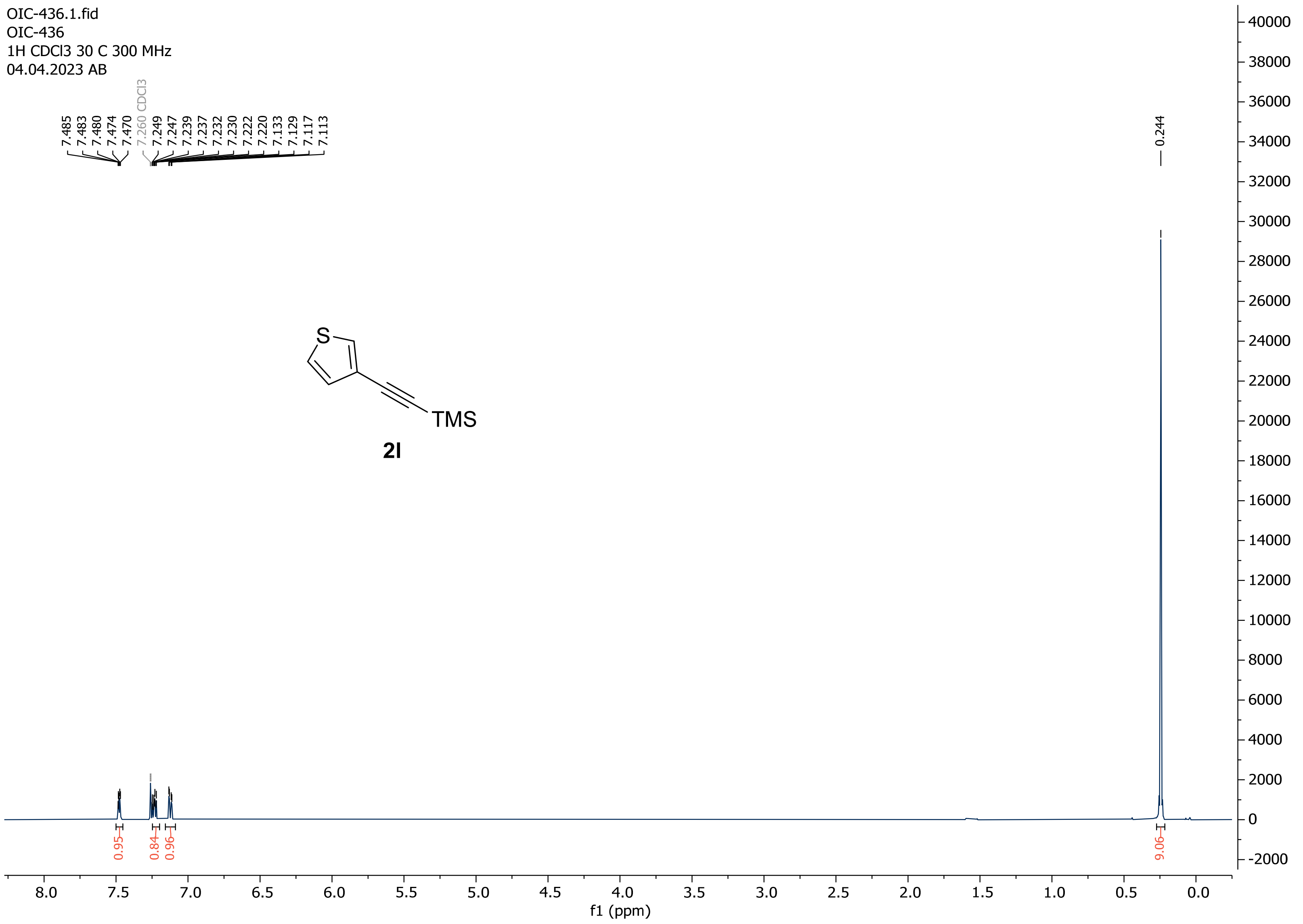

1.24 Trimethyl(thiophen-3-ylethynyl)silane (**2l**), ^{13}C NMR spectrum

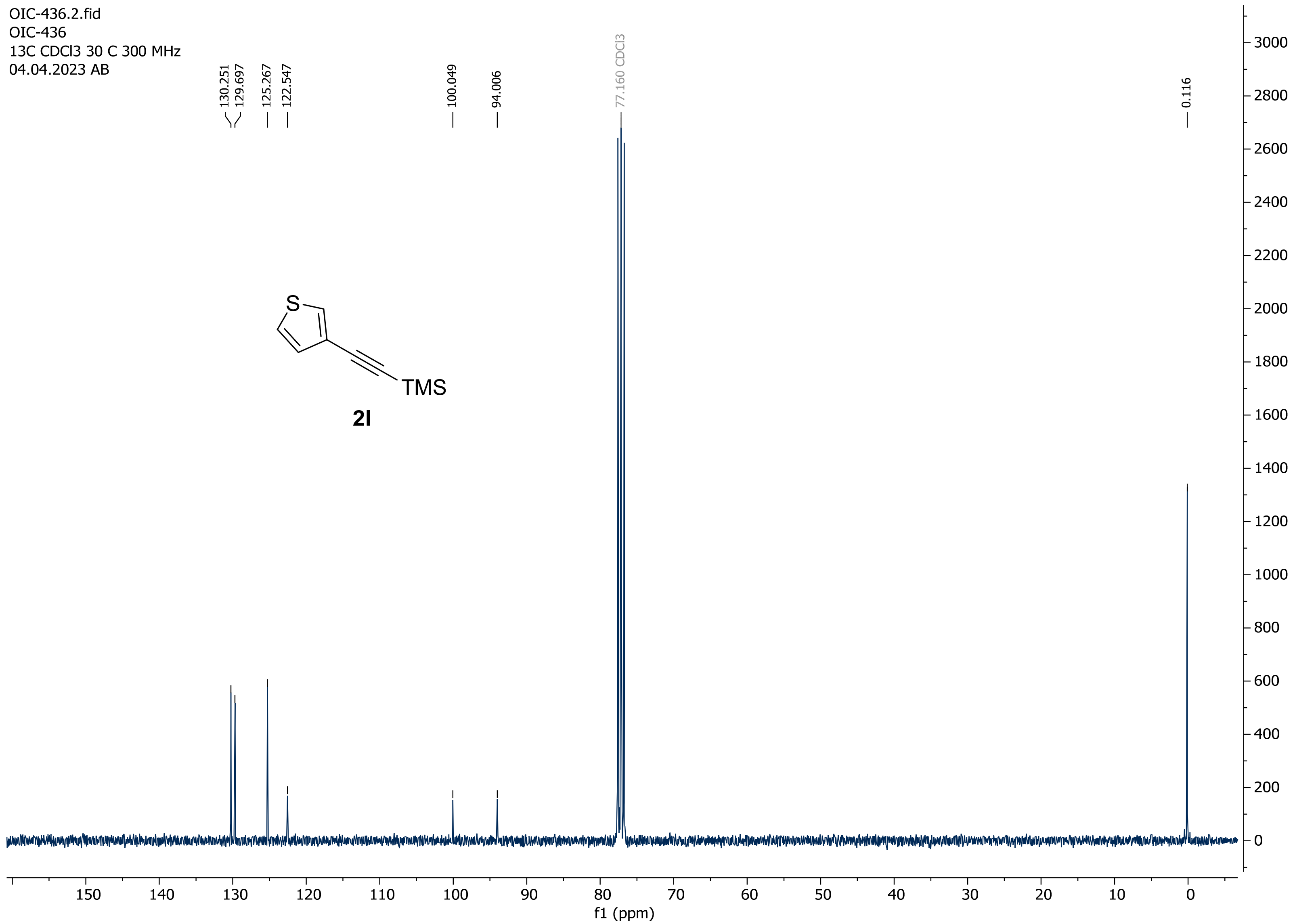

1.25 ((6-methoxynaphthalen-2-yl)ethynyl)trimethylsilane (**2m**), ^{1}H NMR spectrum

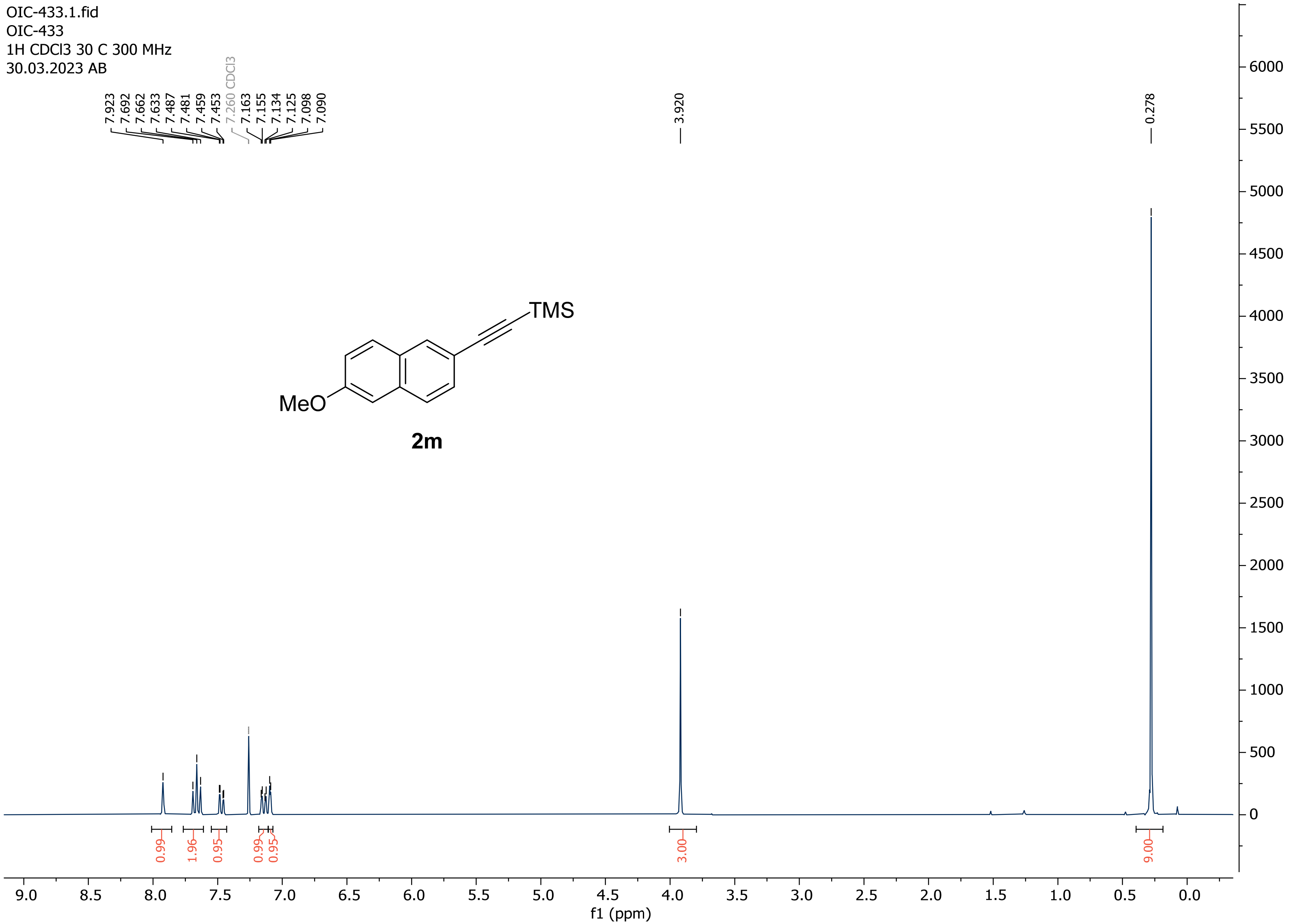

1.26 ((6-methoxynaphthalen-2-yl)ethynyl)trimethylsilane (**2m**), ^{13}C NMR spectrum

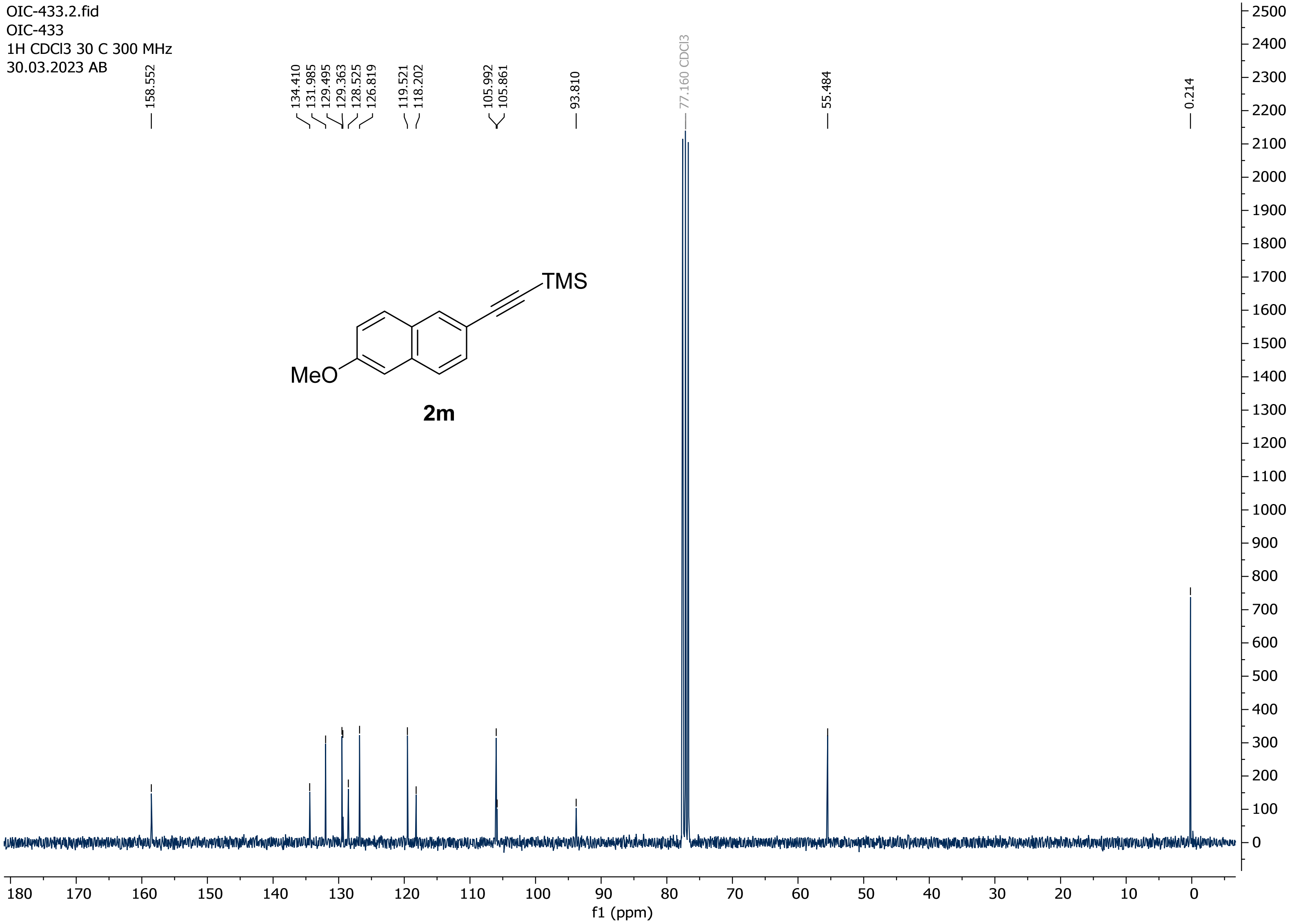

1.27 *tert*-Butyldimethyl((3-(trimethylsilyl)prop-2-yn-1-yl)oxy)silane (**2n**), ^{1}H NMR spectrum

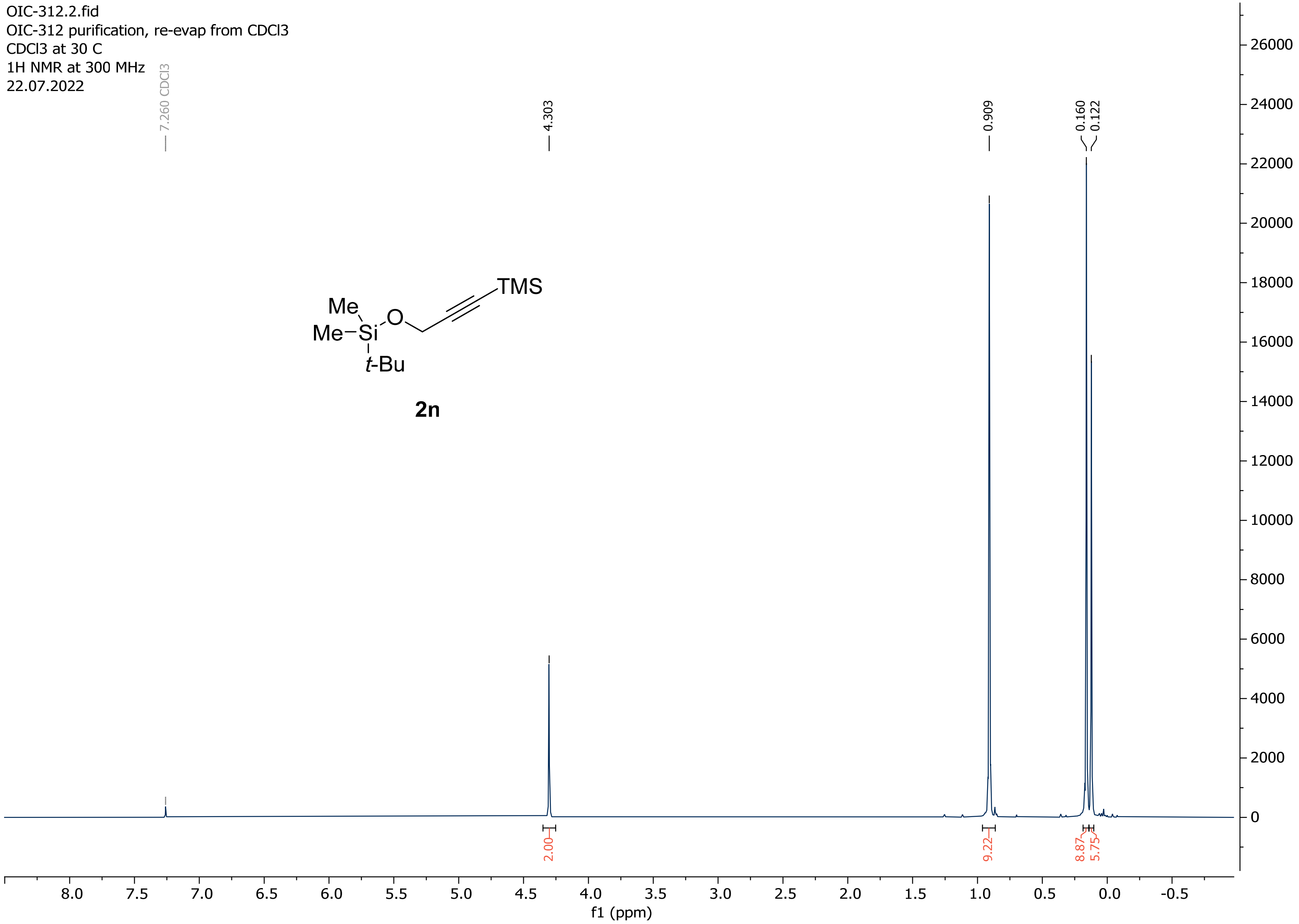

1.28 *tert*-Butyldimethyl((3-(trimethylsilyl)prop-2-yn-1-yl)oxy)silane (**2n**), ^{13}C NMR spectrum

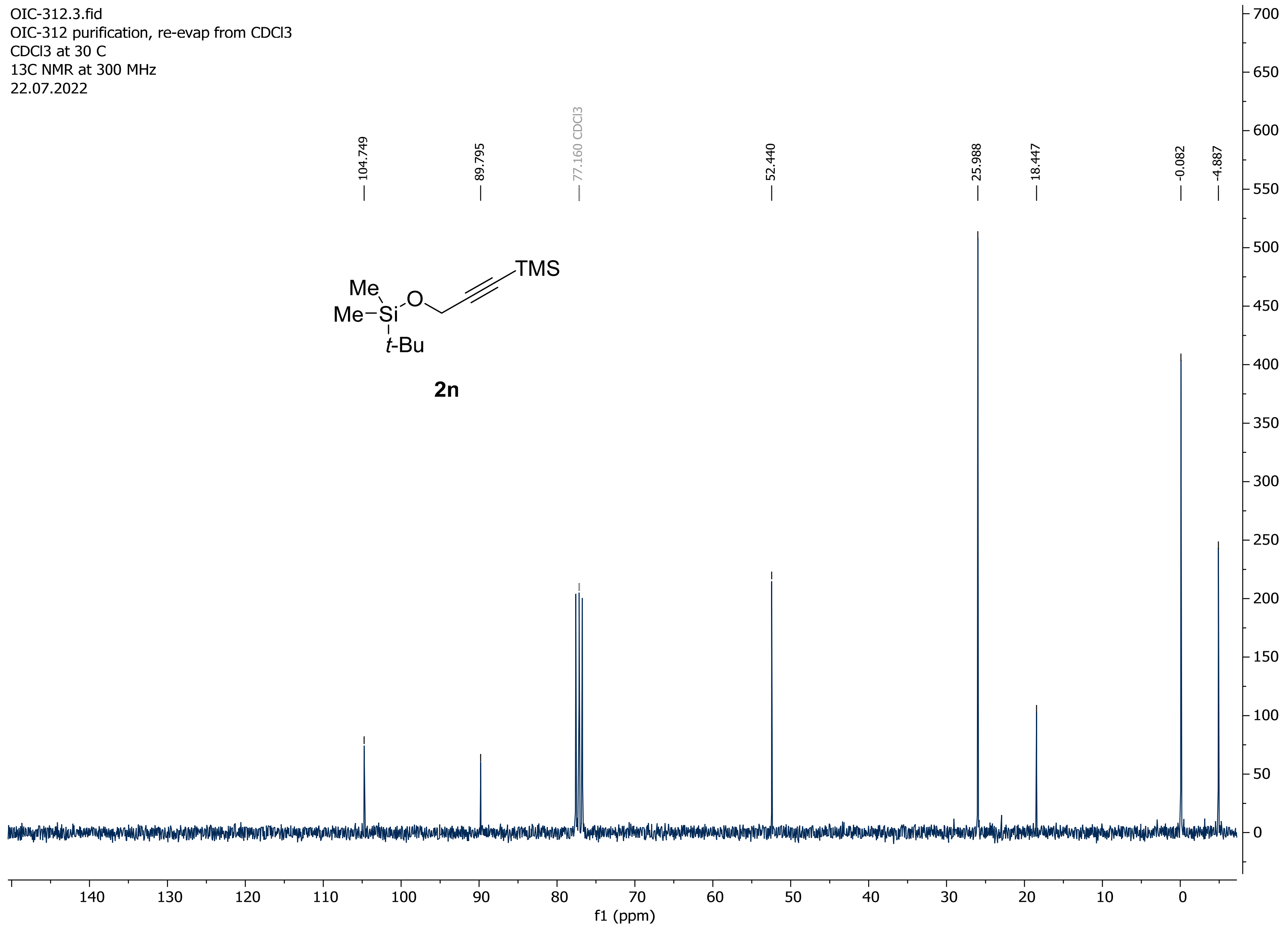

1.29 Trimethyl(3-(oxiran-2-ylmethoxy)prop-1-yn-1-yl)silane (**2o**), ^{1}H NMR spectrum

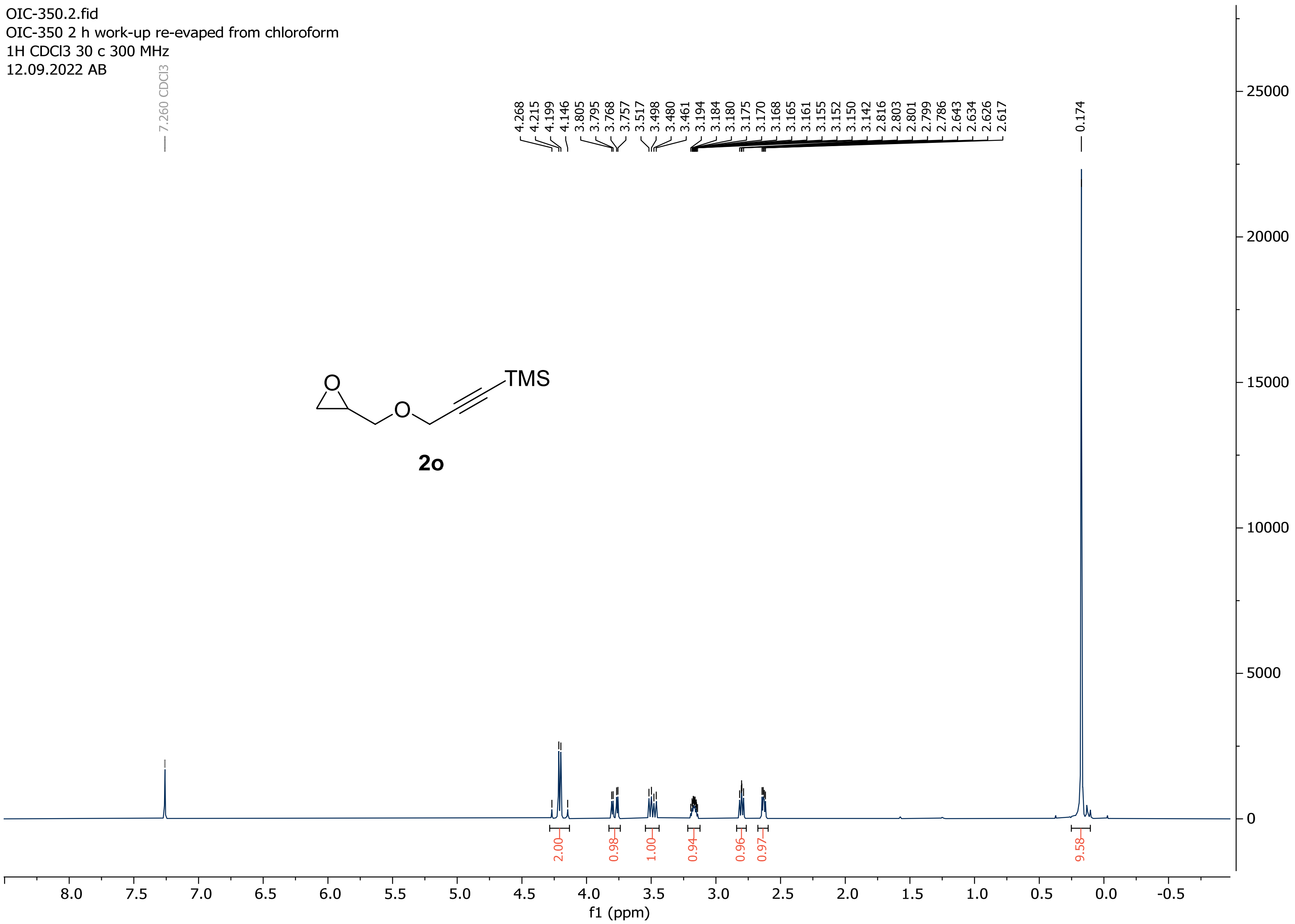

1.30 Trimethyl(3-(oxiran-2-ylmethoxy)prop-1-yn-1-yl)silane (**2o**), ^{13}C NMR spectrum

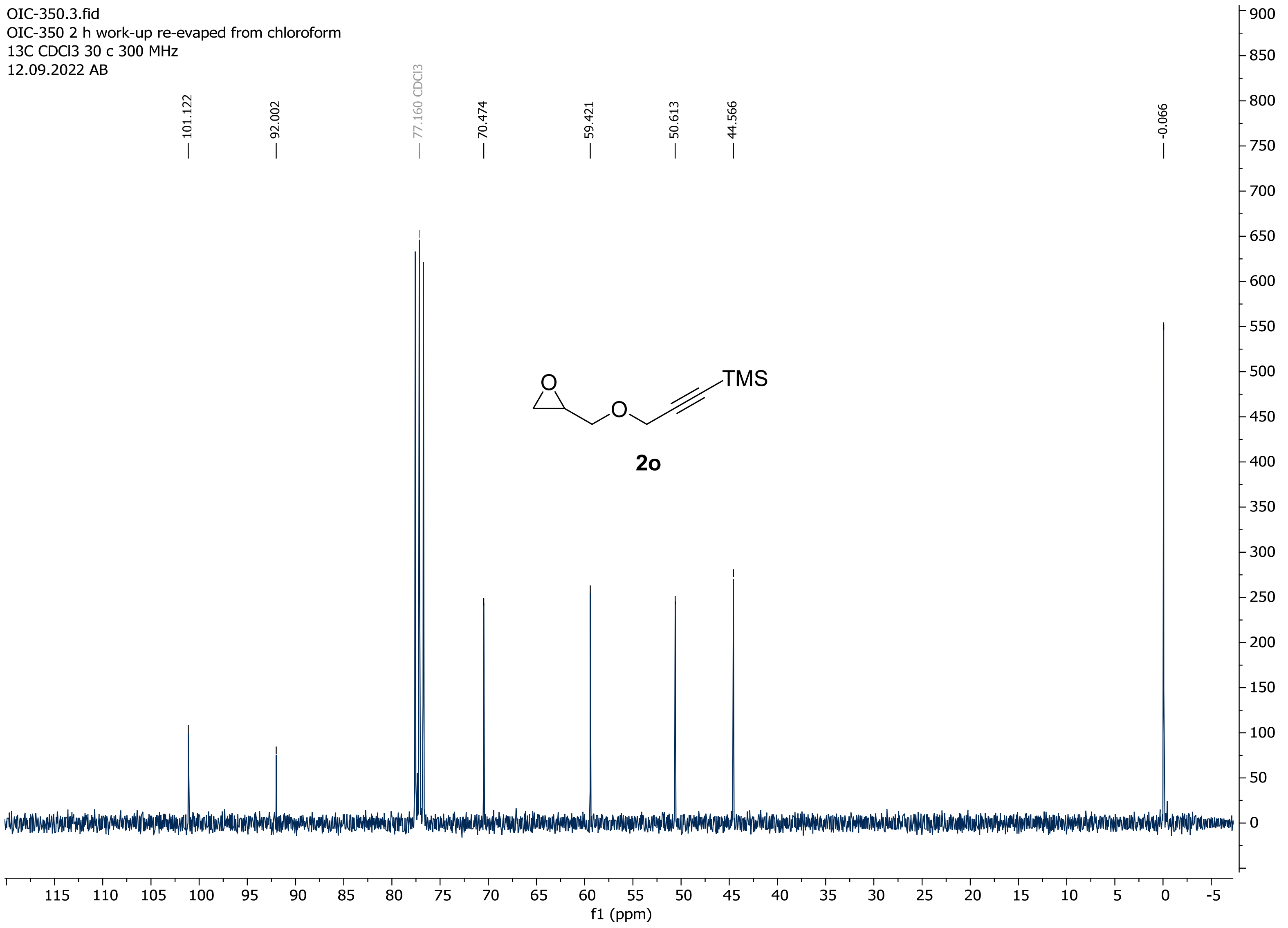

1.31 1-(3-(trimethylsilyl)prop-2-yn-1-yl)-1H-indole (**2p**), ^{1}H NMR spectrum

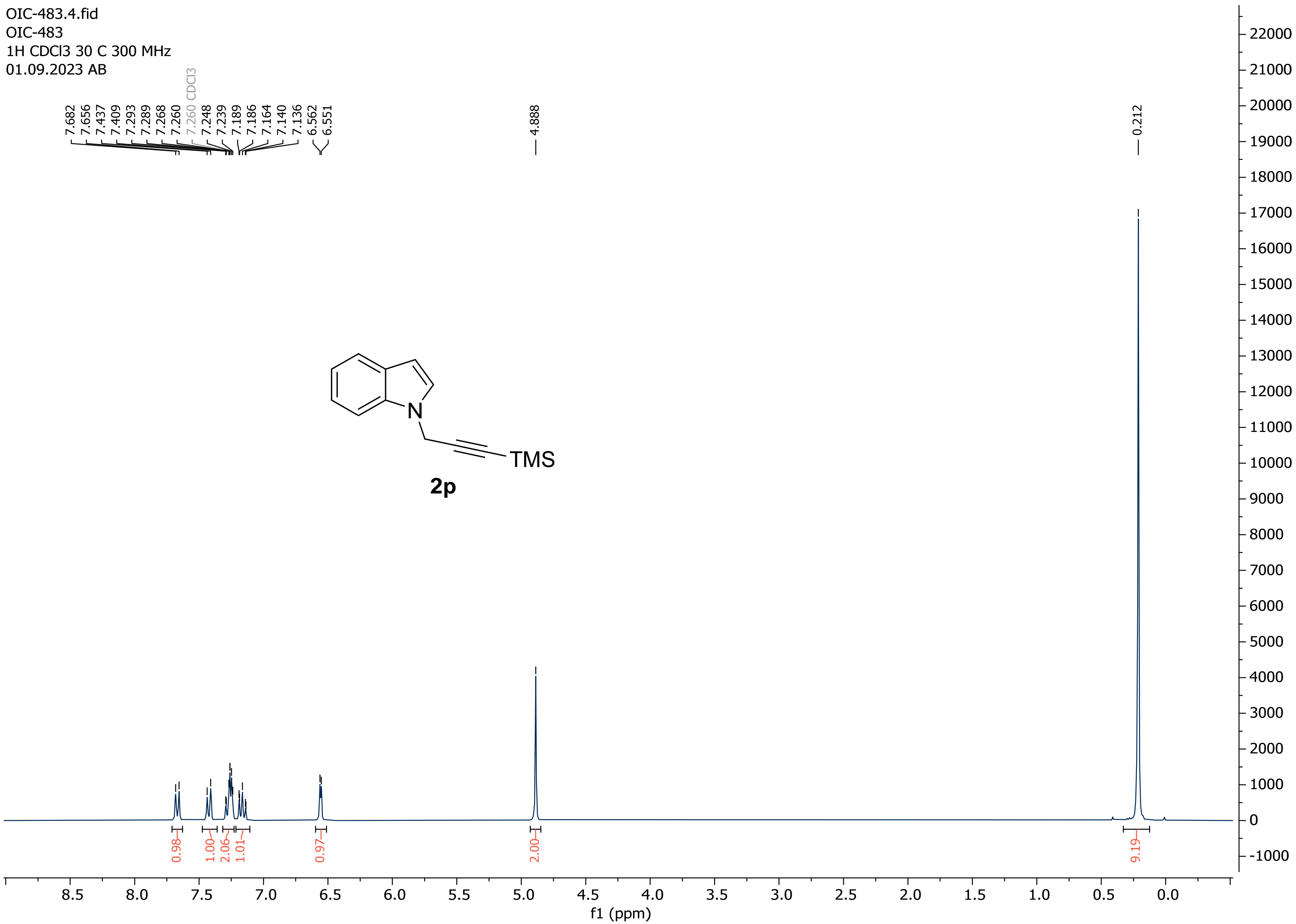

1.32 *1-(3-(trimethylsilyl)prop-2-yn-1-yl)-1H-indole* (**2p**), ^{13}C NMR spectrum

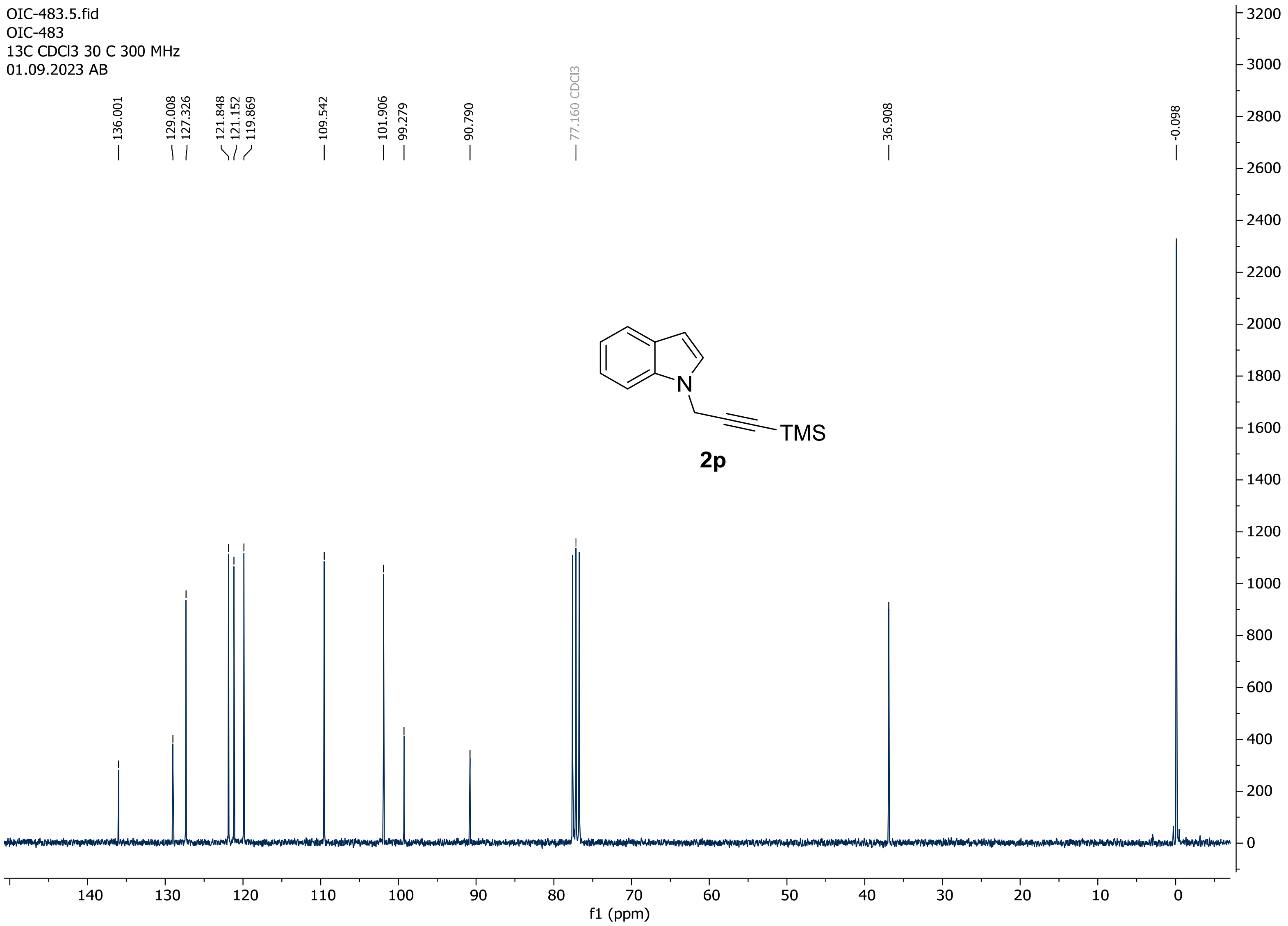

1.33 (Cyclohex-1-en-1-ylethynyl)trimethylsilane (**2q**), ^{1}H NMR spectrum

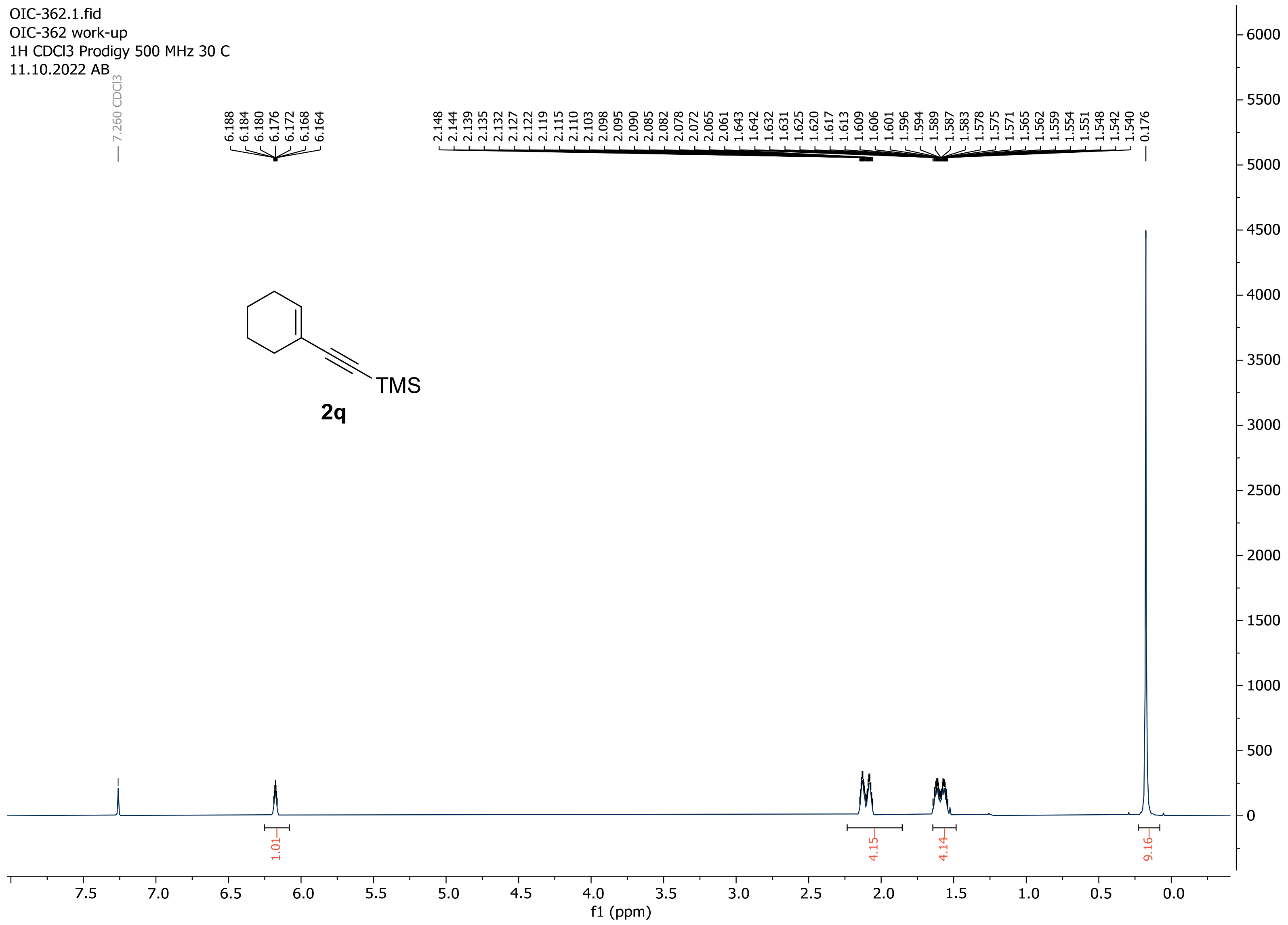

1.34 (Cyclohex-1-en-1-ylethynyl)trimethylsilane (**2q**), ^{13}C NMR spectrum

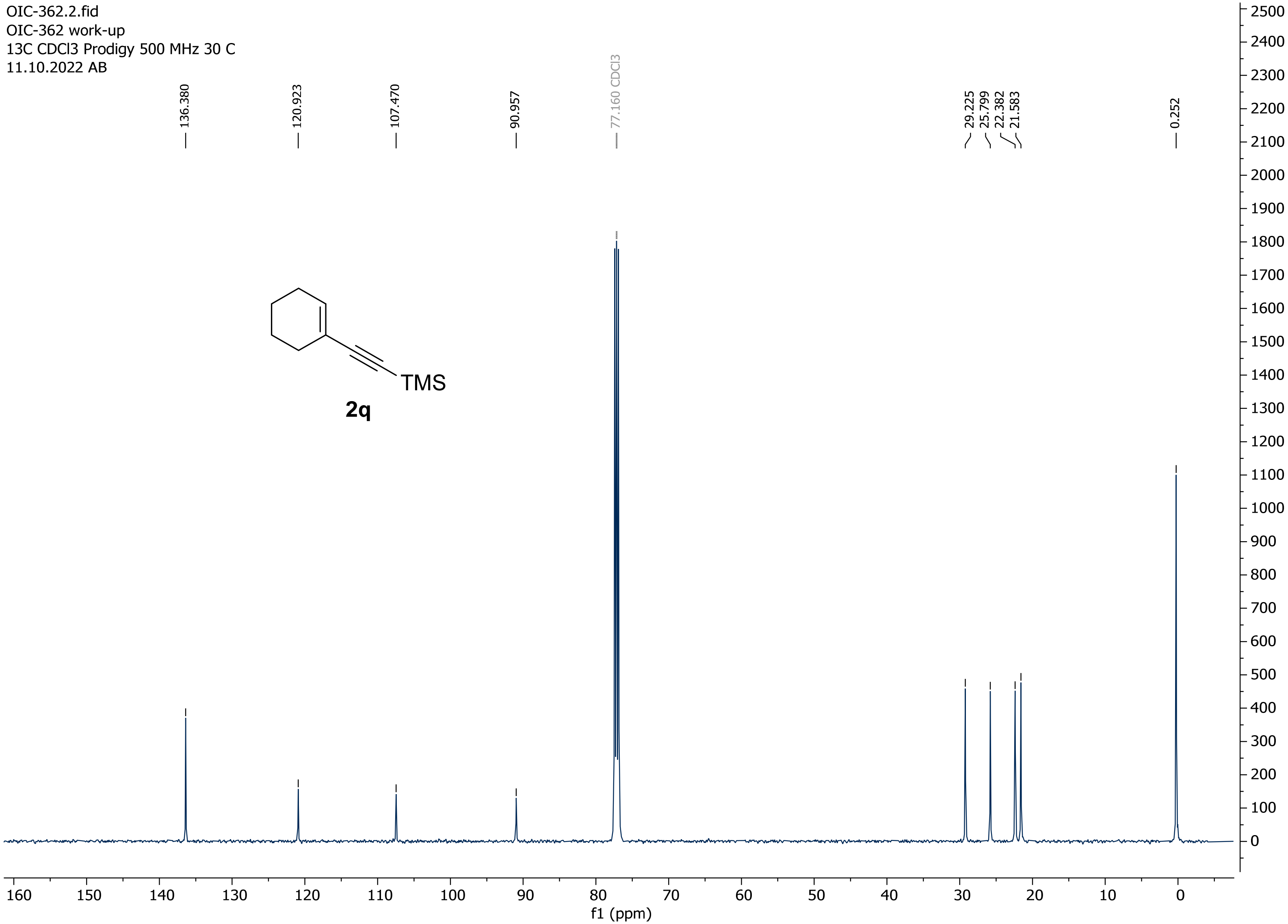

1.35 Hex-1-yn-1-yltrimethylsilane (**2r**), ^{1}H NMR spectrum

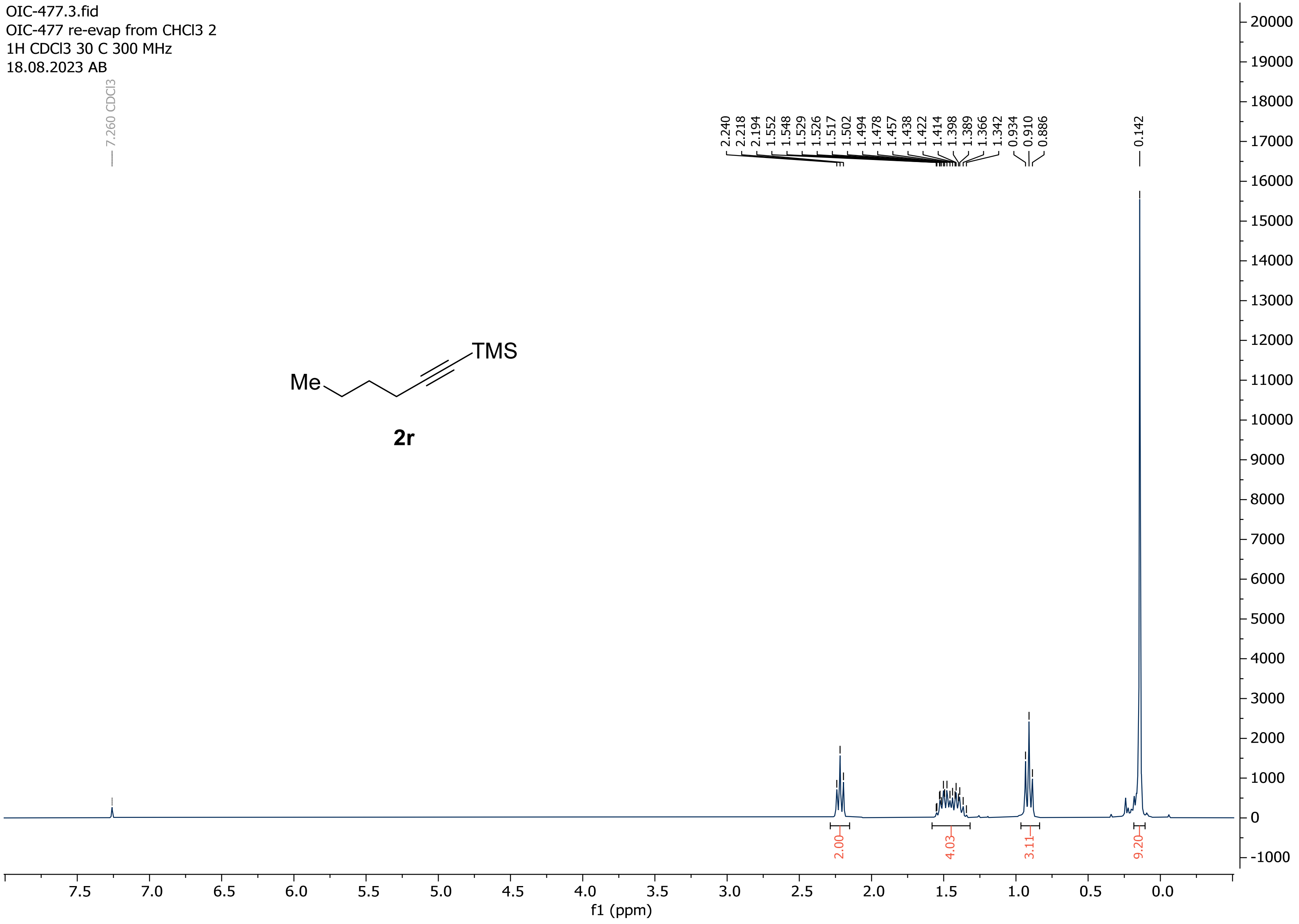

1.36 Hex-1-yn-1-yltrimethylsilane (**2r**), ^{13}C NMR spectrum

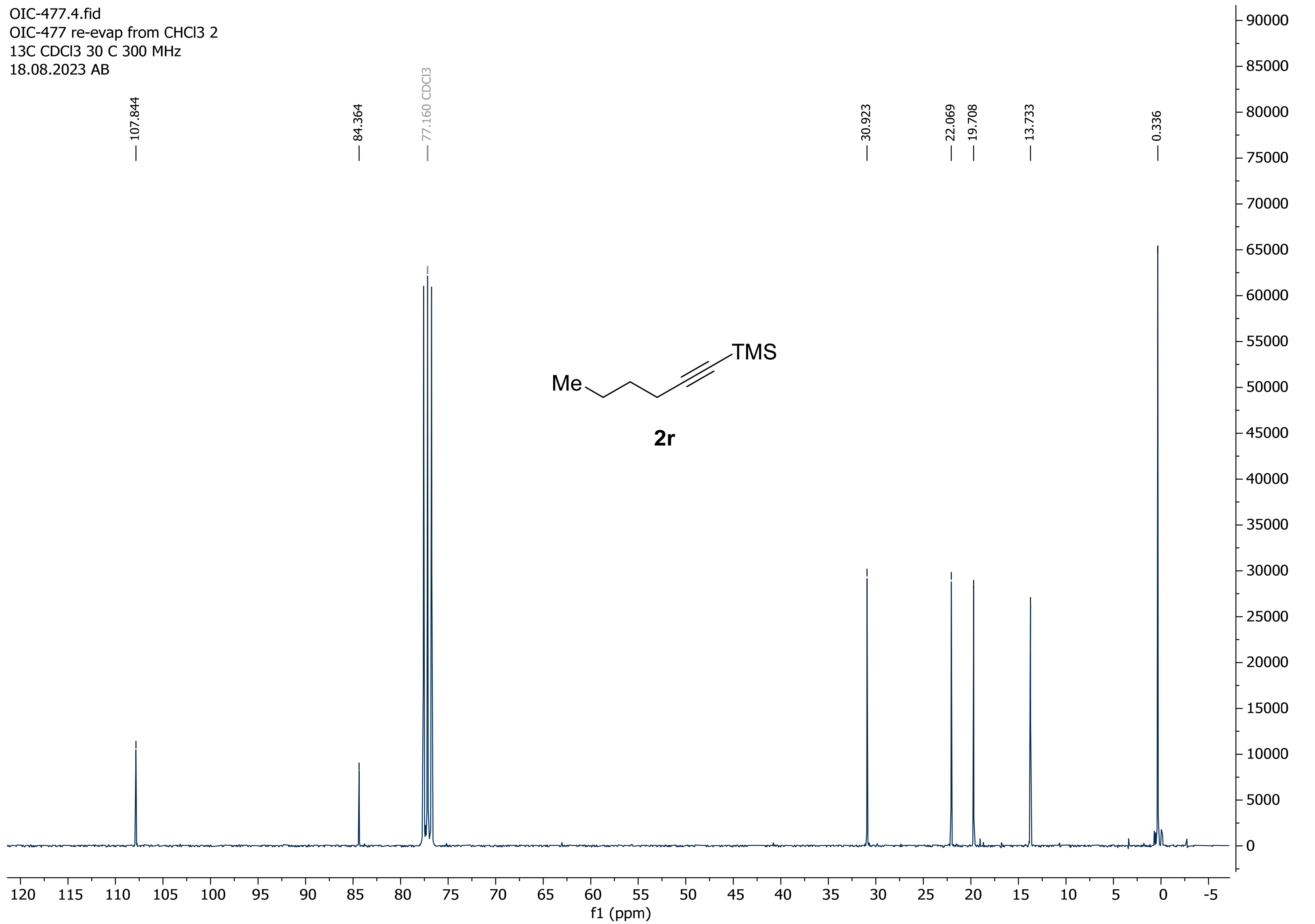

1.37 Trimethyl(4-phenylbut-1-yn-1-yl)silane (**2s**), ^{1}H NMR spectrum

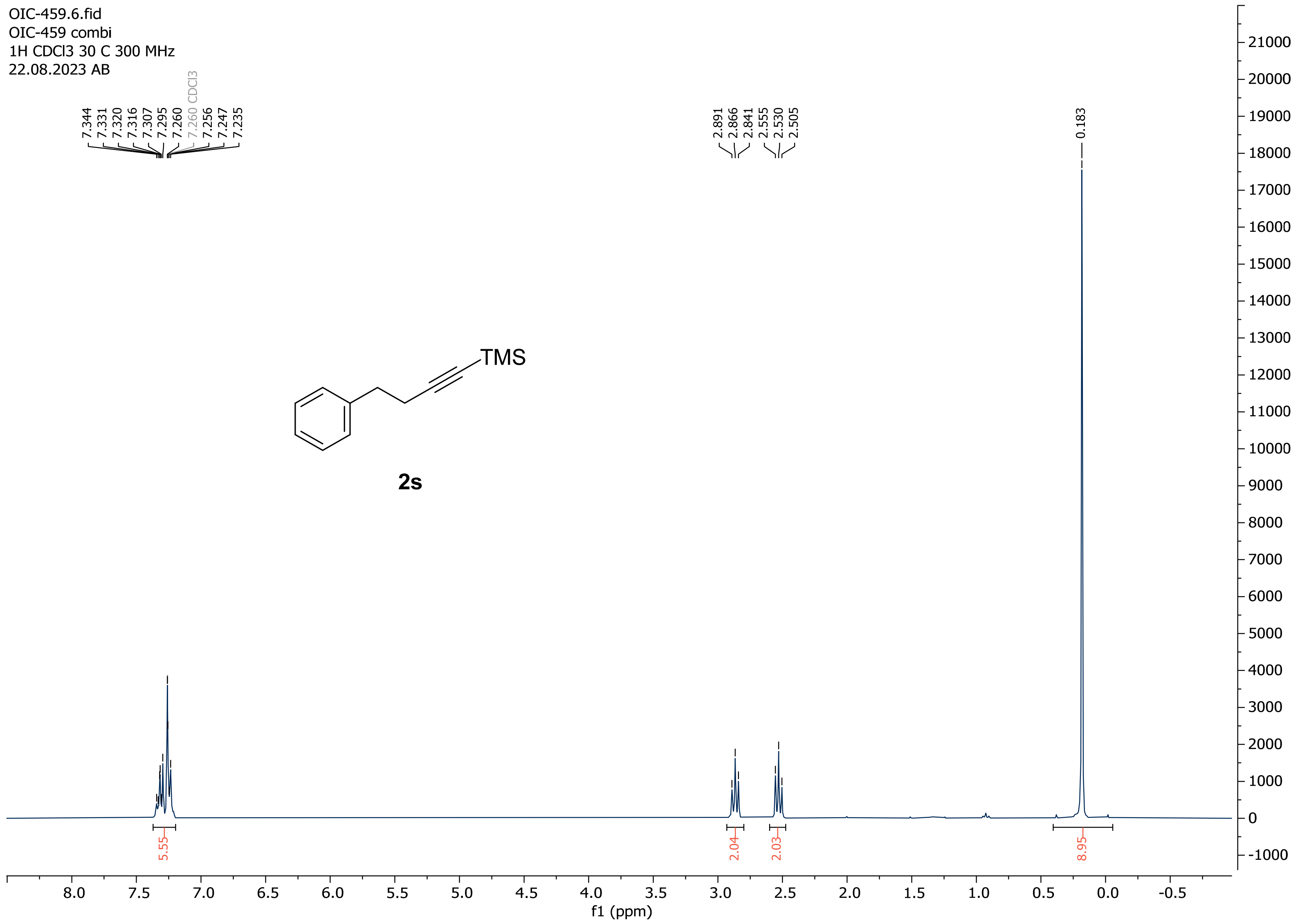

1.38 Trimethyl(4-phenylbut-1-yn-1-yl)silane (**2s**), ^{13}C NMR spectrum

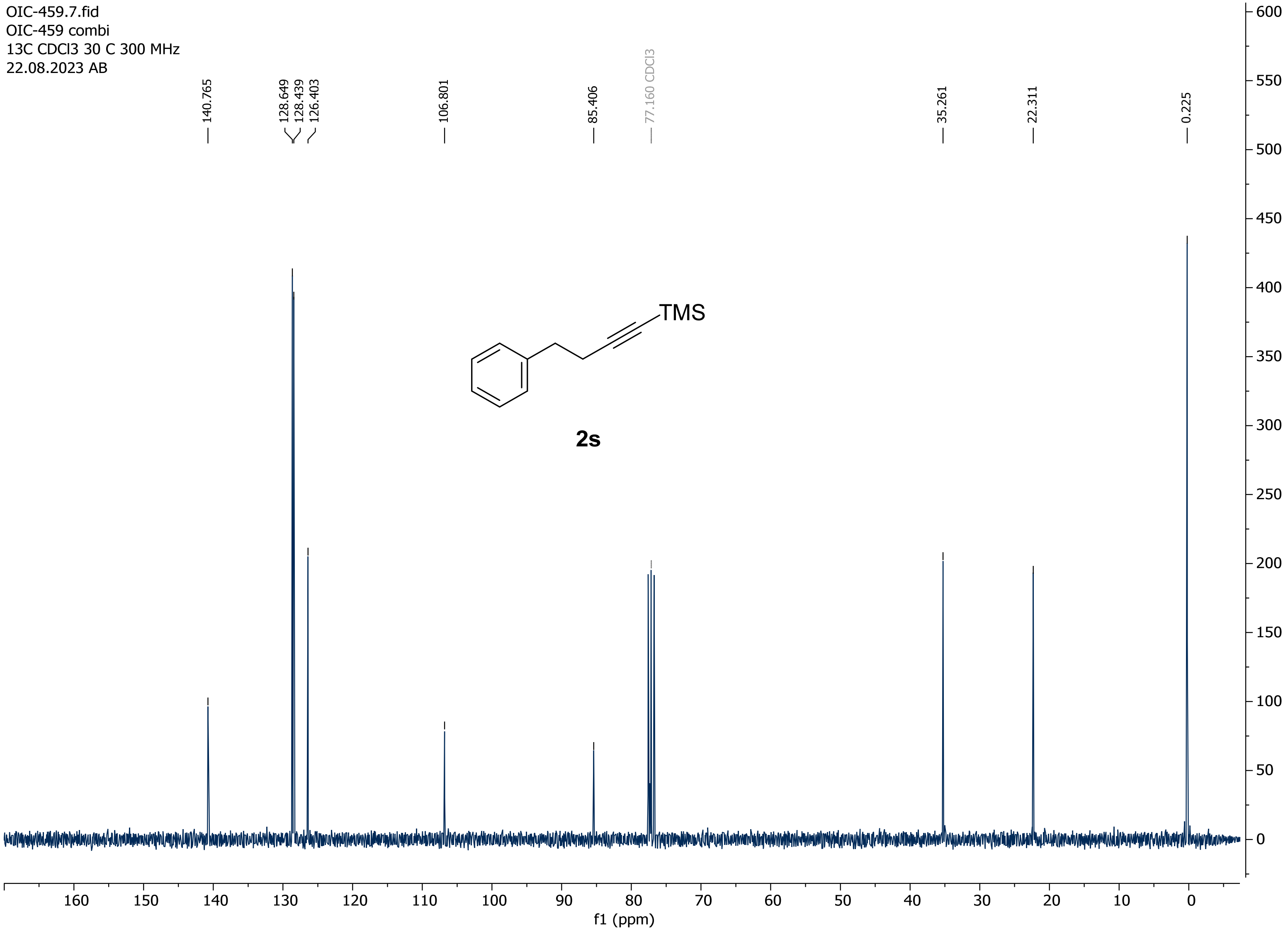

1.39 6-(Trimethylsilyl)hex-5-ynenitrile (**2t**), ^{1}H NMR spectrum

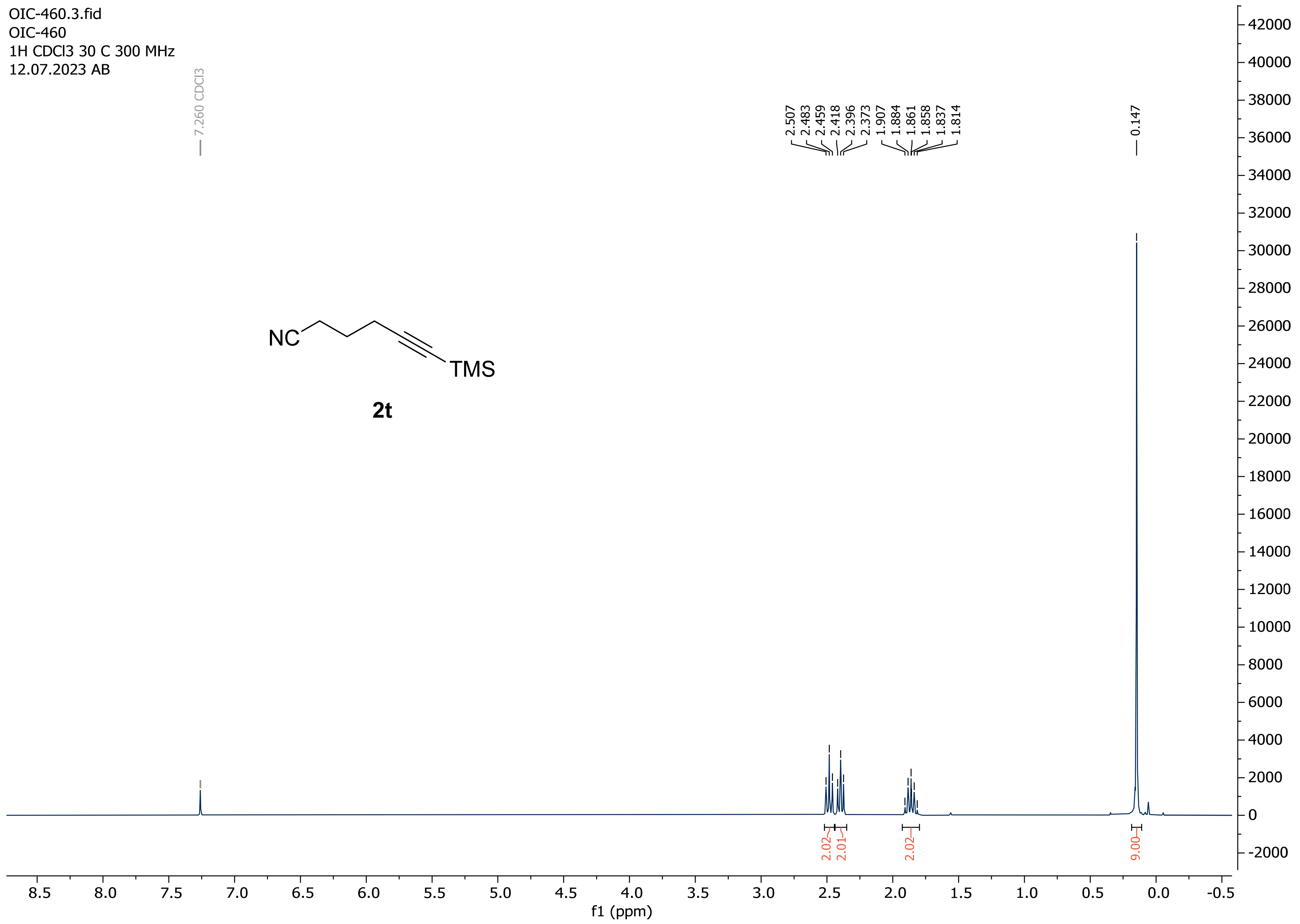

1.40 6-(Trimethylsilyl)hex-5-ynenitrile (**2t**), ^{13}C NMR spectrum

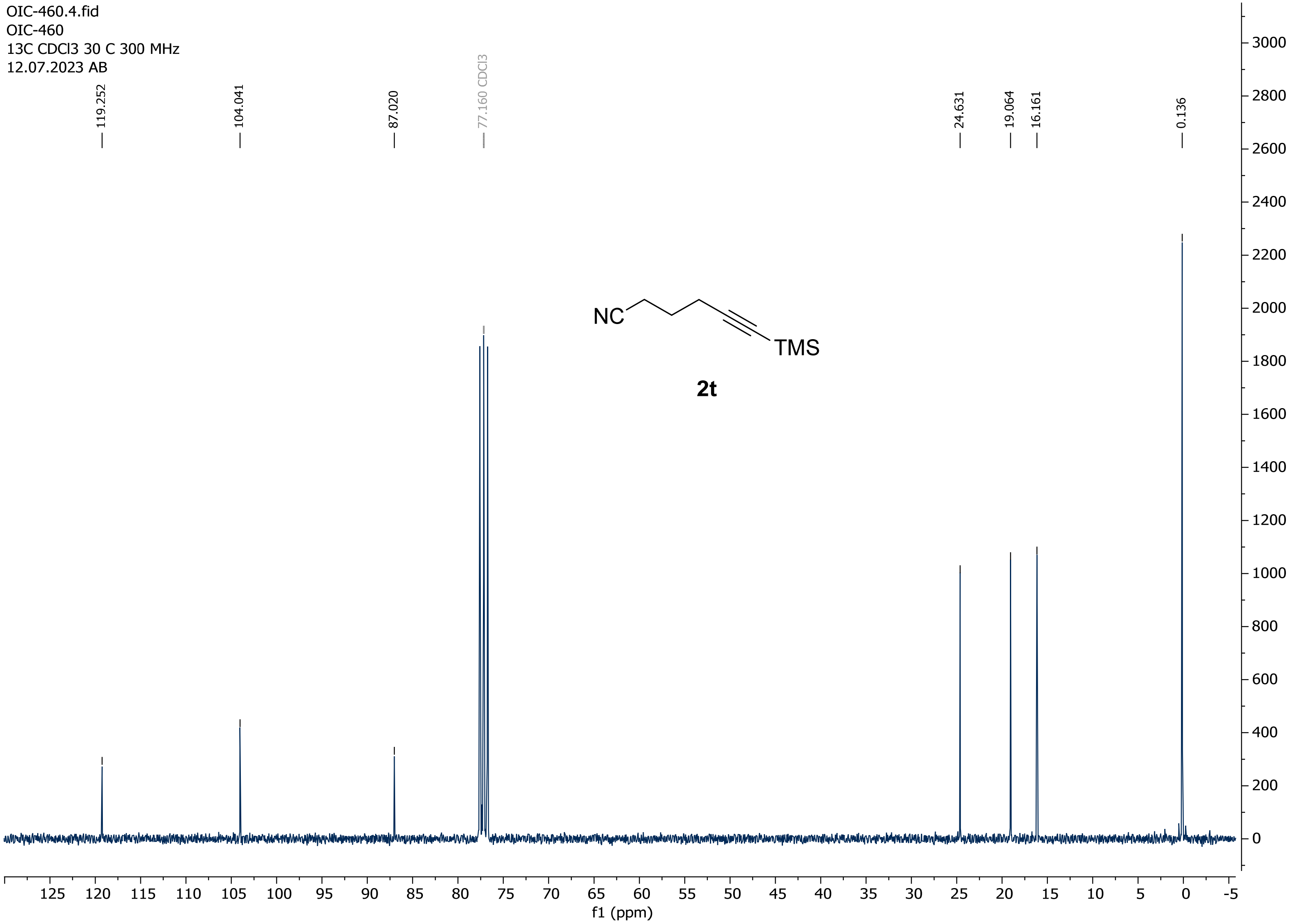

1.41 1,3-Bis((trimethylsilyl)ethynyl)benzene (**2u**), ^{1}H NMR spectrum

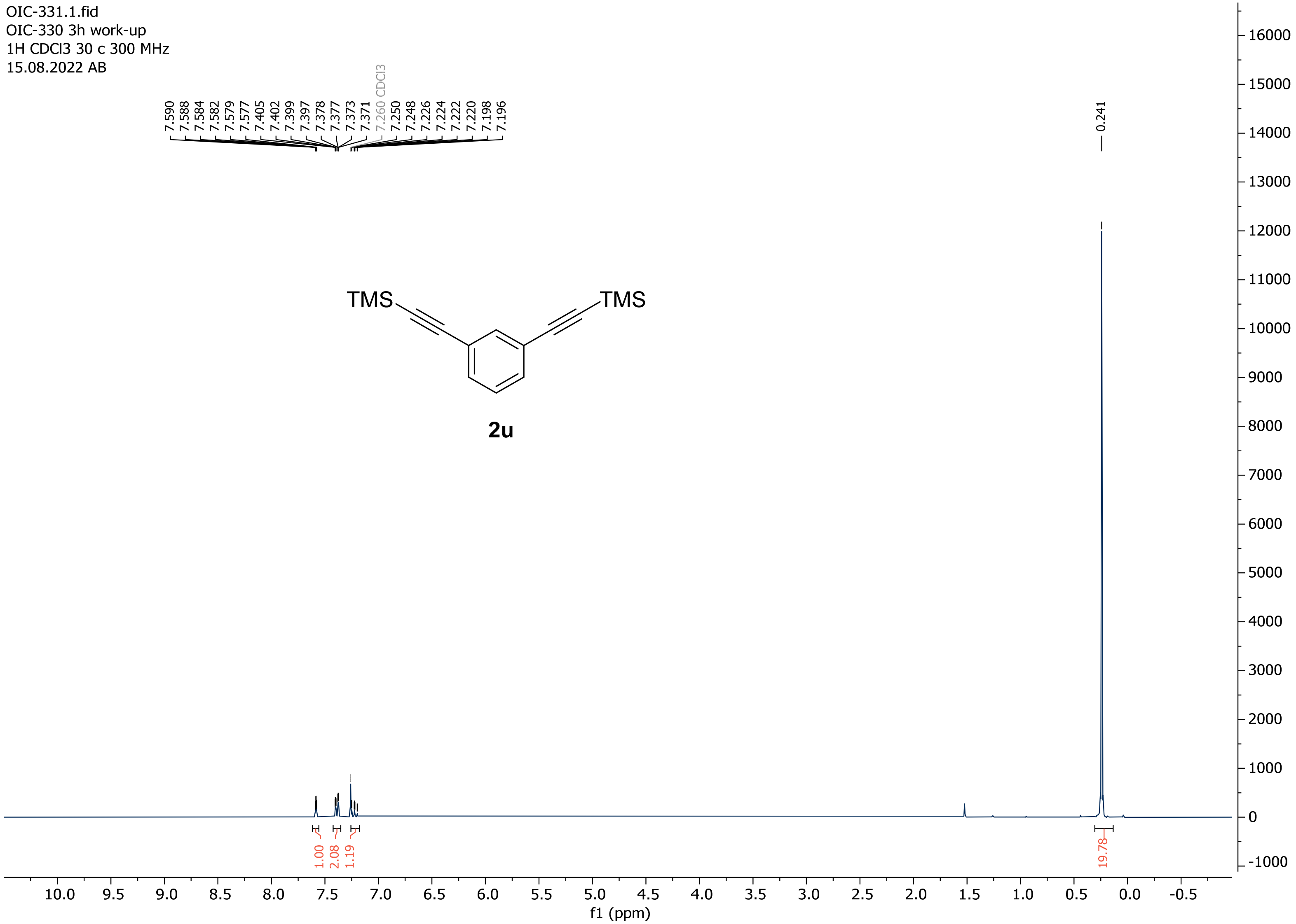

1.42 1,3-Bis((trimethylsilyl)ethynyl)benzene (**2u**), ^{13}C NMR spectrum

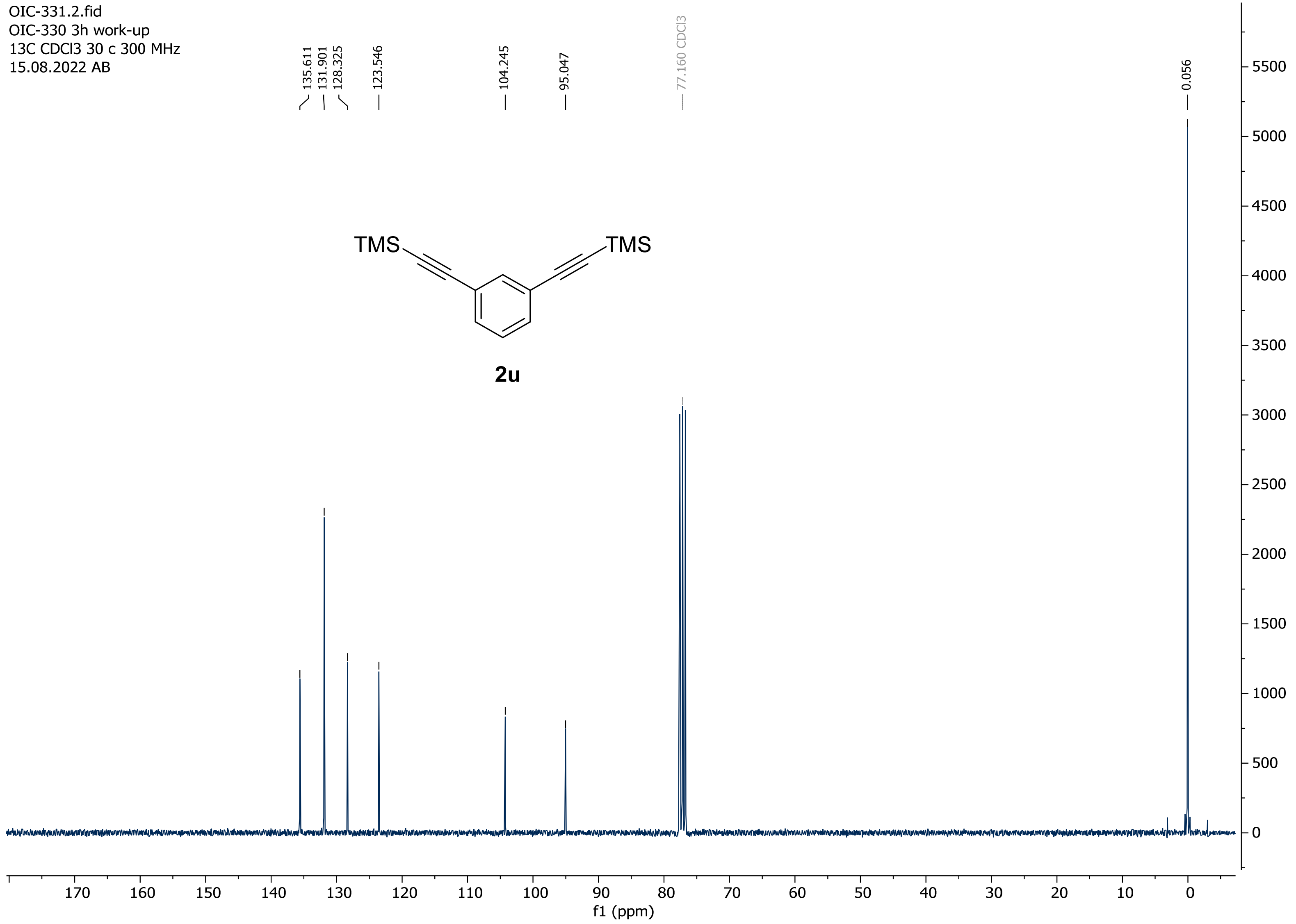

1.43 (((*8R,9S,13S,14S,17S*)-13-Methyl-17-((trimethylsilyl)ethynyl)-7,8,9,11,12,13,14,15,16,17-decahydro-6H-cyclopenta[*a*]phenanthrene-3,17-diyl)bis(oxy))bis(trimethylsilane) (**2v**), ^{1}H NMR spectrum. (*) indicates impurity (bis-*O*-silylated product with free alkyne-C-H).

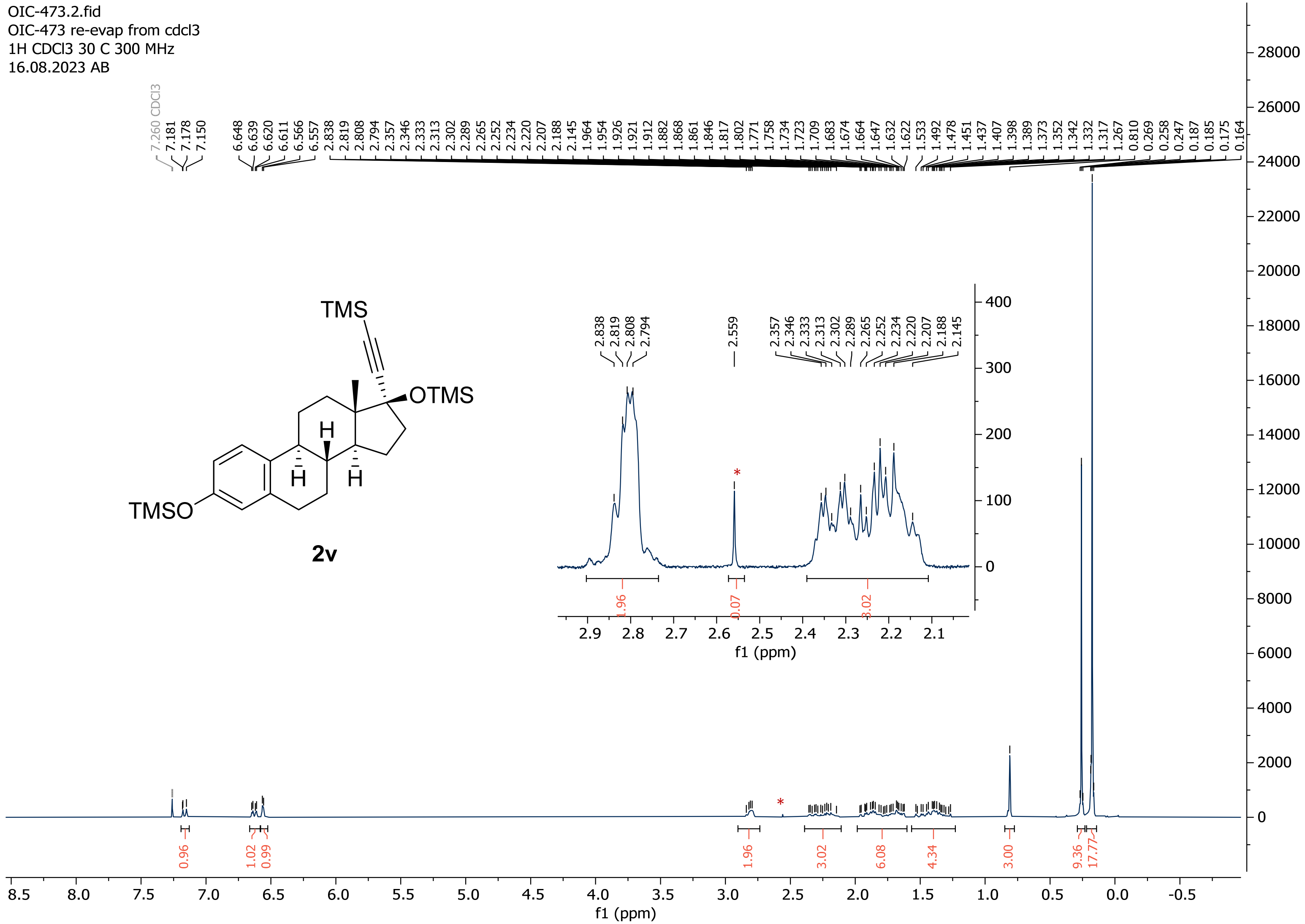

1.44 (((*8R,9S,13S,14S,17S*)-13-Methyl-17-((trimethylsilyl)ethynyl)-7,8,9,11,12,13,14,15,16,17-decahydro-6H-cyclopenta[*a*]phenanthrene-3,17-diyl)bis(oxy))bis(trimethylsilane) (**2v**), ^{13}C NMR spectrum

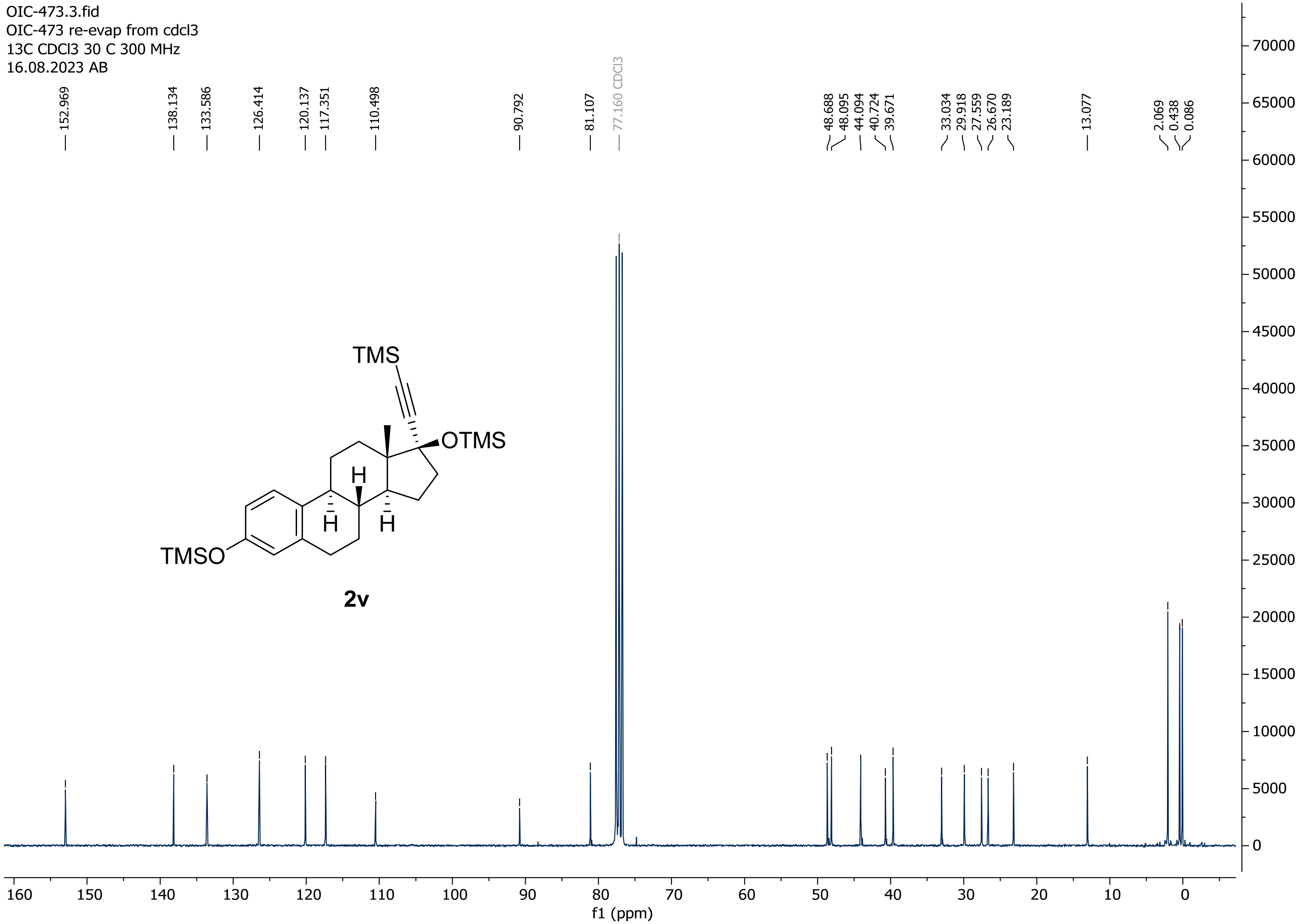

1.45 *tert*-Butyl (trimethylsilyl)(3-(trimethylsilyl)prop-2-yn-1-yl)carbamate (**2w**), ^{1}H NMR spectrum

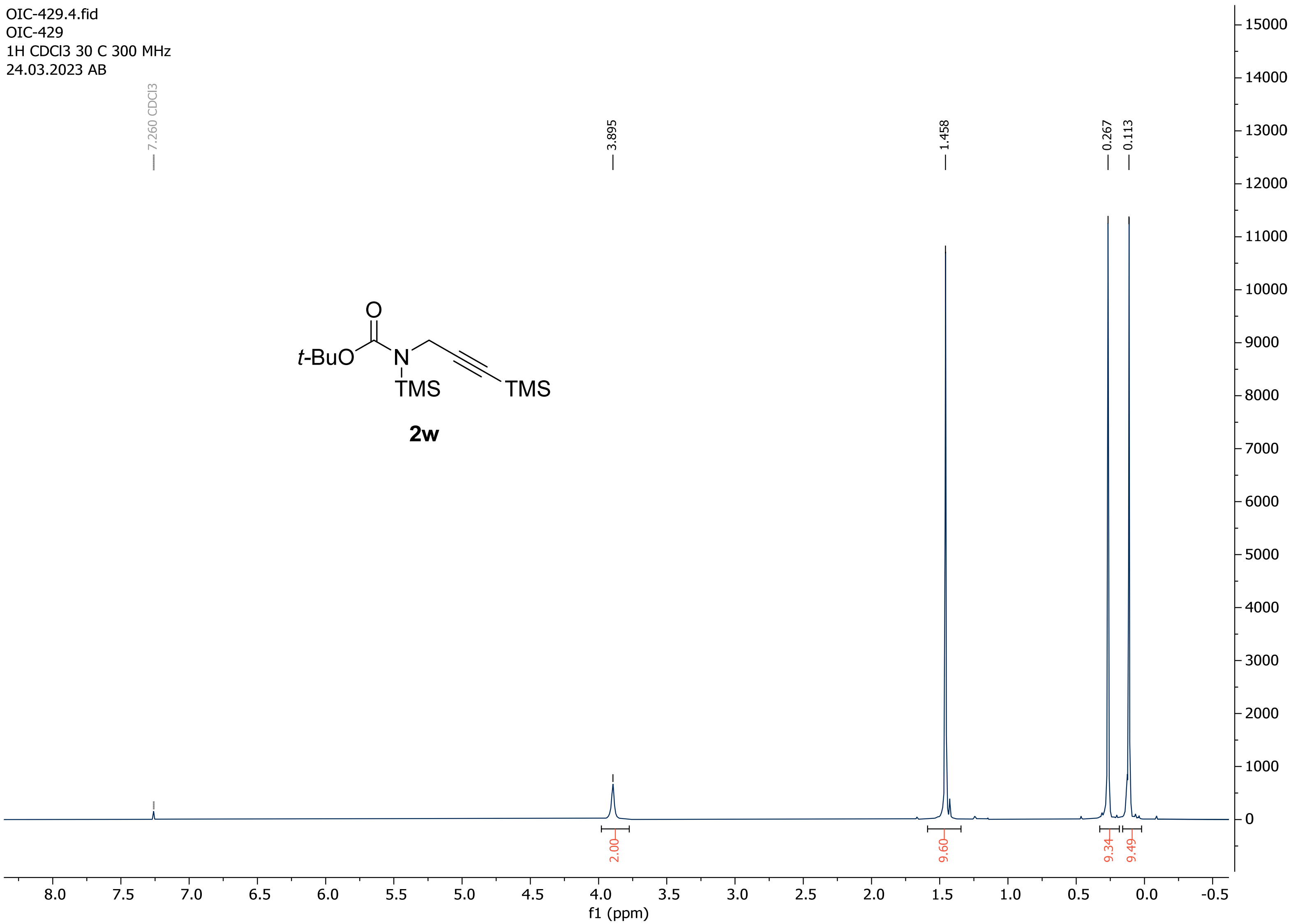

1.46 *tert*-Butyl (trimethylsilyl)(3-(trimethylsilyl)prop-2-yn-1-yl)carbamate (**2w**), ^{13}C NMR spectrum

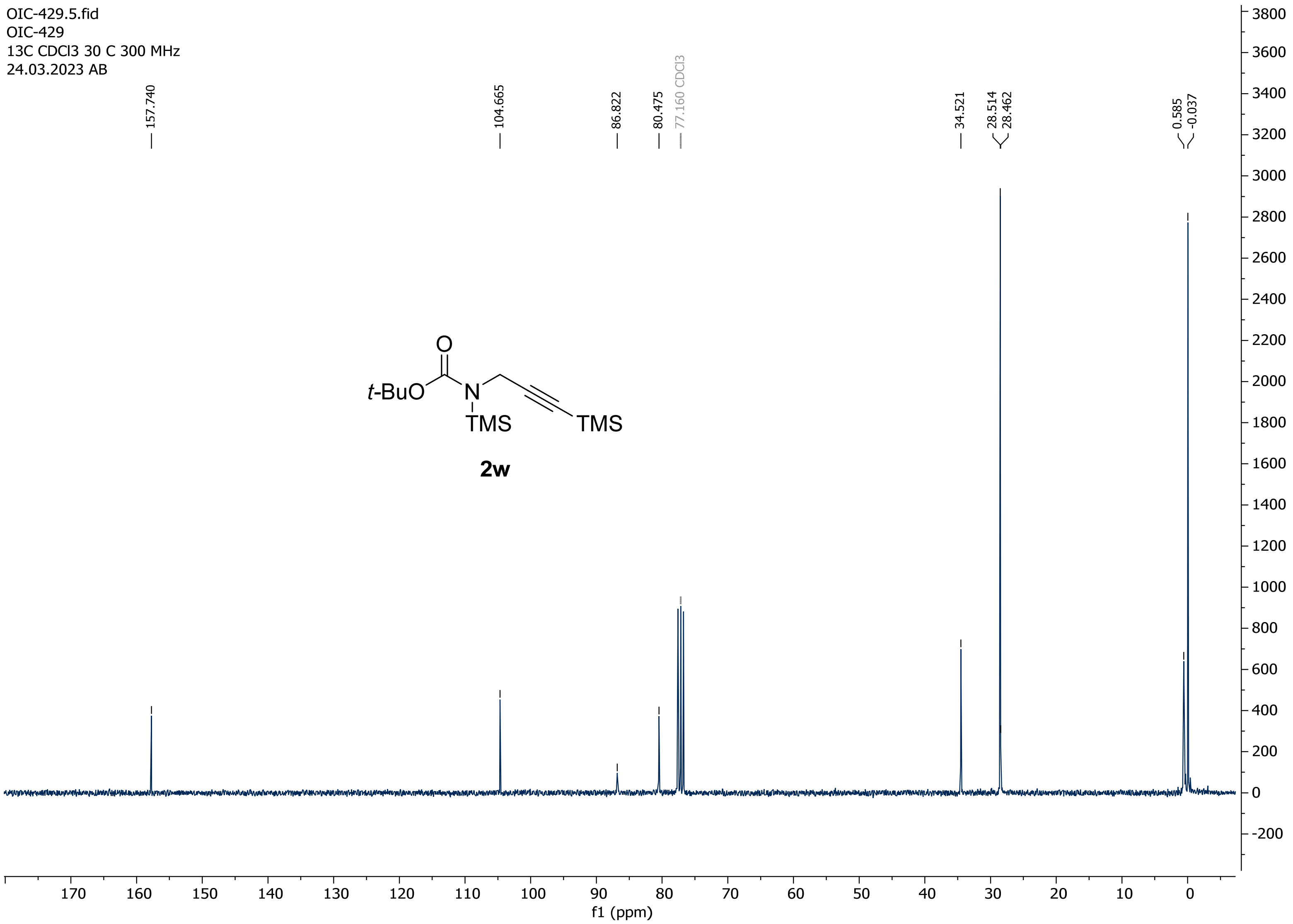

1.47 *tert*-Butyl (3-(trimethylsilyl)prop-2-yn-1-yl)carbamate (**2x**), ^{1}H NMR spectrum

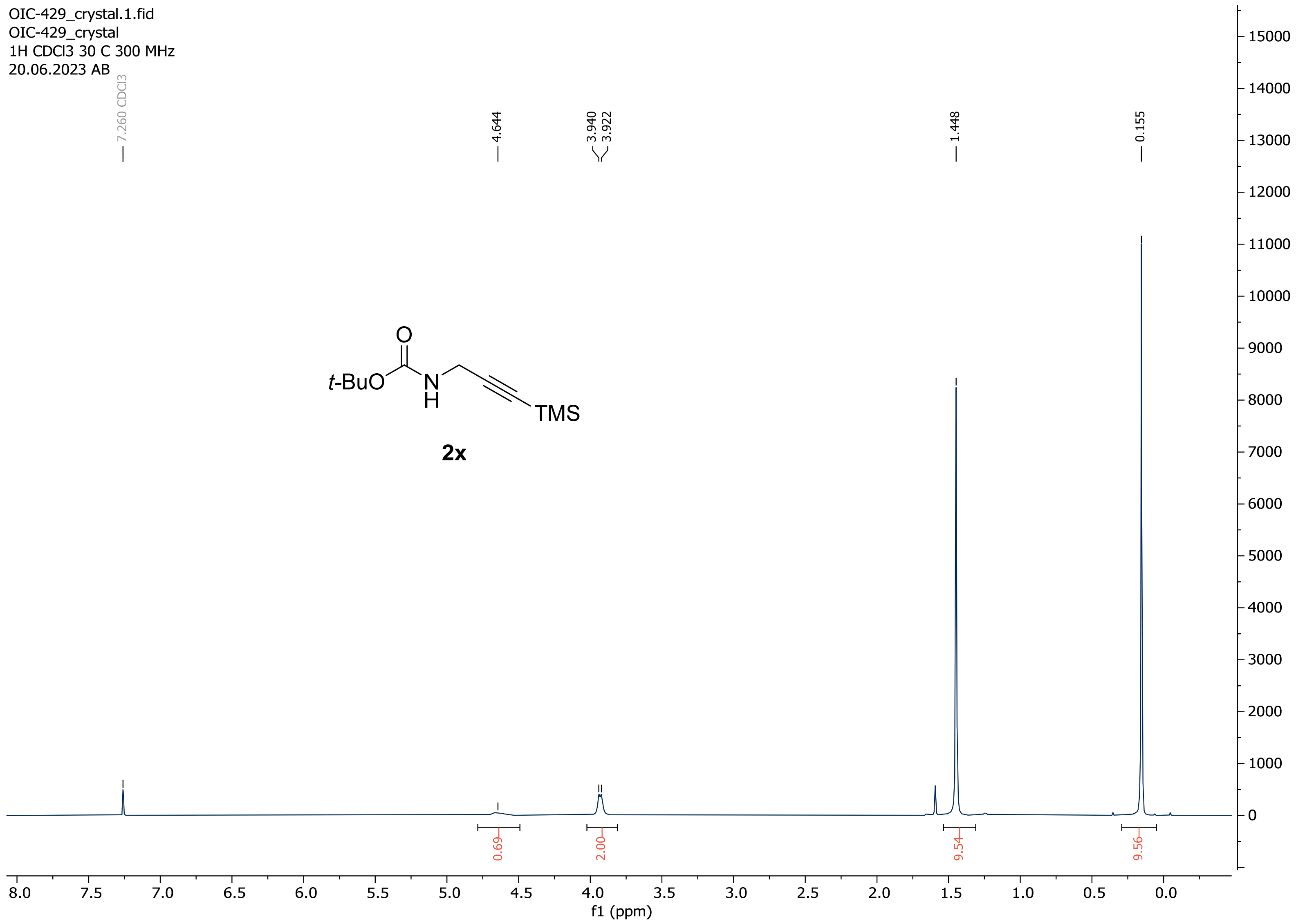

1.48 *tert*-Butyl (3-(trimethylsilyl)prop-2-yn-1-yl)carbamate (**2x**), ^{13}C NMR spectrum

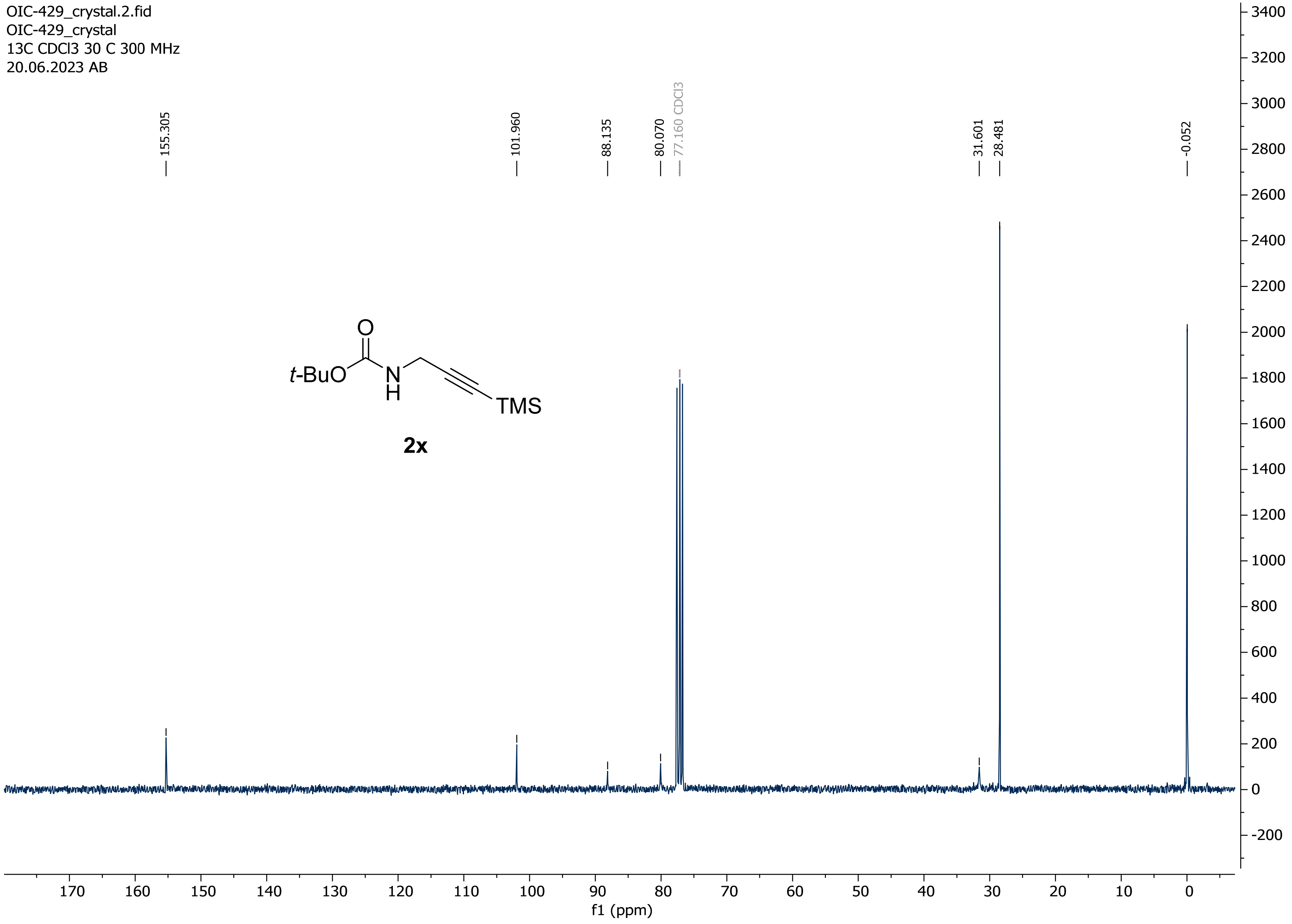

1.49 *tert*-Butyldimethyl(phenylethynyl)silane (**4**), ^{1}H NMR spectrum

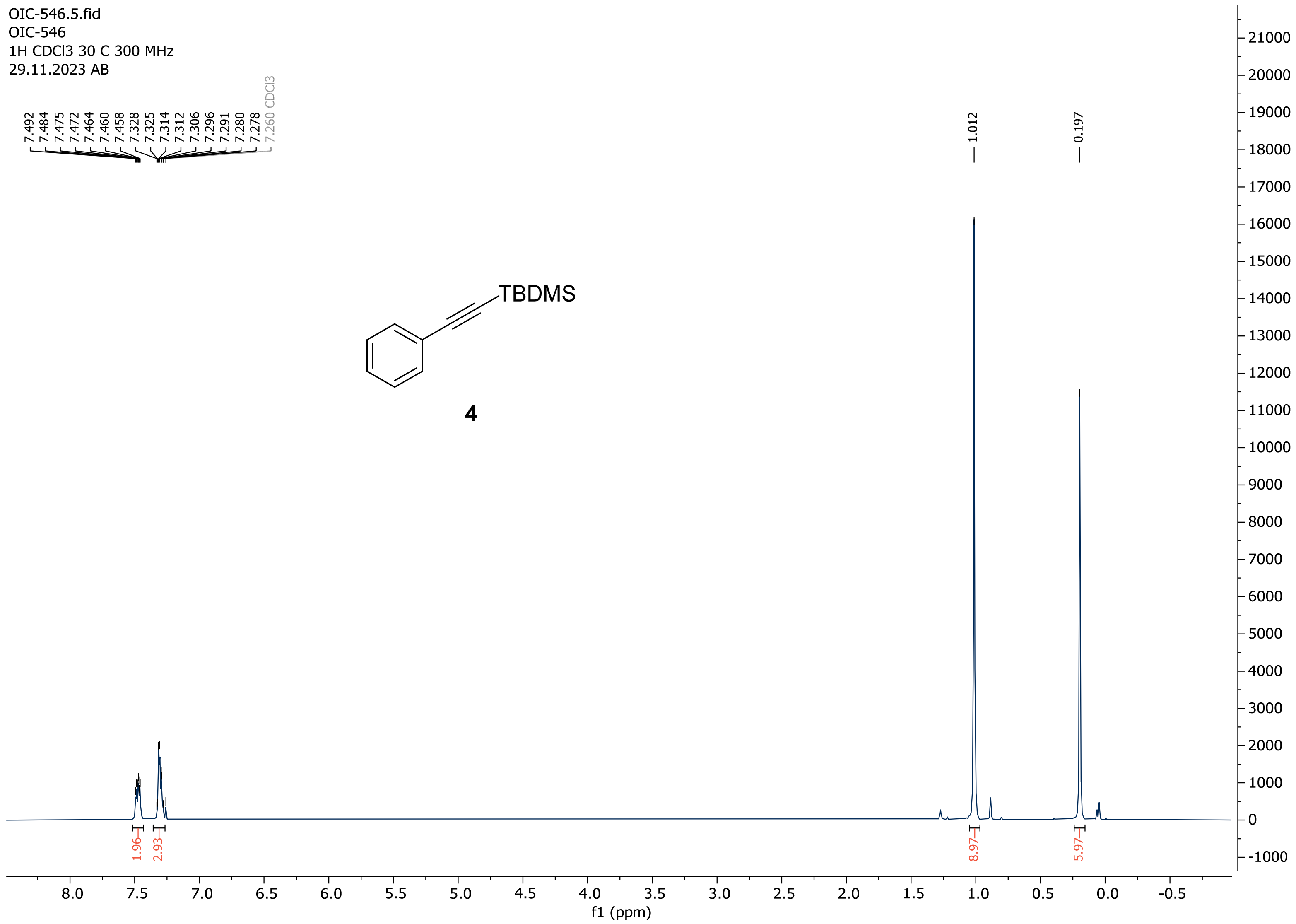

1.50 *tert*-Butyldimethyl(phenylethynyl)silane (**4**), ^{13}C NMR spectrum

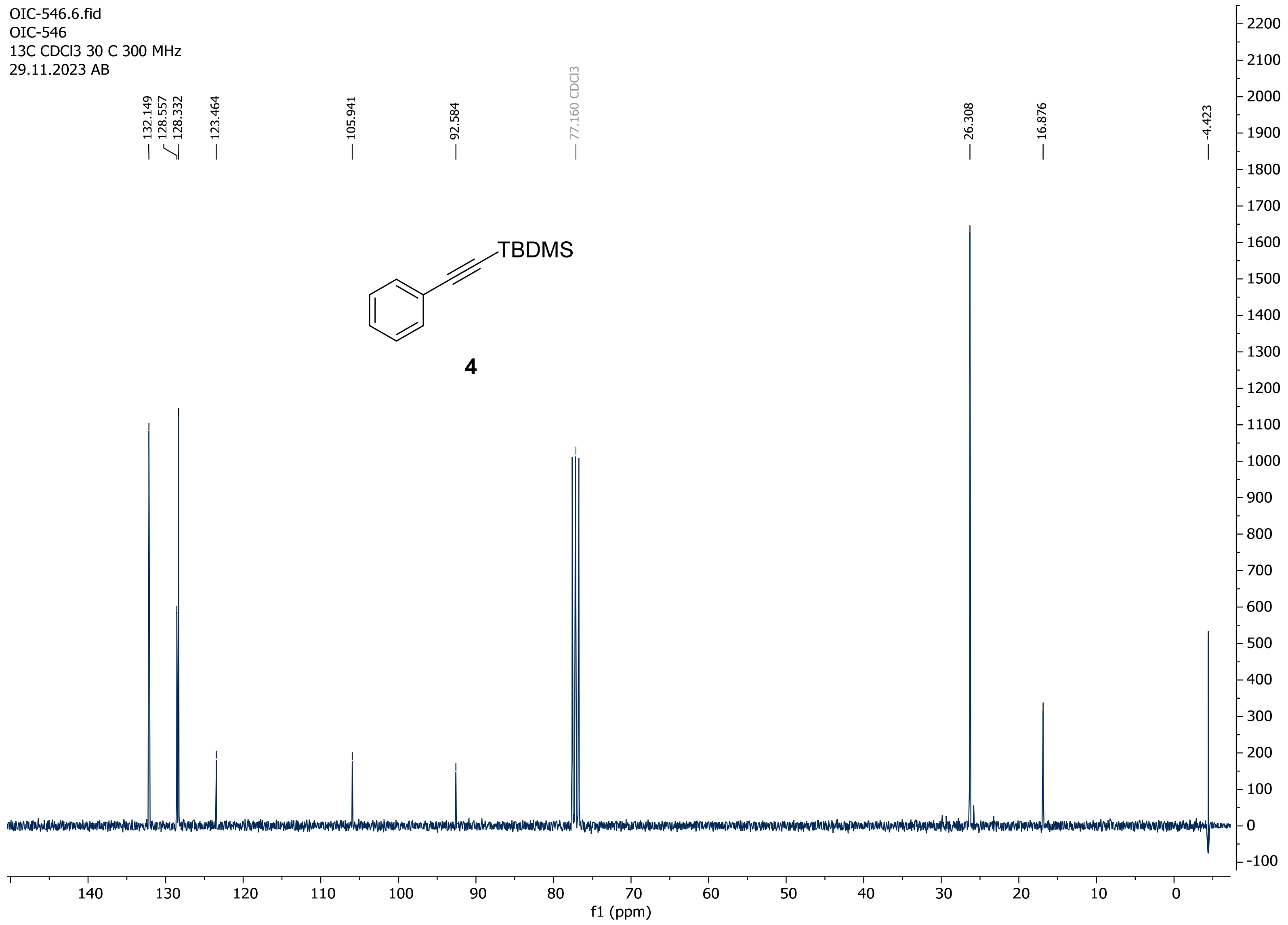

Supplement: Supplementary file 2 — ol3c04213_si_002.pdf [file ol3c04213_si_002.pdf]
