# Supplementary figures and images for: Carboxylate-Catalyzed C-Silylation of Terminal Alkynes
Source: Org Lett. 2024 Mar 1;26(10):1991–5. doi: 10.1021/acs.orglett.3c04213 (PMC10949233; doi:10.1021/acs.orglett.3c04213)

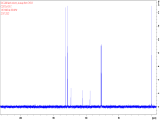

Supplement: Supplementary file 3 — ol3c04213_si_003.zip [file ol3c04213_si_003.zip › NMR Spectra/2a/13C/pdata/1/thumb.png]

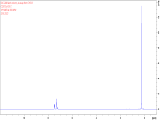

Supplement: Supplementary file 3 — ol3c04213_si_003.zip [file ol3c04213_si_003.zip › NMR Spectra/2a/1H/pdata/1/thumb.png]

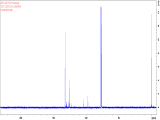

Supplement: Supplementary file 3 — ol3c04213_si_003.zip [file ol3c04213_si_003.zip › NMR Spectra/2b/13C/pdata/1/thumb.png]

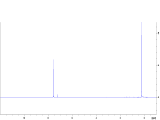

Supplement: Supplementary file 3 — ol3c04213_si_003.zip [file ol3c04213_si_003.zip › NMR Spectra/2b/1H/pdata/1/thumb.png]

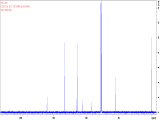

Supplement: Supplementary file 3 — ol3c04213_si_003.zip [file ol3c04213_si_003.zip › NMR Spectra/2c/13C/pdata/1/thumb.png]

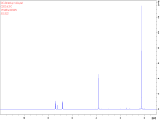

Supplement: Supplementary file 3 — ol3c04213_si_003.zip [file ol3c04213_si_003.zip › NMR Spectra/2c/1H/pdata/1/thumb.png]

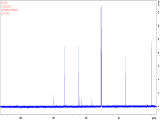

Supplement: Supplementary file 3 — ol3c04213_si_003.zip [file ol3c04213_si_003.zip › NMR Spectra/2d/13C/pdata/1/thumb.png]

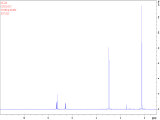

Supplement: Supplementary file 3 — ol3c04213_si_003.zip [file ol3c04213_si_003.zip › NMR Spectra/2d/1H/pdata/1/thumb.png]

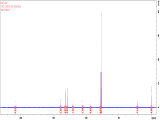

Supplement: Supplementary file 3 — ol3c04213_si_003.zip [file ol3c04213_si_003.zip › NMR Spectra/2e/13C/pdata/1/thumb.png]
